# Engineered signal-coupled inducible promoters: measuring the apparent RNA-polymerase resource budget

**DOI:** 10.1093/nar/gkaa734

**Published:** 2020-09-05

**Authors:** James A Davey, Corey J Wilson

**Affiliations:** Georgia Institute of Technology, School of Chemical & Biomolecular Engineering, 311 Ferst Drive, Atlanta, GA 30332-0100, USA; Georgia Institute of Technology, School of Chemical & Biomolecular Engineering, 311 Ferst Drive, Atlanta, GA 30332-0100, USA

## Abstract

Inducible promoters are a central regulatory component in synthetic biology, metabolic engineering, and protein production for laboratory and commercial uses. Many of these applications utilize two or more exogenous promoters, imposing a currently unquantifiable metabolic burden on the living system. Here, we engineered a collection of inducible promoters (regulated by LacI-based transcription factors) that maximize the free-state of endogenous RNA polymerase (RNAP). We leveraged this collection of inducible promotors to construct simple two-channel logical controls that enabled us to measure metabolic burden – as it relates to RNAP resource partitioning. The two-channel genetic circuits utilized sets of signal-coupled transcription factors that regulate cognate inducible promoters in a coordinated logical fashion. With this fundamental genetic architecture, we evaluated the performance of each inducible promoter as discrete operations, and as coupled systems to evaluate and quantify the effects of resource partitioning. Obtaining the ability to systematically and accurately measure the apparent RNA-polymerase resource budget will enable researchers to design more robust genetic circuits, with significantly higher fidelity. Moreover, this study presents a workflow that can be used to better understand how living systems adapt RNAP resources, *via* the complementary pairing of constitutive and regulated promoters that vary in strength.

## INTRODUCTION

Synthetic biologists strive to design and instantiate useful genetic circuitry and regulatory functions within living biological hosts. These synthetic regulatory functions alter a biological host's ability to respond or adapt to a variety of input (and environmental) stimuli. Example systems include genetically programmable clocks (1), toggle switches (2,3), oscillators (4,5) and logical operations (6–9)—conferring decision making capabilities and regulatory controls orthogonal to those evolved in nature. A sustained effort has been directed toward the engineering of a variety of regulatory components by the synthetic biology community at large, which has enabled the construction of increasingly complicated genetic circuit assemblies. However, the expression of introduced synthetic genetic systems, relying on endogenous transcription and translation machinery, imparts a significant burden on the biological host's ability to maintain homeostasis (10). This challenge has also emerged as a problem associated with the routine scale-up of industrial biotechnological processes (11). Furthermore, this problem complicates the comparable performance and transfer of these systems between biological hosts because the experiments are sensitive to changes in their genetic design and growth conditions (12–16). It is anticipated that future constructs, requiring the simultaneous expression of multiple interacting regulatory components, will be rendered increasingly susceptible to expression burden. Thus, it is expected that problems arising from expression burden will occur with greater incidence and severity as the complexity of synthetic biological circuitry increases (17–20).

Biological hosts coordinate their homeostasis with a variety of cellular factors and environmental cues by stringently regulating levels of endogenous RNA polymerase (RNAP) (21), ribosomes (22,23), and their associated components (e.g. sigma factors modulating rate of RNAP recruitment to specific promoter sequences) (14,24,25). This regulation has evolved to enable the efficient allocation of resources between an organism's growth and maintenance needs (10). Presumably, expression of introduced synthetic biology gene products is limited and demarcated as a consequence of this resource and component balance (26). This balance, referred to as a resource budget, consists of ∼1500–11 400 RNAP molecules and 6800–72 000 ribosomes per cell (27), which vary according to the growth phase of the cell and environmental conditions such as available nutrients and temperature (11). Many synthetic biological systems are implemented using *Escherichia coli* as a biological host. Homeostasis requires that this resource budget must be distributed across *E. coli's* ∼4000 endogenous genes (16) and balanced against the burden of expressing any introduced synthetic biological constructs. As a result, the function of programmable genetic circuitry can be unstable, and variable due to coupled expression of synthetic biological gene constituents and resources required to maintain homeostasis of the host (28). The complexity of this problem has led to the creation of many expression models that ignore changes to the resource budget, effectively treating RNAP and ribosome as spectator molecules (3,5,26). However, depletion of the resource budget has been demonstrated to have systemic consequences, evidenced by observation of reduced expression of introduced constructs or impacted homeostasis that results from molecular constituents competing for RNAP or ribosome (29,30). To reduce competition for host homeostatic factors, molecular biologists have introduced orthogonal components from bacteriophage, such as T7 RNAP, to drive transcription of introduced constructs (31). To further mitigate this issue, Segall-Shapiro *et al.* implemented a landmark design for a resource allocation using fragmented T7 RNAP (32). While this approach successfully helps to reduce depletion of the host resource budget, the fragmented components must be expressed by endogenous host machinery. If ever increasing complex synthetic biological circuits are to be implemented, a readily implemented and simple means for quantification and the predictable modeling of expression burden is desirable (26).

With this objective in mind, we sought to construct a methodology that would enable quantification and interpretation of expression burden to be implemented by molecular and synthetic biologists alike. This system functions by measuring differential expression of super-folder green fluorescent protein (GFP) (33) and mCherry (RFP) (34) donated on an in-house assembled dual reporter gene plasmid. The reporter plasmid was co-expressed alongside a single regulatory component (lac repressor, LacI) donating a plasmid containing a copy of the functional repressor (+ LacI) or non-functional repressor (–LacI). A library of reporter plasmids provides a combination of constitutive, inducible, and absent promoter architectures regulating transcription of GFP and RFP. This library enabled quantification of resource competition when reporter protein expression was assayed over a time course, in the absence and presence of the inducer molecule isopropyl β-d-1-thiogalactopyranoside (IPTG). This setup readily facilitates the comparative measurement of expression output for a given reporter protein as a function of burden introduced by a competing reporter. When employing our coupled reporter constructs we observed linear depression of inducible (pLac) RFP expression, even when paired with the weakest synthetic promoter sequence (pUV5) on GFP. Next, we examine how promoter strength and repressor response changes as a function of inducer concentration titration and burden. Finally, we evaluated the data collected in this study in aggregate to illustrate the impact of resource partitioning on dual promoter systems – relative to putative synthetic biology performance boundaries.

## MATERIALS AND METHODS

### Construction of the reporter plasmid scaffold

Study of promoter competition for endogenous transcriptional resources required the construction of a reporter plasmid expressing the two reporter genes, super folder green fluorescent protein (GFP) (33) and the red fluorescent protein mCherry (RFP) (34). The pZS*22-sfGFP plasmid (ExpresSys) previously employed by Richards *et al.* provided the base scaffold from which all reporter plasmids were constructed (35). All reporter plasmids contain the pSC101* replication origin resulting in a low cellular plasmid copy number (estimated 3 – 5 copies per cell) and express the selection marker conferring resistance to kanamycin (effective kanamycin working concentration 100 μg·ml^−1^), donated from pZS*22-sfGFP. The promoter of the GFP gene was deleted by quick change mutagenesis (36), by PCR with deep vent (New England Biolabs, NEB) followed by DpnI (NEB) digestion. This reaction was subjected to PCR clean-up (Qiagen) and transformed into in-house prepared electro-competent 3.32 cells (37). A transformant was cultured overnight (37°C with shaking 300 rpm) in LB with kanamycin (100 μg·ml^−1^) and subjected to miniprep (Omega Bio-tek, Omega) to yield an intermediate plasmid incapable of GFP expression. This intermediate plasmid served as the vector for insertion of the RFP gene. The gene for RFP was isolated from the pET28b plasmid, kindly provided by the Kane laboratory, by PCR amplification with deep vent (NEB) and gel extraction (Qiagen). The rrnB T1 terminator (38) flanking the GFP gene in pZS*22 was amplified by PCR with deep vent (NEB) and gel extracted (Qiagen). The RFP gene was prepared for insertion by SOE PCR (39) using deep vent (NEB) to produce a RFP·rrnB T1 insert lacking a promoter cassette. The RFP·rrnB T1 PCR was purified (Qiagen) and used to replicate a linearized (NEB) and gel extracted (Qiagen) copy of the intermediate plasmid by circular polymerase extension cloning (CPEC) (40) using deep vent (NEB). The resulting reaction product was purified by PCR clean-up (Qiagen) and transformed into the electro-competent 3.32 cells to yield the –pGFP/−pRFP reporter plasmid scaffold following miniprep (Omega). As expected, spectrophotometry, conducted using a SpectraMax M2e plate reader (molecular devices), of 3.32 cultures transformed with the –pGFP/−pRFP reporter plasmid confirmed that this reporter plasmid was incapable of GFP (λ_ex_ = 485 nm, λ_em_ = 510 nm, gain = 400) or RFP (λ_ex_ = 585 nm, λ_em_ = 610 nm, gain = 400) expression. Sequencing (Eurofins Genomics) confirmed the presence of both GFP·rrnB T1 and RFP·rrnB T1 constructs in the –pGFP/−pRFP reporter plasmid.

### Construction of the promoter cassettes and their insertion into the reporter plasmid

Promoter cassettes to be inserted upstream and adjacent to the start codons of the GFP·rrnB T1 and RFP·rrnB T1 constructs were constructed by polymerase cycling assembly (PCA) (41,42) using deep vent (NEB) and purified by gel extraction (Qiagen). Each promoter cassette is comprised of an identical 60 BP region immediately upstream of the promoter and an identical 115 BP region immediately downstream of the promoter, containing the genetic insulator RiboJ (43), and the ribosome binding site enabling translation of both GFP and RFP mRNA transcripts. GFP and RFP promoter cassettes vary according to the 5′ and 3′ termini presenting complementary sequences with their site of insertion on the –pGFP/−pRFP scaffold and their respective fluorescent protein genes. Each promoter cassette was constructed to deliver a variable promoter sequence to each fluorescent reporter protein. Promoter sequences inserted adjacent to GFP·rrnB T1 include the constitutive promoters pUV5 (44), pNull, and pTrc (45), as well as the regulated promoters pSym (46), pLac (47), and pTTA, recognized by the lac repressor DNA binding domains YQR (Y17/Q18/R22), YQR, and IAN/TAN/VAN, respectively. Of the promoter elements, pUV5, pTrc, pSym and pLac were all previously published. The promoter elements pNull and pTTA were designed by sequence alignment against the pLac and pSym promoters, respectively. Prior to their insertion into the reporter plasmid, promoter cassettes were adapted with a portion of their intended fluorescent protein gene by SOE PCR (39) using deep vent (NEB) and gel extracted (Qiagen). These promoter fused reporter protein constructs were then inserted into the –pGFP/−pRFP reporter plasmid by CPEC (40) using Phusion polymerase (NEB) to produce reporter plasmids with a single transcribable copy of either GFP or RFP. Promoter cassettes introduced into the –pGFP/−pRFP reporter plasmid include pUV5, pSym, pNull, pLac and pTrc controlling expression of GFP, and pTTA, pNull, and pLac, controlling expression of RFP. Each CPEC reaction was purified by PCR cleanup (Qiagen) and transformed into in-house electro-competent 3.32 cells. Transformants were screened by visual inspection and eight colony plaques were selected for spectrophotometric characterization to ensure that transformants expressed their intended GFP (λ_ex_ = 485 nm, λ_em_ = 510 nm, gain = 400) or RFP (λ_ex_ = 585 nm, λ_em_ = 610 nm, gain = 400) reporter protein. Plasmids belonging to transformants displaying their expected phenotype were extracted by miniprep (Omega) from overnight cultures (37°C with shaking 300 rpm). The insert region of these plasmids were sequenced (Eurofins Genomics) to confirm the presence of the intended promoter cassette and fluorescent protein. This process of CPEC, phenotype characterization, and sequencing was repeated using the –pGFP/pLac RFP plasmid to insert the series of GFP adapted promoter cassettes producing reporter plasmids with pUV5, pSym, pNull, pLac, and pTrc controlling expression of GFP simultaneously with pLac controlling expression of RFP. The reporter plasmids: pLac GFP/pNull RFP, pNull GFP/pTTA RFP and pTrc GFP/pTTA RFP were also produced.

### Construction of the LacI donor plasmids

The 3.32 cells used in this study contain the mutation lacI22, thus lacking a functional genomic copy of LacI required to repress gene transcription via interaction with lac type operators. Instead, functional copies of the LacI gene are donated on a separate plasmid, referred to as pLacI. The plasmid pLacI contains a chloramphenicol resistance gene (effective chloramphenicol working concentration 35 μg·ml^−1^) and the p15A origin of replication (estimated 20–30 copies per cell). Five LacI genotypes were employed in this study, including the functional wild-type repressor (+LacI), a copy of the LacI gene with a stop codon mutation in place of the first residue of the repressor incapable of gene translation (−LacI), as well as three DNA binding domain variants: LacI_Y17I/Q18A/R22N_ (+IAN), LacI_Y17T/Q18A/R22N_ (+TAN) and LacI_Y17V/Q18A/R22N_ (+VAN) (48). Plasmids +LacI and –LacI originate from a previous study (35), while +IAN, +TAN and +VAN were produced by quick change mutagenesis (36) of the +LacI gene using deep vent (NEB) and DpnI (NEB) digestions. Plasmids produced by quick change were purified (Qiagen), transformed into 3.32 electro-competent cells, and used to inoculate cultures grown overnight (37°C with shaking 300 rpm) for miniprep (Omega). Harvested plasmids were subjected to sequencing (Eurofins Genomics) to confirm their identity.

### *In vivo* assaying of reporter expression activity

All reporter plasmids were co-expressed with one of two pLacI plasmids donating either a functional copy of the lac repressor (+LacI) or a non-translatable gene (–LacI). Co-expression required co-transformation in 3.32 cells by electroporation followed by selection on LB agar spiked with 100 μg·ml^−1^ of kanamycin and 35 μg·ml^−1^ of chloramphenicol. Three colony forming units from each co-transformation were selected to inoculate three 1 ml cultures of LB and grown overnight (37°C with shaking 500 rpm) maintaining selection with antibiotics (kanamycin = 100 μg·mL^−1^, chloramphenicol = 35 μg·ml^−1^). The density of each culture was recorded by spectrophotometry (λ_abs_ = 600 nm) and diluted to 0.01 absorbance units using sterile deionized H_2_O. 12 ul volumes of diluted cultures were passaged into 96 deep well microplates containing 1.2 ml volumes of M9 minimal media supplemented with cas amino acids, antibiotics to maintain selection (kanamycin = 100 μg·ml^−1^, chloramphenicol = 35 μg·ml^−1^), and the absence or presence of 10 μM IPTG. Microplates were grown for a total of 26 h (37°C with shaking 500 rpm), withdrawing 100 ul of culture for assaying starting at the 12th hour, in 2 h intervals. Cultures were assayed to determine their density (λ_abs_ = 600 nm), GFP expression (λ_ex_ = 485 nm, λ_em_ = 510 nm, gain = 400), RFP expression (λ_ex_ = 585 nm, λ_em_ = 610 nm, gain = 400) and FRET (λ_ex_ = 485 nm, λ_em_ = 610 nm, gain = 400). IPTG titration curves were conducted by repeating the assay workflow, in the presence of various IPTG concentrations (100 mM, 10 mM, 1 mM, 100 μM, 10 μM, 1 μM, 100 nM and 0 M), arresting the growth step at the 20-h time-point to the recorded data.

## RESULTS

### Engineering coupled inducible promoters

**Figure 1.**
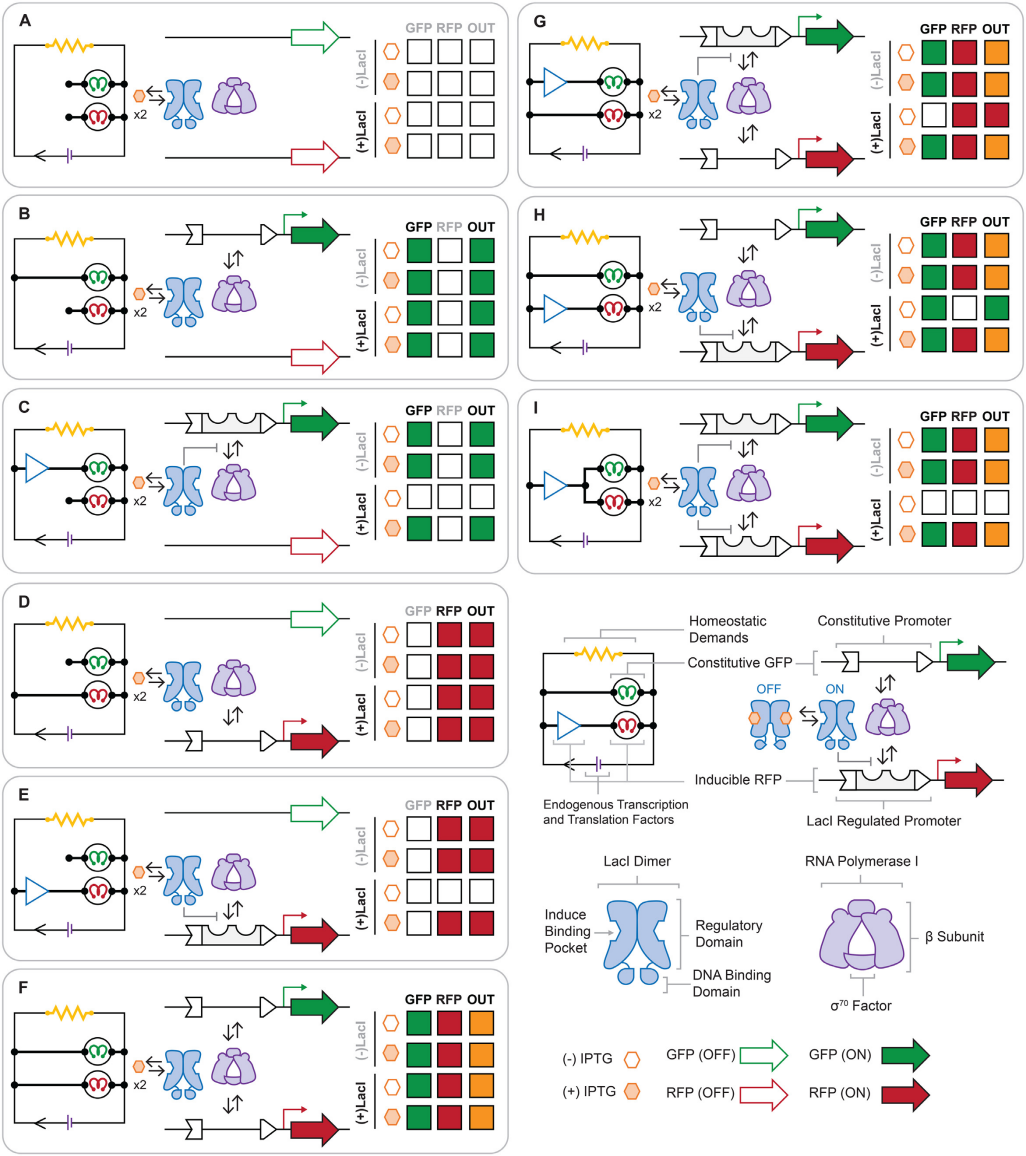
Architecture and anticipated behaviour of engineered signal coupled genetic circuitry constructed in this study. All plasmids constructed in this study contain super-folder green fluorescent protein (GFP) and the red fluorescent protein mCherry (RFP), flanked by identical rrnB T1 terminators, and identical promoter cassettes delivering variable constitutive or inducible promoters. The expression of GFP and RFP reporter proteins is monitored under four experimental conditions: −LacI/−IPTG, −LacI/+IPTG, +LacI/−IPTG, and +LacI/+IPTG. Plasmid architectures constructed in this study include: promoter omitted GFP and RFP (**A**), constitutive expression of GFP (**B**), inducible expression of GFP (**C**), constitutive expression of RFP (**D**), inducible expression of RFP (**E**), constitutive expression of both GFP and RFP (**F**), inducible expression of GFP coupled with constitutive expression of RFP (**G**), constitutive expression of GFP coupled with inducible expression of RFP (**H**) and inducible expression of both GFP and RFP (**I**). A figure legend included in the bottom right panel depicts molecular components and their organization.

To measure the comparative expression profiles from two competing promoters, we developed a collection of two-channel reporter systems (Figure 1, and Supplementary Figure S1). In this schema, each channel was dedicated to the production of either super-folder green fluorescent protein (GFP), or mCherry fluorescent protein (RFP). In Figure 1 we summarized the nine architectures used to progressively construct (and subsequently analyze) coupled promoters used in this study. In brief, each single-channel system is represented in one of three configurations: (i) without a functional promoter (Figure 1A), (ii) with a constitutive-promoter (Figure 1B, D) or (iii) with an inducible-promoter (Figure 1C, E). Here, we define an inducible-promoter as a system composed of the lactose repressor (LacI) transcription factor and a cognate DNA operator. Thus, an inducible-promoter system is in the ON-state when the ligand IPTG is present (i.e. induced—producing a given fluorescent protein) or in the OFF-state without IPTG (i.e. repressed—impeding the production of a given fluorescent protein), see Figure 1C and E. Collectively, the combinatorial space for systems composed of two non-synonymous channels (in a given configuration) is defined by 9 distinct sets (see Figure 1)—of which, only one system can be regarded as potentially signal-coupled (Figure 1I). To confer direct signal-coupling *via* IPTG the architecture illustrated in Figure 1I requires the incorporation of a pair of cognate DNA operators (one per channel) that are modulated by a shared LacI transcription factor. However, we posit that any two-channel system with two functional promoters will experience some degree of RNAP resource partitioning.

**Figure 2.**
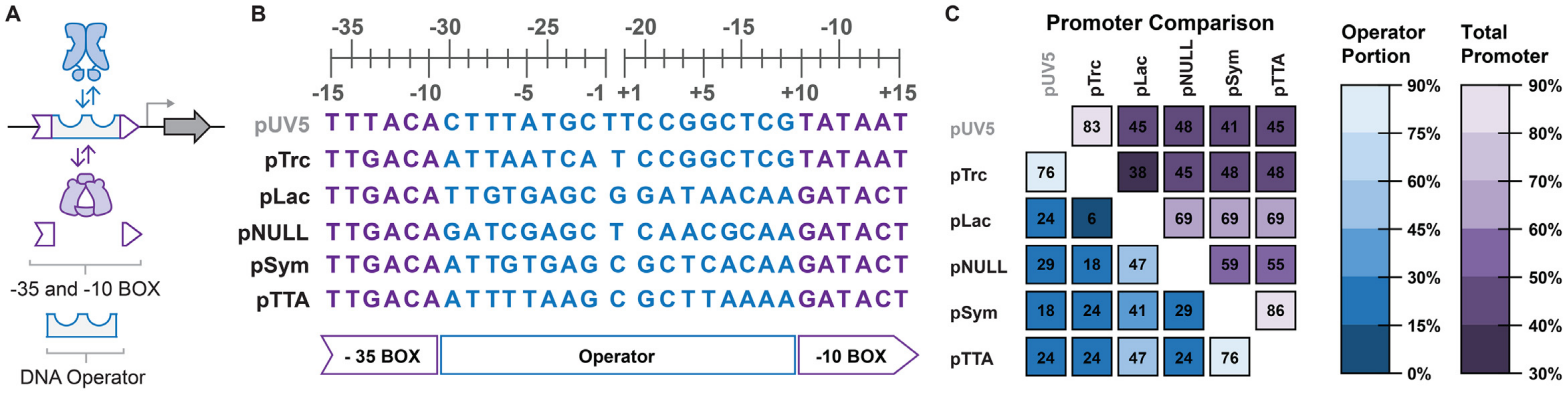
Promoters employed in this study can be constitutive or inducible with operator sequences inserted between the −35 and −10 boxes (**A**). The sequence identity of promoters incorporated into our plasmid architecture (**B**) include the constitutive promoters pUV5, pTrc, and pNull, and the inducible promoters pLac, pSym and pTTA. A sequence alignment and comparison of sequence identity (**C**) was performed to identify promoters of high similarity (pUV5 and pTrc, pSym and pTTA) and low similarity (pNull and pUV5, pNull and pTrc) across the operator (blue) portion or total sequence (purple) of each promoter.

In a given inducible system (Figure 1C, E, G–I), the integrated promoter and operator regions (Figure 2A and Supplementary Figure S1) can be regarded as a single variable unit. Thus, the variable inducible-promoter elements and constitutive-promoter elements deliver the experimental parameter tested in this study. The inducible-promoter elements used in this work leverage a structure in which the LacI DNA operator (i.e. the element directing DNA binding of the transcription factor) is intercalated between the –35 and –10 hexamers—which facilitate RNA polymerase binding and function – such that this configuration has been classified as a core operator position (49) (Figure 2A and Supplementary Figure S1). The choice to vary inducible-promoter elements by the identity of their core sequence ensures that binding of RNAP or LacI repressor are mutually exclusive competitive events, as it has been reported that operator sequences placed distal or proximal to the promoter element can simultaneously accommodate the recruitment of both RNAP and LacI (49,50) (Supplementary Figure S1). Here we have constructed six non-synonymous promoter modules—three constitutive-promoter elements (pNull, pUV5 and pTrc), and three inducible-promoter elements (pLac, pSym and pTTA), Figure 2B, C. Each of the promoter modules were designed to confer different degrees of transcription and transcriptional control. Four modules utilize consensus sequences at the –35 and –10 hexamer elements (i.e. TTGACA and GATACT, respectively)—thus, are anticipated to recruit RNAP for the expression of their reporter genes with the same frequency. However, the region between the hexamer elements is variable between the four modules thus changing the operational scope of their ability to recruit the cognate transcription factor, and potentially alters the affinity of the σ70 factor for the promoter sequence. Finally, the constitutive weak and strong bacterial promoter sequences conferred by pUV5 (44) and pTrc (45) (respectively) introduce putative boundaries to test the limits of resource partitioning. In summary, each genetic construct tested in this study consists of a variable (29–30 bp) promoter element inserted between a 60 bp upstream sequence and 115 bp downstream sequence delivering the genetic insulator riboJ and the ribosome binding site. The sequence architecture of these upstream and downstream elements remain unchanged across all promoter modules employed in this work in an attempt to reduce changes in expression profile output, to changes in promoter element composition.

### Qualitative analysis of coupled promoters

**Figure 3.**
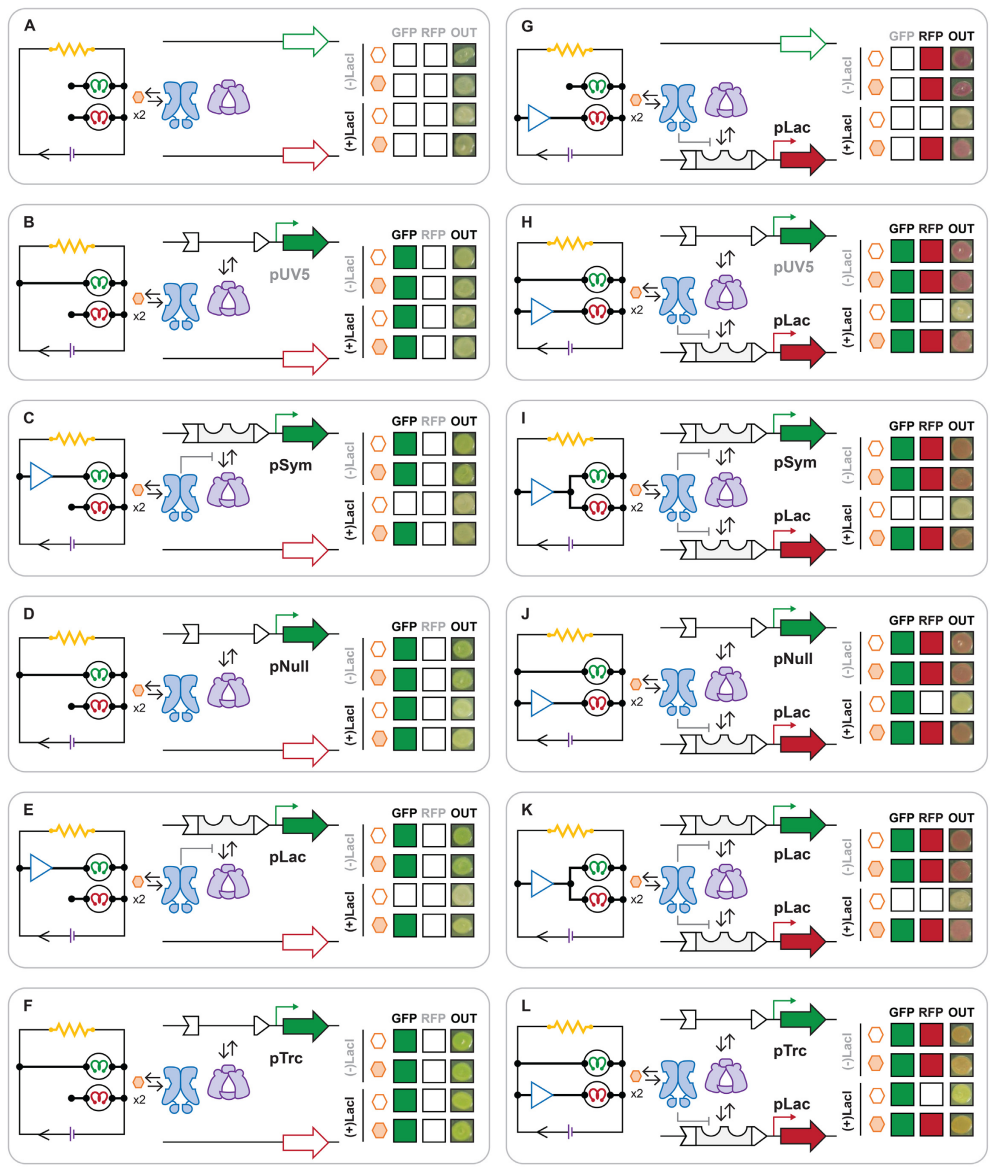
Architecture and observed colony plaque phenotype (OUT) for engineered signal couple plasmids constructed in this study. Plasmid architectures incorporate various constitutive and inducible promoters controlling expression of GFP and RFP reporter proteins, including: promoter omitted GFP and RFP (**A**), constitutive expression of GFP using pUV5 (**B**), inducible expression of GFP using pSym (**C**), constitutive expression of GFP using pNull (**D**), inducible expression of GFP using pLac (**E**), constitutive expression of GFP using pTrc (**F**), inducible expression of RFP using pLac (**G**), constitutive expression of GFP using pUV5 coupled with inducible expression of RFP using pLac (**H**), inducible expression of GFP using pSym and RFP using pLac (**I**), constitutive expression of GFP using pNull coupled with inducible expression of RFP using pLac (**J**), inducible expression of both GFP and RFP using pLac (**K**), constitutive expression of GFP using pTrc coupled with inducible expression of RFP using pLac (**L**).

In a preliminary assessment of promoter coupling, we evaluated five dual-promoter systems *via* a qualitative solid media screen, Figure 3. In this experiment, we assayed promoter modules pUV5, pSym, pNull, pLac and pTrc *via* a green-channel, relative to a red-channel adapted with a fixed inducible pLac element. The qualitative phenotypes presented in Figure 3 depict the relative expression of GFP (green) and RFP (red) of colony plaques following co-transformation, with (+LacI) and without (–LacI) transcriptional regulation. All systems were incubated for 24-h at 37°C on Luria-Bertani agar – with ligand (+IPTG) and without ligand (−IPTG). Single-channel data were collected for reporter plasmids grouped according to whether the system contained no promoter elements on the opposing channel, designated as GFP minus (−pGFP) or RFP minus (−pRFP), such that: (i) a single constitutive-promoter element (pUV5, pNull or pTrc) initiated the transcription of GFP alone (GFP | −pRFP), Figure 3B, D, F; (ii) a single inducible-promoter element (pSym, or pLac) regulated the transcription of GFP alone (GFP | −pRFP), Figure 3C,E; (iii) finally, a single system regulated RFP output (RFP | −pGFP) *via* the inducible-promoter pLac, Figure 3G. The reporter plasmids that expressed GFP under single constitutive-promoter elements showed a distinct difference in the presentation of plaque phenotypes for pUV5 (Figure 3B) and pTrc (Figure 3F), and gave the weakest and strongest green colours, respectively. Notably, the single red-channel system (Figure 3G) clearly illustrated induction. However, a qualitative ranking of the remaining single-promoter green-channel systems could not be accomplished as a result of inherent difficulties distinguishing GFP output alone *via* visual inspection within the observed colony plaques.

In turn, qualitative two-channel data were collected as dual-promoter elements with variation in the initiation of GFP *via* pUV5, pSym, pNull, pLac or pTrc, with a fixed inducible pLac promoter for RFP (Figure 3H-L). The schema for a variable constitutive green-channel (pUV5, pNull or pTrc) paired with a fixed inducible red-channel (pLac) are given in Figure 3H, J, L. Whereas, the schema for dual-inducible channels are given in Figure 3I, K, such that the systems pair a variable green-channel regulated by pLac or pSym with a fixed red-channel regulated by pLac. The change in plaque colour phenotypes range from red to orange, implying the concomitant expression of RFP with GFP promoter elements of variable strength. Pairing functional lac repressor (+LacI) with reporter plasmids delivering the inducible pLac RFP promoter element in conjunction with the constitutive pNull or pTrc GFP promoter elements presented a distinct change of phenotype from green in the absence of inducer to red/orange in the presence of inducer. The distinction between red-channel systems is more apparent – relative to the green-channel alone – implying some degree of promoter coupling. However, a more quantitative assessment of systems (in which red and green channels can be assessed separately) is necessary to better articulate the extent of resource partitioning.

### Quantitative analysis of coupled promoters

**Figure 4.**
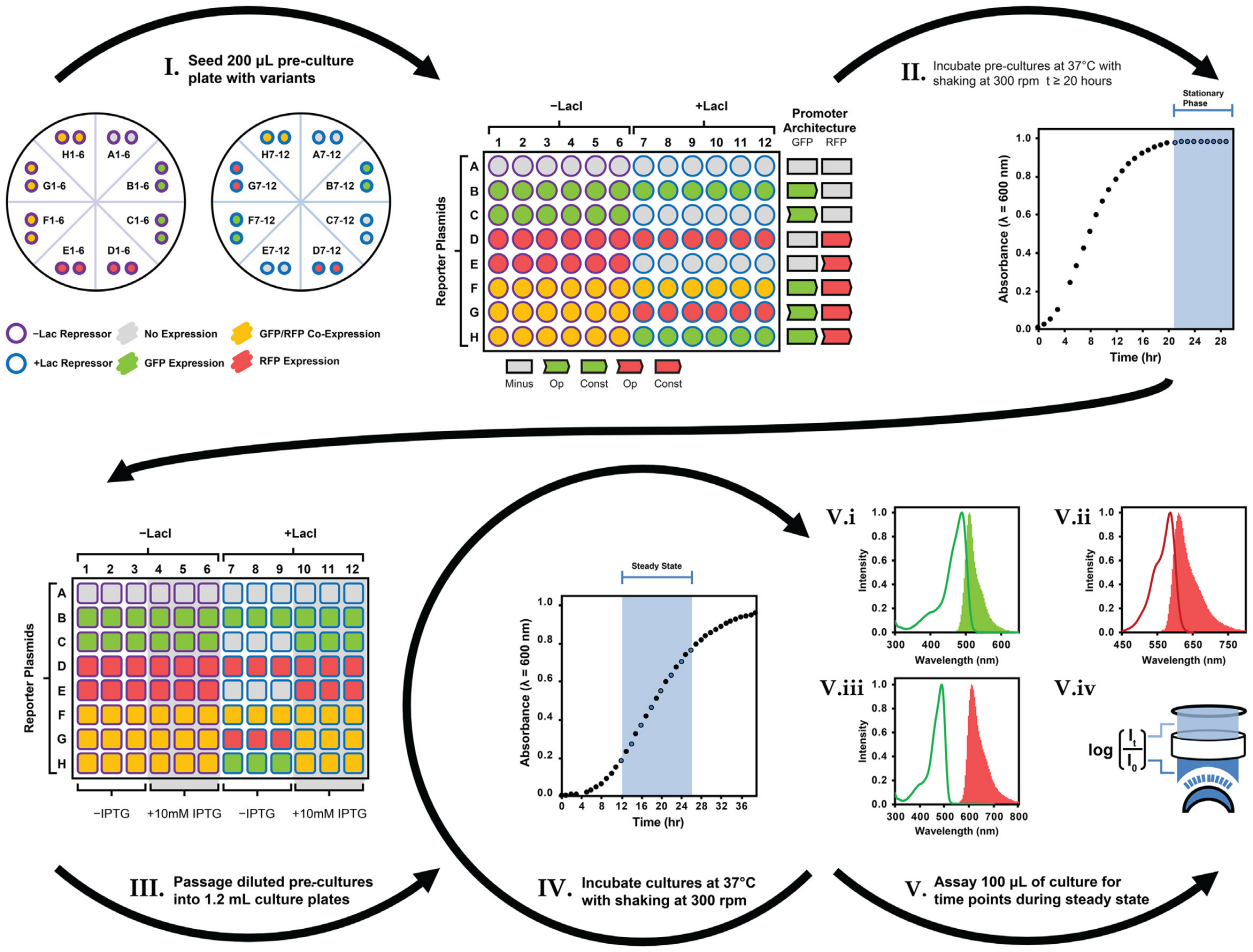
Expression assay protocol for quantification of signal coupled reporter plasmid variants. I: Minimal media pre-cultures (200 μl) are seeded with co-transformed reporter plasmids (non-, constitutive- and inducible- expressing) and repressor plasmids conferring the control (−LacI) and wild-type LacI repressor (+LacI). II: Pre-cultures are grown to stationary phase (*t* = 24 h) by incubation (37°C) with shaking (300 rpm) for 24 h. III: Pre-cultures are diluted (OD_600 nm_ = 0.01) and passaged into minimal media cultures (1.2 ml) and grown for 12 h by incubation (37°C) with shaking (300 rpm). IV: 100 μl of each culture is sampled in 2 h increments during steady state (*t* = 12, 14, 16, 18, 20, 22, 24 and 26 h). V: Samples are assayed to determine signal output resulting from GFP and RFP expression. Specifically, V.i: green fluorescence (λ_ex_ = 485 nm, λ_em_ = 510 nm, gain = 400), V.ii: red fluorescence (λ_ex_ = 585 nm, λ_em_ = 610 nm, gain = 400), V.iii: green-to-red fluorescence by forester resonance energy transfer (λ_ex_ = 485 nm, λ_em_ = 610 nm, gain = 400) and V.iv: culture density (λ_abs_ = 600 nm), are quantified.

To quantitatively assess single-channel and dual-channel promoter systems, a microplate format culture assay was implemented (Figure 4). The assay is conducted over a course of two days, involving the co-transformation of the repressor and cognate reporter systems, their inoculation and growth in minimal media (−IPTG, 200 μl, 37°C, 300 rpm, 20 h) to stationary phase, culture dilution (OD_600_ = 0.01) and passage into fresh minimal media (with and without IPTG, 1.2 ml, 37°C, 300 rpm) for growth, and the sampling of 100 μl culture volumes between 12 and 26 h in 2 h increments. Culture samples were then assayed to determine density (λ_abs_ = 600 nm), GFP expression (λ_ex_ = 485 nm, λ_em_ = 510 nm), and RFP expression (λ_ex_ = 585 nm, λ_em_ = 610 nm). The assay data for each reporter system and experimental condition—i.e. with and without LacI and cognate inducer—was extracted and analyzed to determine changes to the expression of GFP and RFP. Specifically, plots of absorbance (ABS) versus time, GFP versus ABS, RFP versus ABS, and Förster resonance energy transfer (FRET) versus ABS (control), were compiled to identify and fit the linear region for each expression metric and extract their value (Supplementary Figure S2 and Supplementary Tables S1 and S2). The FRET control experiment confirmed a lack of undesirable protein-protein interactions between GFP and RFP. Accordingly, study of expression plots for the set of reporter systems enabled quantitative assignment of phenotypes and degree of promoter coupling.

**Figure 5.**
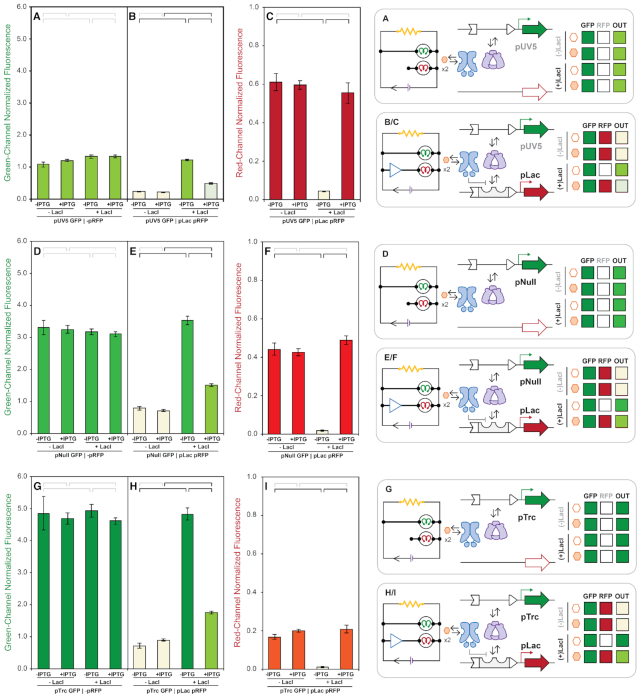
Signal coupled reporter plasmid expression of constitutive GFP promoters paired with absent or inducible RFP promoter across assay time-points at steady-state. GFP expression using pUV5 in the absence (**A**) or presence (**B**) of inducible RFP expression using pLac. RFP expression using pLac in the presence of GFP expression using pUV5 (**C**). GFP expression using pNull in the absence (**D**) or presence (**E**) of inducible RFP expression using pLac. RFP expression using pLac in the presence of GFP expression using pNull (**F**). GFP expression using pTrc in the absence (**G**) or presence (**H**) of inducible RFP expression using pLac. RFP expression using pLac in the presence of GFP expression using pTrc (**I**). Fluorescence from green (λ_ex_ = 485 nm, λ_em_ = 510 nm, gain = 400) and red (λ_ex_ = 585 nm, λ_em_ = 610 nm, gain = 400) channels normalized by culture density (λ_abs_ = 600 nm), and reported in ×10^4^ relative fluorescence units. Brackets comparing signal output between experimental conditions (−LacI/−IPTG, −LacI/+IPTG, +LacI/−IPTG and +LacI/+IPTG) indicate homoscedastic two-tailed t-test statistic results for no significant difference (gray, *P* ≥ 10^−2^) and significant difference (black *P* < 10^−2^).

Given the lack of apparent distinction *via* the solid media screen on the green-channel, our first objective using our in solution assay was to quantify the performance and distinguish putative constitutive-promoters pUV5, pNull, and PTrc (Figure 5). In Figure 5A, D, G the data clearly illustrated that all three promoters can be regarded as constitutive – as none of the systems are regulated by the LacI transcription factor. In addition, we can now rank the relative constitutive-promoter strengths as follows pTrc > pNull > pUV5. In turn, we evaluated the impact of RNAP resource partitioning on dual-promoter systems, in which the red-channel is regulated by the inducible-promoter pLac (Figure 5B, E, H). In Figure 5B, we paired the constitutive-promoter pUV5 (green) with the inducible pLac promoter (red), and evaluated the relative performance of the green-channel upon regulation of the red-channel. In the repressed-state the red-channel was OFF (Figure 5C), and the complementary green-channel was ON (Figure 5B)—producing GFP at the same level as the single-channel system, as expected. In the induced-state the red-channel was ON; however, the green-channel had diminished production of GFP (Figure 5B). We posited that the observed reduction of GFP output was the result of RNAP depletion *via* activation of the red-channel upon induction. Accordingly, we surmised that a similar effect would be observed with dual-promoter systems composed of pNull or pTrc initiating the green-channel (Figure 5E, H). Moreover, the influence of increasing the constitutive-promoter strength of the green-channel can be observed (quantified) on the opposing red-channel post-induction (Figure 5C, F, I). Namely, as the constitutive-promoter (green-channel) strength increased the dynamic range of the red-channel decreased—illustrating RNAP quantity limits at steady-state, resulting in an observable resource partitioning.

**Figure 6.**
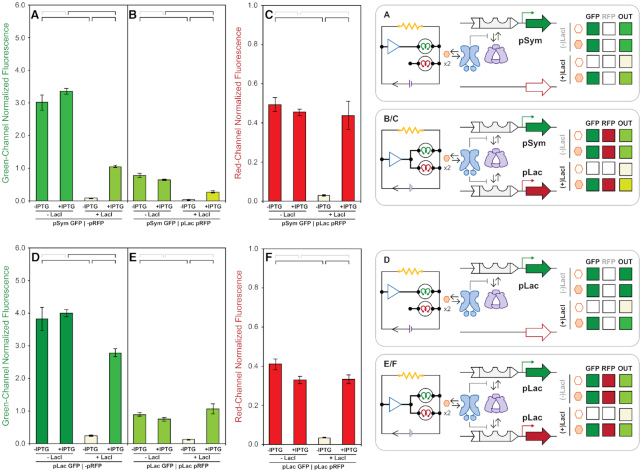
Signal coupled reporter plasmid expression of inducible GFP promoters paired with absent or inducible RFP promoter across assay time-points at steady-state. GFP expression using pSym in the absence (**A**) or presence (**B**) of inducible RFP expression using pLac. RFP expression using pLac in the presence of GFP expression using pSym (**C**). GFP expression using pLac in the absence (**D**) or presence (**E**) of inducible RFP expression using pLac. RFP expression using pLac in the presence of GFP expression using pLac (**F**). Fluorescence from green (λ_ex_ = 485 nm, λ_em_ = 510 nm, gain = 400) and red (λ_ex_ = 585 nm, λ_em_ = 610 nm, gain = 400) channels normalized by culture density (λ_abs_ = 600 nm), and reported in ×10^4^ relative fluorescence units. Brackets comparing signal output between experimental conditions (−LacI/−IPTG, −LacI/+IPTG, +LacI/−IPTG, and +LacI/+IPTG) indicate homoscedastic two-tailed t-test statistic results for no significant difference (gray, *P* ≥ 10^−2^) and significant difference (black *P* < 10^−2^).

Next, we evaluated bi-lateral inducible-promoters, in which the system regulated the green-channel pSym or pLac (Figure 6). First, we monitored the green-channel alone *via* either inducible-promoter (Figure 6A,D). We observed the expected phenotype for pSym and pLac—however, the difference between the repressed and induced state was larger for the pLac system. Next, we paired each of the green-channels with a pLac inducible red-channel (Figure 6C, F). We observed LacI mediated regulation of the red-channel (Figure 6C, F), and complementary (signal-coupled) regulation of the non-synonymous green-channels (Figure 6B, E). Taken together the bi-lateral inducible systems harbored the expected phenotypic response on both channels—in contrast to the dual-promoter systems that employed constitutive green-channels. However, the coupled inducible systems displayed some degree of RNAP resource partitioning as the dynamic range (of the induced-states, and repressed-states to a lesser extent) on the green-channels were diminished relative to the inducible single-channel systems.

### Correlation between promoter strengths and reporter outputs

**Figure 7.**
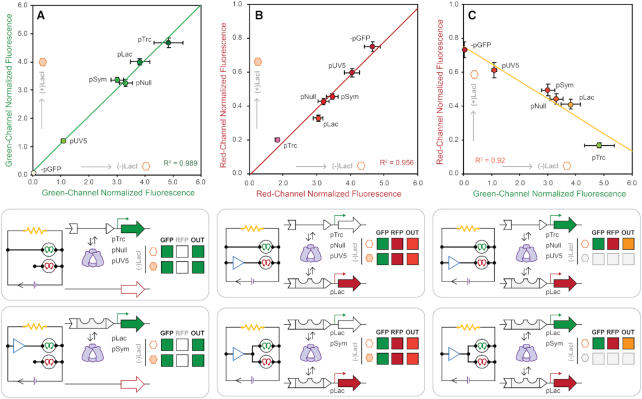
Comparison of density normalized GFP and RFP expression across the set of signal coupled and uncoupled reporter plasmids. GFP expression uncoupled from RFP expression (−pRFP) at steady state for reporter plasmids containing the −pGFP, pUV5, pSym, pNull, pLac and pTrc promoters in the absence (x-axis) and presence (y-axis) of 10 mM IPTG (**A**). RFP expression under pLac uncoupled and coupled with GFP expression under pUV5, pSym, pNull, pLac, and pTrc promoters in the absence (x-axis) and presence (y-axis) of 10 mM IPTG (**B**). Comparison of coupled RFP expression (y-axis, −IPTG) as a function of uncoupled GFP expression (x-axis) at steady-state, for reporter plasmids containing the +pLac RFP and –pGFP, pUV5, pSym, pNull, pLac and pTrc GFP promoter cassettes (**C**). Fluorescence from green (λ_ex_ = 485 nm, λ_em_ = 510 nm, gain = 400) and red (λ_ex_ = 585 nm, λ_em_ = 610 nm, gain = 400) channels normalized by culture density (λ_abs_ = 600 nm), and reported in ×10^4^ relative fluorescence units.

In addition to facilitating the assessment of promoter coupling, our quantitative microwell assay was used to rank order the promoter strength of the five promoters originally assessed (and unsuccessfully ranked) in our qualitative solid media screen (Figure 7A). Promoter performances were measured in the absence of functional LacI (i.e. unregulated), with and without the inducer IPTG enabling the relative comparison of promoter strength alone. The linear correlation of this comparison (*R*^2^ = 0.989) resulted improved resolution of the assignment of promoter strength. In agreement with the qualitative colony plaque assessment (Figure 3B, F) pUV5 and pTrc represented the weakest and strongest promoters (relatively) in the quantitative assay (Figure 7A). The absolute ranking of all five promoter strengths was observed as follows pUV5 < pSym ≈ pNull < pLac < pTrc. Likewise, measurement of RFP expression output under increasing GFP promoter burden showed a high degree of correlation (*R*^2^ = 0.956) between RFP output relative to reported GFP promoter strength – though inverted (Figure 7B). This result strongly suggests that there exists a linearly correlated, binary partitioning of endogenous transcription and translation resources. This competition can be attenuated by changing the relative strength of competing promoter element (Figure 7C) – reconciling the observations given in Figures 5 and 6. For example, the pLac RFP promoter element produced a stronger red channel output when paired with the weak pUV5 GFP promoter, compared to red-channel output when paired with the strong pTrc promoter driving the green-channel (Figure 7B, C).

### Dose-response between competing promoters

**Figure 8.**
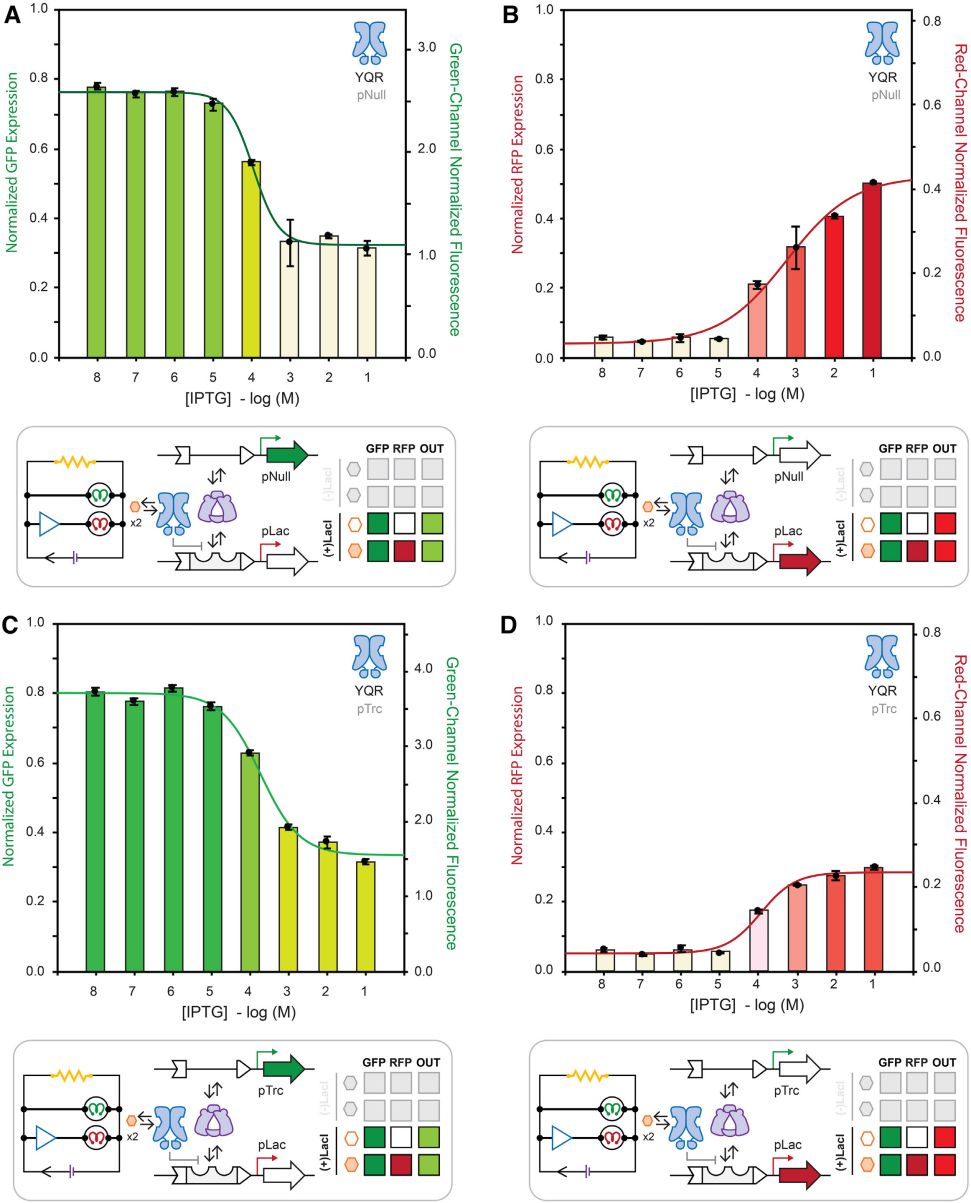
Inducer titration isotherms comparing constitutive GFP and inducible RFP coupled-expression at steady-state (*t* = 20 h) under wild-type LacI regulation. GFP (**A**) and RFP (**B**) expression for a reported plasmid delivering the promoters pNull GFP and pLac RFP. GFP (**C**) and RFP (**D**) expression for a reported plasmid delivering the promoters pTrc GFP and pLac RFP. Fluorescence from green (λ_ex_ = 485 nm, λ_em_ = 510 nm, gain = 400) and red (λ_ex_ = 585 nm, λ_em_ = 610 nm, gain = 400) channels normalized by culture density (λ_abs_ = 600 nm), and reported as the fraction of total potential fluorescence as well as ×10^4^ relative fluorescence units, on left and right vertical axes, respectively.

**Figure 9.**
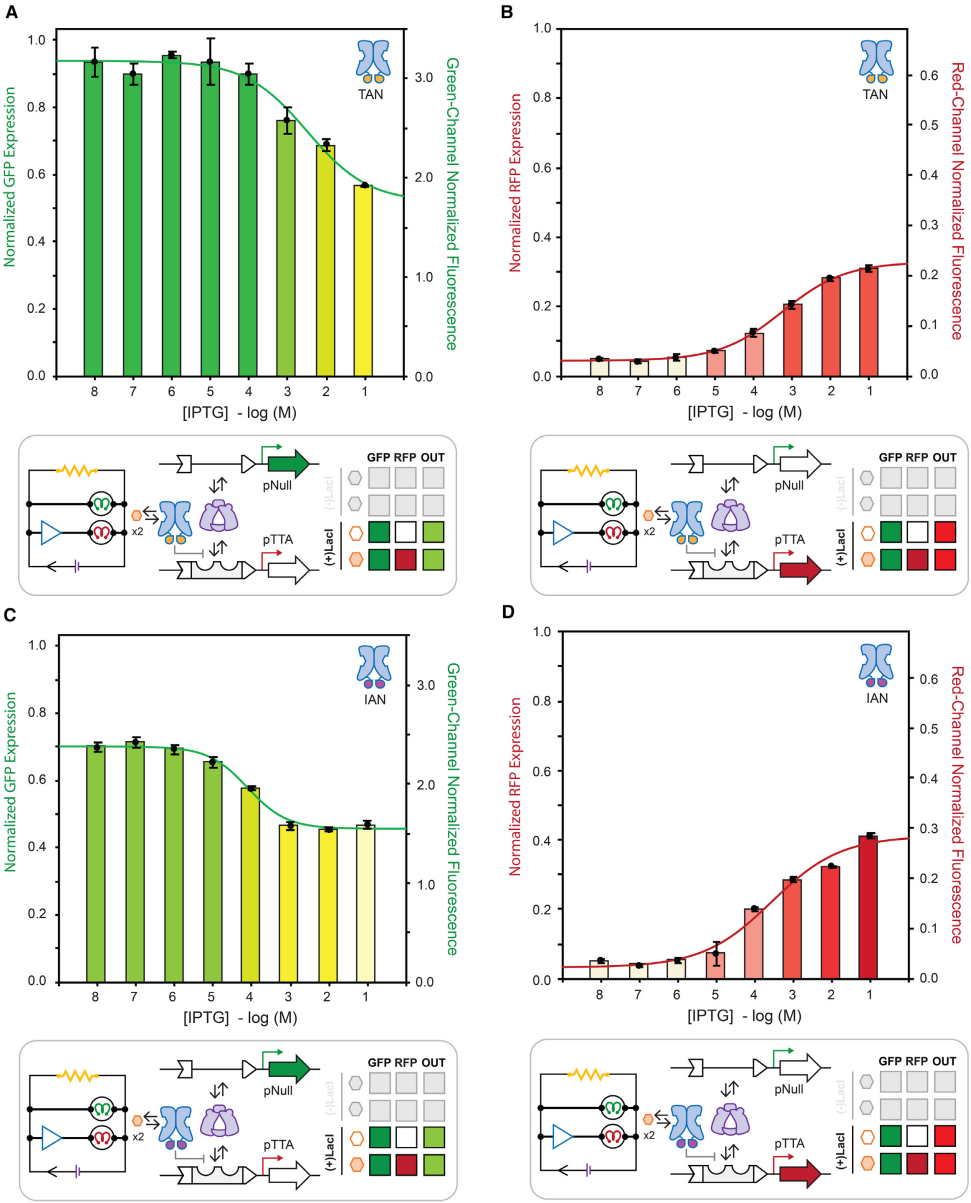
Inducer titration isotherms comparing coupled constitutive GFP expression using pNull and inducible RFP expression using pTTA at steady-state (t = 20 hours) under alternate DNA binding domain LacI regulation. GFP (**A**) and RFP (**B**) expression regulated using LacI variant Y17T/Q18A/R22N (TAN). GFP (**C**) and RFP (**D**) expression regulated using LacI variant Y17I/Q18A/R22N (IAN). Fluorescence from green (λ_ex_ = 485 nm, λ_em_ = 510 nm, gain = 400) and red (λ_ex_ = 585 nm, λ_em_ = 610 nm, gain = 400) channels normalized by culture density (λabs = 600 nm), and reported as the fraction of total potential fluorescence as well as × 10^4^ relative fluorescence units, on left and right vertical axes, respectively.

Having demonstrated an apparent RNA-polymerase resource competition between dual-promoters, we next sought to determine the properties of resource competition in response to varying concentrations of IPTG (Figures 8 and 9). To facilitate our investigation, eight dual-promoter systems (4-sets) with functional LacI repressor variants were constructed. Generically, the two-channel reporter systems were composed of mixed promoter elements, in which a constitutive-promoter drives expression of the green-channel and an inducible-promoter regulates expression *via* the red-channel. Having previously determined that all assayed reporter systems collected beyond the 14-hour time-point present linear changes in fluorescent output with respect to changes in culture density, the experiment was setup to monitor includible expression changes at the 20-h time-point under variable concentrations of the cognate inducer IPTG. All dose-response curves were fitted to a sigmoid curve describing the coupled system's relative leakiness (E_0_), dynamic range (D), effective concentration of inducer at 50% output (EC_50_), and transition slope (*k*) (Supplementary Figure S3 and Supplementary Tables S3–S5).

In our first case study, we selected the intermediate mixed promoter system evaluated in Figure 5E, F—in which a constitutive pNull green-channel was paired with an inducible pLac red-channel. In the corresponding dose-response experiment (Figure 8A, B), we observed a progressive decrease in green-channel fluorescence output with a compensatory increase in red-channel output as the concentration of IPTG increased. This observation supports our previous supposition that diminished green-channel output for this system was the result of RNA-polymerase resource partitioning—as the progressive increase in IPTG concentration mitigated occupancy of the –35 and –10 hexamers progressively depleting the finite (steady-state) RNAP resource. Accordingly, we posited that increasing the constitutive-promoter strength on the green-channel would diminish the dynamic range of the red-channel—evidenced in Figure 8C, D. Moreover, the increase in promoter strength on the green-channel (from pNull to pTrc) shifts the EC_50_ of the red-channel isotherm to a lower effective IPTG concentration (i.e. tighter apparent LacI binding) as result of reduced competitive binding due to a reduction in free RNAP. This interpretation was further supported in the context of the control experiment in which the green-channel was completely muted, which provided the maximum degree of competition and most right shifted EC_50_ (Supplementary Figure S3A).

### Dose-response and transcription factor variation *via* an orthogonal promoter (pTTA)

To further investigate the apparent behaviour of repressor induction in response to varied competing promoter elements, we converted the pSym operator imbedded in the core of our RFP bacterial promoter to an alternative operator sequence O^tta^ by mutating the core operator bases G6T/T5/G4A to yield the alternate bacterial core promoter **pTTA** (Figure 2B). The wild-type Lac operator (O^1^ | pLac) and the symmetric variant (O^sym^ | pSym) associate with the wild-type DNA binding domain sequence of LacI Y17/Q18/R22. Mutation of the DNA binding domain to Y17**T**/Q18**A**/R22**N** (TAN) results in a LacI variant that is incapable of repressing transcription regulated with the O^1^ or symmetric O^sym^ operator elements. Instead, the LacI-TAN variant associates with the O^tta^ (pTTA) operator sequence. Moreover, construction of the **pTTA** promoter provides an additional avenue with which we can investigate the influence of promoter burden on the apparent behaviour of a given repressor variant (Figure 9). Previous studies demonstrated that select mutations to the LacI DNA binding domain alter the LacI transcription factor's affinity for the O^tta^ operator element. Namely, repressor DNA binding domain variants TAN, IAN, and VAN can interact with the O^tta^ operator element – however with different expression profiles (48). This feature allowed us to change the experimental variable from the promoter element to the transcription factor. We posited that the variation in expression profiles conferred by the relative strength of the protein (transcription factor) DNA interaction will tune the performance of the opposing channel—such that the stronger the interaction on the inducible channel the greater the displacement of RNA-polymerase.

At the outset we used the absolute dynamic range and degree of leakiness of single-channel systems as proxies to estimate the relative strength of the protein (transcription factor) DNA interaction for the TAN, IAN and VAN variants (Supplementary Figure S3 and Supplementary Tables S3–S5). These data suggest that relative dynamic interaction strength was approximately TAN > IAN > VAN using a fixed O^tta^ (pTTA) promoter-operator element under the conditions tested. In turn, we constructed a mixed promoter system in which a constitutive pNull green-channel was paired with an inducible pTTA red-channel. Next we paired the pNull | pTTA duel reporter system with LacI variants TAN, IAN or VAN (Figure 9, and Supplementary Figure S3). Consistent with our working hypothesis the TAN system displayed the greatest amount of RNAP displacement followed by IAN, and finally VAN – as observed by the couple performance of the green-channel. Interestingly, increasing the promoter strength on the green-channel (i.e. from pNull to pTrc) had the most significant effect on the VAN and IAN red-channels respectively – and the smallest observed differences for the TAN system. This observation suggests that the pTTA | LacI-TAN dual-promoter system is approximately resource balanced between the pNull and pTrc promoter strengths.

### Global assessment of dual-promoter systems

**Figure 10.**
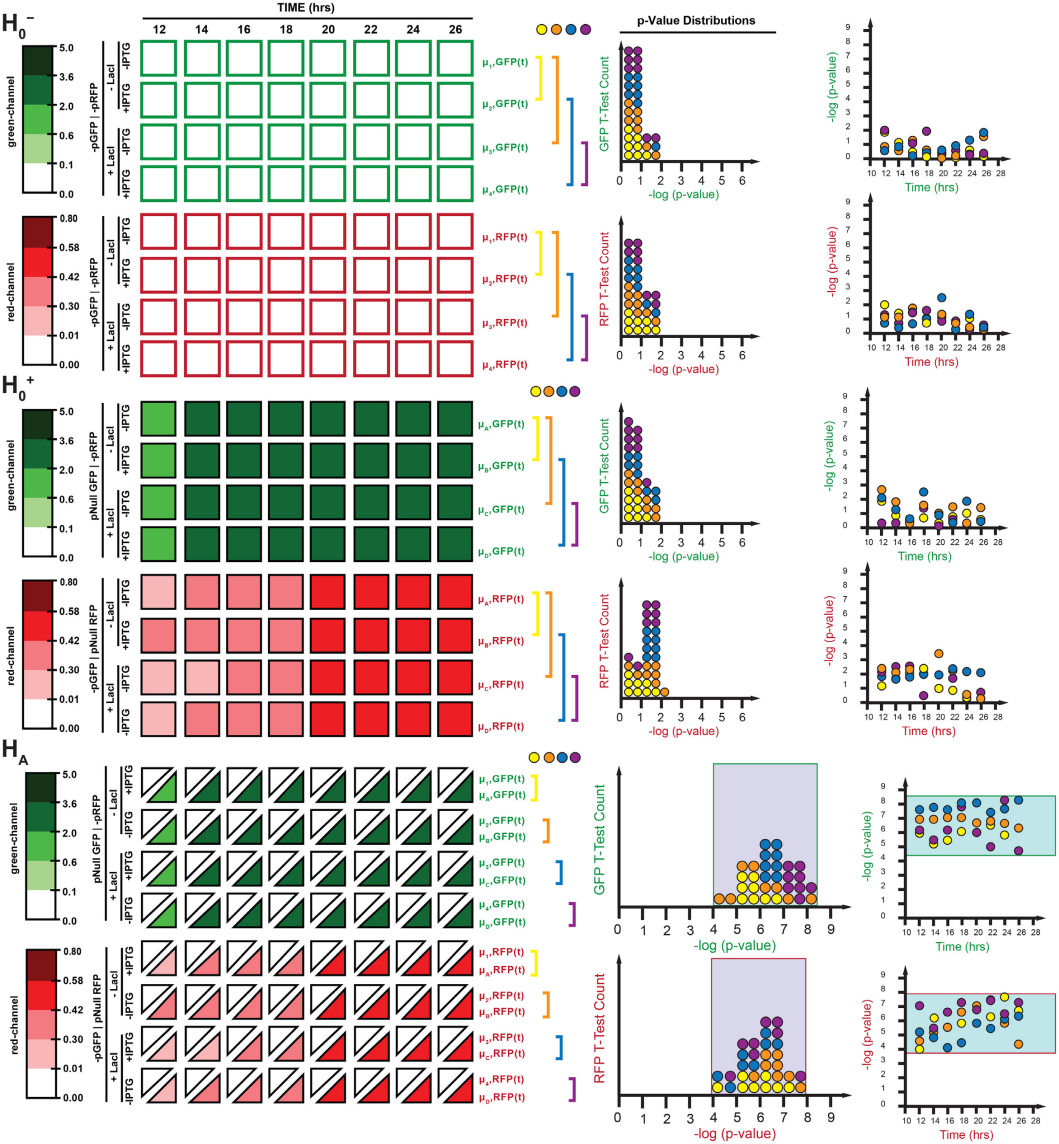
Comparison of GFP and RFP expression by p-value statistics from t-tests (two-tailed, homoscedastic, *n* = 3) over a time course (12, 14, 16, 18, 20, 22, 24, 26 h) between two populations of cultures varying by experimental conditions (−LacI/−IPTG, −LacI/+IPTG, +LacI/−IPTG and +LacI/+IPTG). Negative controls (H_0_) include comparisons between populations incapable of expressing reporter protein (H_0_^−^, −pGFP / −pRFP) or populations that include the signal uncoupled constitutive expression of GFP or RFP using the pNull promoter (H_0_^+^, pNull GFP / −pRFP or −GFP / pNull RFP). Positive control (H_A_) includes comparisons of GFP and RFP expression between populations of reporter plasmids with promoter absent (H_0_^−^) and constitutive pNull promoters (H_0_^+^). Histograms and scatter plots indicated the frequency and time-point of each p-value comparison, respectively. Thresholds for experimentally determined statistically significant results are indicated by the green and red outlined boxes for GFP and RFP expression comparisons, respectively. Fluorescence from green (λ_ex_ = 485 nm, λ_em_ = 510 nm, gain = 400) and red (λ_ex_ = 585 nm, λ_em_ = 610 nm, gain = 400) channels normalized by culture density (λ_abs_ = 600 nm), and reported in × 10^4^ relative fluorescence units.

**Figure 11.**
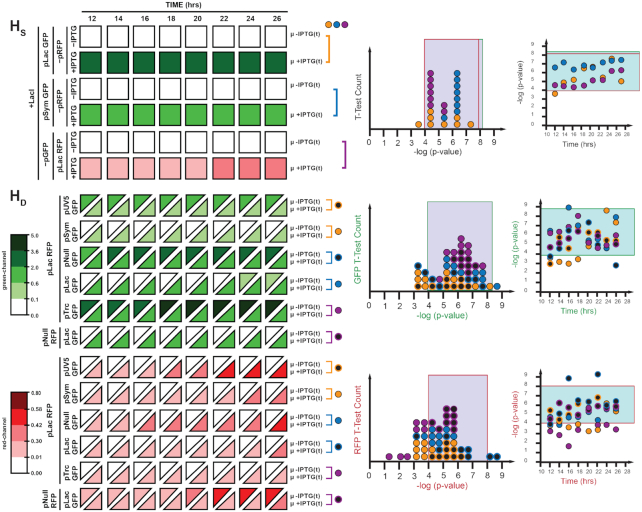
Comparison of GFP and RFP expression by p-value statistics from t-tests (two-tailed, homoscedastic, *n* = 3) over a time course (12, 14, 16, 18, 20, 22, 24, 26 h) between two populations of cultures varying by experimental conditions (+LacI/−IPTG and +LacI/+IPTG). Decoupled single promoter expression (H_S_) is measured for GFP expression under pSym or pLac and measured for RFP expression under pLac. Coupled dual promoter expression (H_D_) is measured for GFP expression using pUV5, pSym, pNull, pLac or pTrc and RFP expression using pLac. Histograms and scatter plots indicated the frequency and time-point of each p-value comparison, respectively. Thresholds for experimentally determined statistically significant results derived from the positive control in Figure 10 are indicated by the green and red outlined boxes for GFP and RFP expression comparisons, respectively. Fluorescence from green (λ_ex_ = 485 nm, λ_em_ = 510 nm, gain = 400) and red (λ_ex_ = 585 nm, λ_em_ = 610 nm, gain = 400) channels normalized by culture density (λ_abs_ = 600 nm), and reported in ×10^4^ relative fluorescence units.

The ability to distinguish between reporter output signals when repressed and induced is a defining prerequisite for functionally useful synthetic biological systems. The successful interrogation and correct interpretation of any genetic sensor or genetic circuit's output requires that there is a distinct and easily defined threshold of signal between reporter outputs when OFF and ON. It is unclear how competition between co-expressed promoter elements would influence our ability to distinguish between repressed and induced states of our reporters. With this objective, we sought to measure changes between induced and repressed pLac (or pTTA) RFP expression in the absence and presence of competing GFP promoter cassettes (Figures 5–9). To facilitate a more global comparison, we objectively bin these data *via* the k-means machine learning algorithm (51) as applied to the GFP and RFP normalized expression. Briefly, the k-means algorithm functions by partitioning data points according to their value into a predefined number of groups. Each group has a centroid, calculated as the average value across all data points that belong to the group. The algorithm then iteratively re-sorts all data points according to their distance from each centroid. Convergence of the algorithm was achieved when sorting results in centroids that cease to change from the previous iteration, thus minimizing the difference between each data point and its group's centroid. Reporter expression outputs were clustered into five groups of increasing magnitude, having the lowest root-mean-square deviation (RMSD) when compared with all other possible centroid combinations. The results are summarized as scale bars in Figures 10 and 11.

In turn, we defined a collection of three controls with which to interpret our distributions (Figure 10), where: (i) H_0_^−^ defines the difference in output signal in the absence and presence of inducer for the reporter genes for GFP or RFP co-expressed with and without LacI, minus promoter elements on the green and red channels, (ii) H_0_^+^ defines the difference in output for discrete single channels under constitutive control, in the absence and presence of inducer with and without LacI, and (iii) H_A_ defines the difference between H_0_^−^ and H_0_^+^ under the same conditions. The H_0_^−^ and H_0_^+^ null controls were expected to provide a lower limit on the signal difference when distinguishing between the output of two populations which should remain unchanged, while the H_A_ alternative control provides an upper limit on the expected change in signal difference between the output of two populations in the absence and presence of a promoter (Figure 10). We justified using the pNull promoter to define the upper and lower performance boundary based on the relative intermediate promoter strength typically used in synthetic biology experiments.

In our first experiment, we evaluated single-channel systems with inducible-promoters (H_S_), relative to the boundaries established *via* H_0_^−^ and H_0_^+^ null controls (Figure 11). T-test counts showed that approximately 96% of the relative comparisons fell within the performance boundary set by the null controls. The one outlier occurred at the earliest time interval tested in the putative steady-state window, accounting for the slight deviation. Next, we sought to conduct and assessment of sets of dual-reporter systems to demonstrate the impact of resource partitioning (Figure 11, H_D_). Monitoring the green-channel 83% of the relative compared states (*i.e*., with and without IPTG) fell within the performance boundary. Approximately 60% of the lower-boundary outliers utilized pSym on the green-channel (versus pLac on the red-channel). Interestingly, approximately 30% of the lower-boundary outliers involved pNull. In the majority of cases, the lower-boundary outliers fell within the first-third of the time-course, likely the result of sampling the system prior to accomplishing steady-state. Only one value fell beyond the upper-boundary (pLac RFP | pLac GFP) and was represented in approximately the first-third of the steady-state time-course. When monitoring the red-channel over the same time course for the same dual reporter systems, the influence of the promoter strength on the opposing channel became apparent. Namely when comparing constitutive promoter values, all of the pTrc systems fell below the lower-boundary, whereas all of the pUV5 systems fell within the putative boundary – and pNull values fell between the two for the most part. However, there was no apparent correlation with the time-course. Interestingly, nearly 60% of the systems with dual-regulated promoters (i.e. pLac and pSym) fell outside of the putative boundary. In addition, when comparing dual-promoter systems in which the promoter element is the same (i.e. pLac GFP | pLac RFP) one can glean the differences that occur due to variation in output and accumulation. While the green-channel fell within the performance boundary nearly 88% of the time, the red-channel only presented within the performance boundary 63% of the time. Given that both channels are essentially genetically identical and mRNA has been normalized by the addition of an insulator (riboJ), the observed differences can be accounted for by the differences in protein output. Nevertheless, the vast majority of these data (∼98%) are clearly distinguishable from the lower-limit boundaries set by the H_0_^−^ and H_0_^+^ null controls – regardless of channel. Accordingly, this global framework has the potential to better predict the behavior of complex genetic circuits that will include the effects of resource partitioning and related phenomenon.

## DISCUSSION

With ever-increasing complexity in genetic circuit design, the need for tools that accurately describe and anticipate effects of resource partitioning are becoming increasingly important. However, the impact of changing the promoter strength on complementary parts within a genetic circuit typically are not considered. This study highlights the importance of the impact of promoter coupling on the resource budget. Moreover, this work illustrates how the position and type of operator element (in addition to output characteristics) can significantly influence the operational scope of programmed gene expression. Our study provides clear evidence for competition of transcription resource allocation between pairs of bacterial promoter constructs. While this competition does not prevent the correct assignment of the repressed and induced status of regulated gene constructs, the phenomenon does prevent the attainment of consistent expression levels in the presence of variable competing promoter elements. A solution to this problem has been proposed by Segall-Shapiro *et al.* with their construction of a transcription resource allocator (32). Their transcription resource allocator is assembled using the orthogonal T7 RNAP, alleviating burden on endogenous bacterial RNAP. The function of multiple T7 RNAP constructs is directed by controlling the expression of fragmented components conferring assembly of specific functional units. These fragmented components direct the specificity, catalytic activity and activation of the fully assembled RNAP unit. Control over the variable expression of these components allows for tuning and normalization of promoter outputs independent of endogenous bacterial RNAP.

While the system tested by Segall-Shapiro *et al.* provides a promising solution for small multiple gene output genetic programs, our results suggest that increasingly complicated resource allocator systems will remain susceptible to resource competition because the components of the allocator rely on the recruitment of endogenous RNAP. Ultimately, we anticipate that there is a theoretical limit on coupled expression output (chemical potential) of a single cell (52). A key experimental consideration for our study required that we could design promoter constructs that prevented simultaneous association of RNAP and repressor elements to our expression cassettes. The study of promoter competition for T7 RNAP is complicated by the fact that the T7 promoter element does not resemble bacterial operator elements. To expand our methodology to the study of T7 promoter systems, repressor DNA binding domains would have to be modified to recognize distinct half sites of the non-symmetric pT7 sequence. Such an experimental setup would likely require the co-expression of LacI heterodimers, increasing the complexity of the allocator.

Finally, the modeling of repressor kinetics and behaviours was also demonstrated to be sensitive to promoter burden, altering the apparent dynamic range and point of saturation of our tested inducer titration curves. Daber and Lewis published a series of impressive papers detailing the assessment of repressor kinetics *in vivo*, enabling the thermodynamic assessment of repressor equilibria (53–55). Their approach provides a means to construct a thermodynamic based description of repressor phenotypes. However, these systems would likely be susceptible to similar promoter burden, observed in this study. Namely, we have demonstrated that expression profiles vary depending on the number of available transcription and translation resources (i.e. altering the apparent dynamic range, leakiness, sensitivity and EC_50_) which Daber and Lewis use to back-calculate repressor kinetics. Thus, in light of other seminal studies, the incorporation of more accurate metrics for the utilization of various resource budgets will enable the development of better predictive tools for design automation in synthetic biology and metabolic engineering (56).

## Supplementary Material

gkaa734_Supplemental_File
