# Supplementary material for: Engineered signal-coupled inducible promoters: measuring the apparent RNA-polymerase resource budget
Source: Nucleic Acids Res. 2020 Sep 5;48(17):9995–10012. doi: 10.1093/nar/gkaa734 (PMC7515704; doi:10.1093/nar/gkaa734)
Supplement: gkaa734_Supplemental_File [file gkaa734_supplemental_file.pdf]

Engineered signal-coupled inducible promoters: measuring the apparent RNA-polymerase resource budget

James A. Davey[1] and Corey J. Wilson[1†]

[1]Georgia Institute of Technology, School of Chemical & Biomolecular Engineering

†To whom correspondence should be addressed: Corey J. Wilson, Georgia Institute of Technology, School of Chemical & Biomolecular Engineering, 311 Ferst Drive, Atlanta, GA 30332-0100. E-Mail: corey.wilson@chbe.gatech.edu

Supplementary Figure S1. Cartoon Plasmid Maps, Operator Placement and Promoter Alignments

Supplementary Figure S2. Expression Plots:

A. –pGFP / −pRFP	B. pUV5 GFP / −pRFP	C. pSym GFP / −pRFP
D. pNull GFP / −pRFP	E. pLac GFP / −pRFP	F. pTrc GFP / −pRFP
G. –pGFP / pLac RFP	H. pUV5 GFP / pLac RFP	I. pSym GFP / pLac RFP
J. pNull GFP / pLac RFP	K. pLac GFP / pLac RFP	L. pTrc GFP / pLac RFP
M. –pGFP / pNull RFP	N. pLac GFP / pNull RFP	O. −pGFP / −pRFP / LacI(TAN)
P. −pGFP / pLac RFP / LacI(TAN)	Q. −pGFP / pTTA RFP / LacI(TAN)	

Supplementary Table S1. Regression Statistics for Corresponding Plots in Figure S2.A−N

Supplementary Table S2. pValues Statistics for Corresponding Regression Statistic Slopes in Table S1

Supplementary Table S3. Regression Statistics for Corresponding Plots in Figure S2.O−Q

Supplementary Table S4. pValues Statistics Corresponding Regression Statistic Slopes in Table S3

Supplementary Figure S3. Dose Response Expression Plots:

A. LacI(YQR)	regulation of	–pGFP / pLac RFP,	pNull GFP / pLac RFP,	pTrc GFP / pLac RFP
B. LacI(TAN)	regulation of	–pGFP / pTTA RFP,	pNull GFP / pTTA RFP,	pTrc GFP / pTTA RFP
C. LacI(IAN)	regulation of	–pGFP / pTTA RFP,	pNull GFP / pTTA RFP,	pTrc GFP / pTTA RFP
D. LacI(VAN)	regulation of	–pGFP / pTTA RFP,	pNull GFP / pTTA RFP,	pTrc GFP / pTTA RFP

Supplementary Table S5. IPTG Inducer Titration Curve Parameters

Figure S1

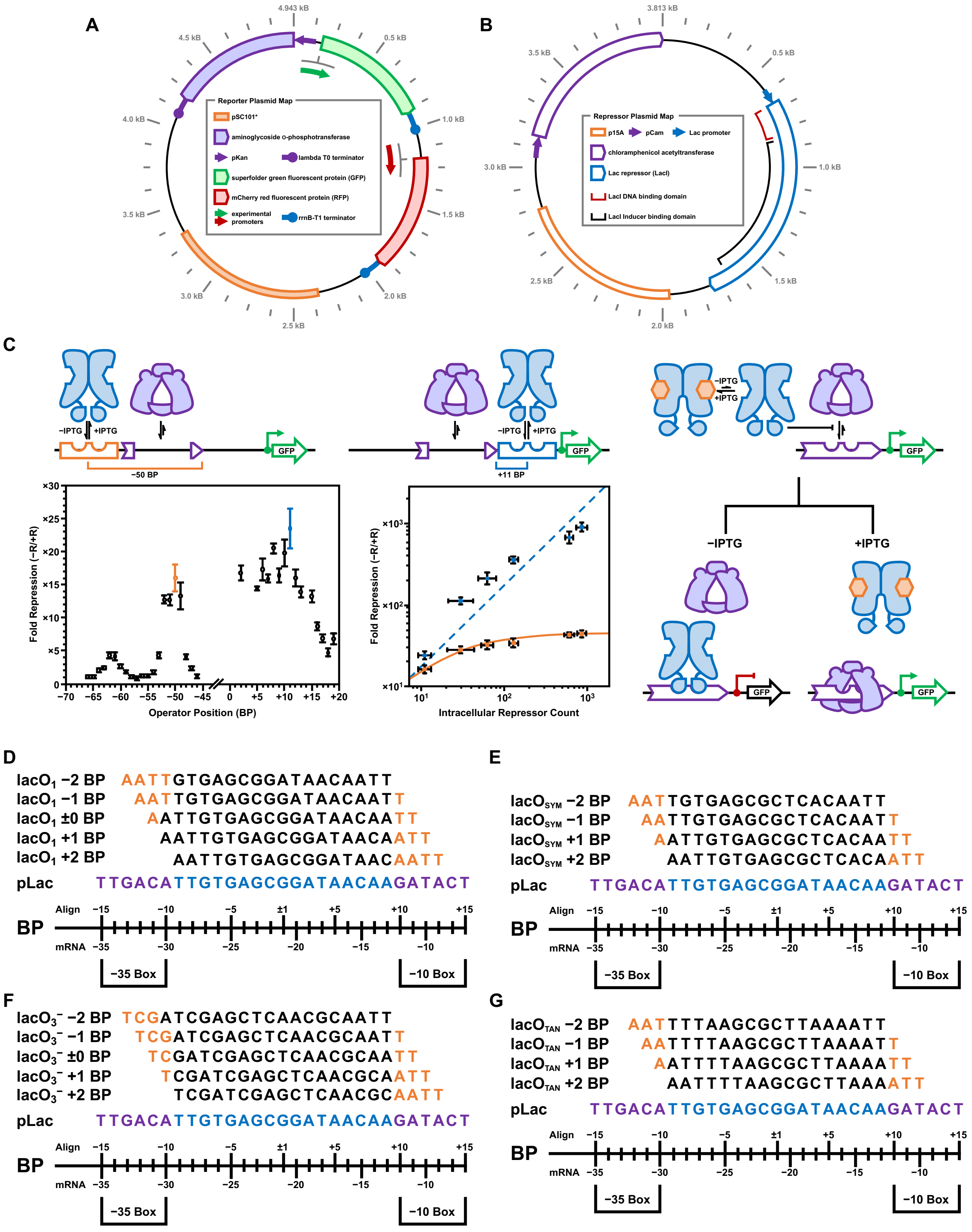

A
Reporter Plasmid Map
pSC101*
aminoglycoside o-phosphotransferase
pKan
lambda T0 terminator
superfolder green fluorescent protein (GFP)
mCherry red fluorescent protein (RFP)
experimental promoters
rrnB-T1 terminator
B
Repressor Plasmid Map
p15A
pCam
Lac promoter
chloramphenicol acetyltransferase
Lac repressor (LacI)
LacI DNA binding domain
LacI Inducer binding domain
C
Fold Repression (−R/+R)
Operator Position (BP)
Intracellular Repressor Count
−IPTG
+IPTG
GFP
D
lacO1 −2 BP AATTGTGAGCGGATAACAATT
lacO1 −1 BP AATTGTGAGCGGATAACAATT
lacO1 ±0 BP AATTGTGAGCGGATAACAATT
lacO1 +1 BP AATTGTGAGCGGATAACAATT
lacO1 +2 BP AATTGTGAGCGGATAACAATT
pLac TTGACATTGTGAGCGGATAACAAGATACT
BP
−35 Box
−10 Box
E
lacOSYM −2 BP AATTGTGAGCGCTCACAATT
lacOSYM −1 BP AATTGTGAGCGCTCACAATT
lacOSYM +1 BP AATTGTGAGCGCTCACAATT
lacOSYM +2 BP AATTGTGAGCGCTCACAATT
pLac TTGACATTGTGAGCGGATAACAAGATACT
F
lacO3− −2 BP TCGATCGAGCTCAACGCAATT
lacO3− −1 BP TCGATCGAGCTCAACGCAATT
lacO3− ±0 BP TCGATCGAGCTCAACGCAATT
lacO3− +1 BP TCGATCGAGCTCAACGCAATT
lacO3− +2 BP TCGATCGAGCTCAACGCAATT
pLac TTGACATTGTGAGCGGATAACAAGATACT
G
lacOTAN −2 BP AATTTTAAGCGCTTAAAATT
lacOTAN −1 BP AATTTTAAGCGCTTAAAATT
lacOTAN +1 BP AATTTTAAGCGCTTAAAATT
lacOTAN +2 BP AATTTTAAGCGCTTAAAATT
pLac TTGACATTGTGAGCGGATAACAAGATACT

Figure S1. Plasmid maps detailing the general architecture of the (A) reporter and (B) repressor plasmids constructed and assayed in this study. All reporter plasmid variants contain copies of the genes for superfolder green fluorescent protein (GFP) and mCherry red fluorescent protein (RFP), each adjacent to identical rrnB T1 terminator sequences. Reporter plasmid variants were created by inserting combinations of various promoter cassettes reported in **Figure 1-2**. The repressor plasmid is responsible for donating the genetic material necessary to produce the LacI repressor (+LacI). The control repressor plasmid variant, referred to as –LacI, incorporates a V1* mutation preventing translation of the LacI gene from the transcribed mRNA, allowing for assessment of reporter plasmid output in the absence of LacI. All reporter plasmids express the aminoglycosie O-phosphotranferase gene conferring resistance to kanamycin and are replicated through the low copy number SC101* origin, while all repressor plasmids express the chloramphenicol acetyltransferase gene conferring resistance to chloramphenicol and are replicated via the 15A origin. The orthogonal selection markers and replication origins enable co-transformation and assaying of reporter and repressor plasmid pairs. Summary of results published by Garcia et al. (1)(C) who previously established that operator placement relative to the promoter site can alter the mechanism of repression and allow for simultaneous recruitment of repressor and RNA polymerase (RNAP) to the promoter cassette. Experimental design in this study (D-G) places the operator between the −35 and −10 promoter boxes to eliminate the possibility of simultaneous recruitment of both RNAP and LacI to the promoter. Sequence alignments of the $lacO_1$ (D), $lacO_{sym}$ (E), $lacO_{3-}$ (F), and $lacO_{tan}$ (G) operators were evaluated to facilitate our promoter design strategy.

Figure S2

-pGFP / -pRFP

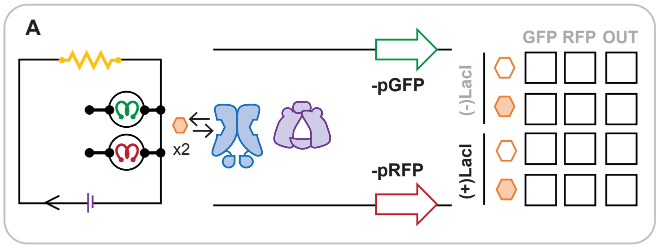

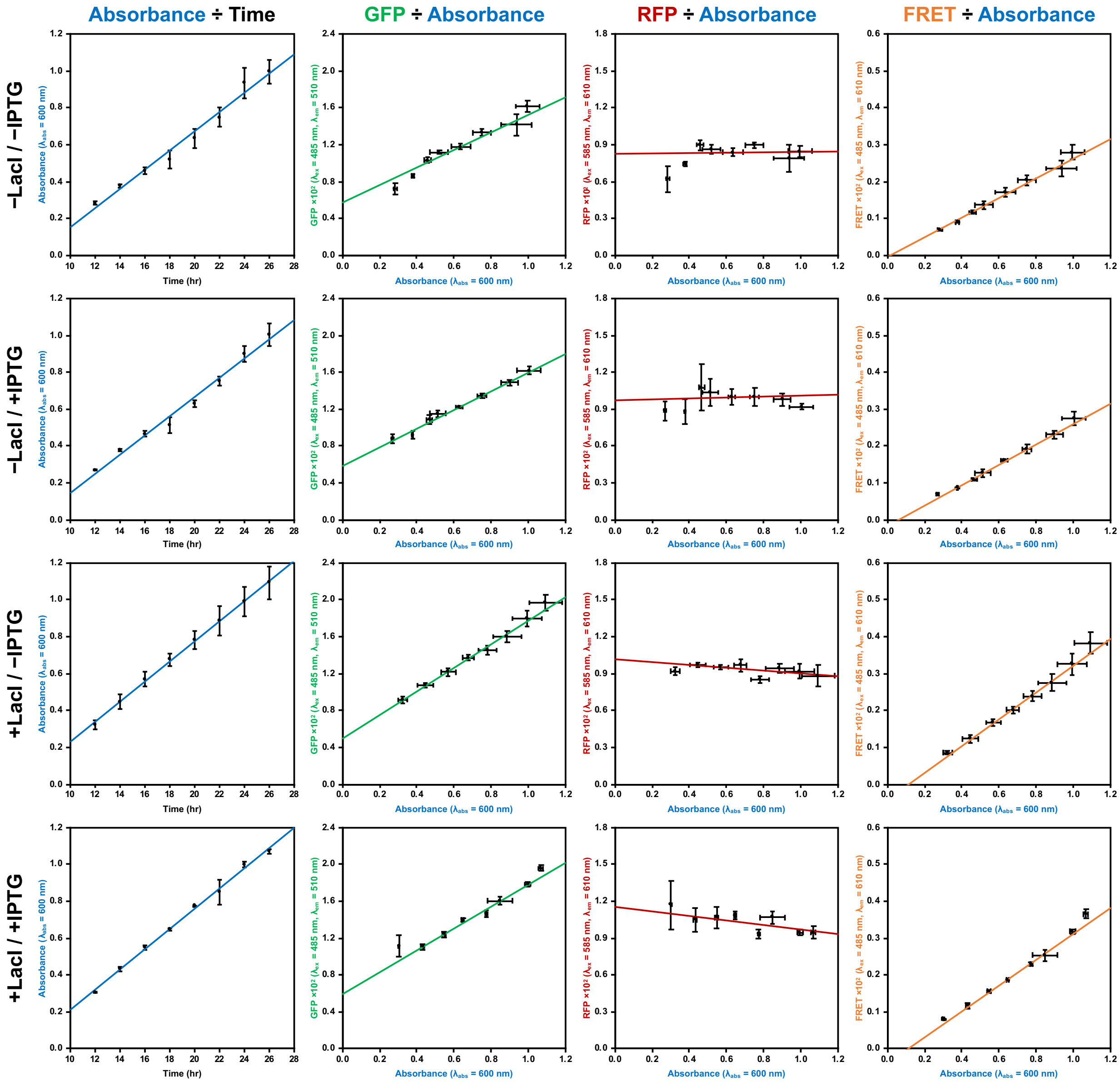

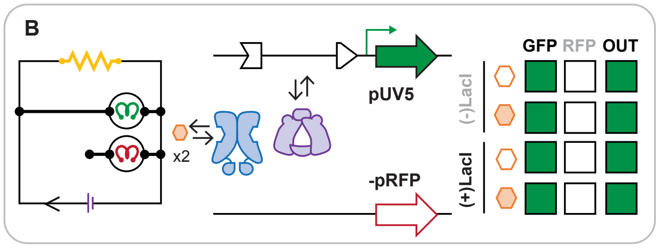

pUV5 GFP / -pRFP

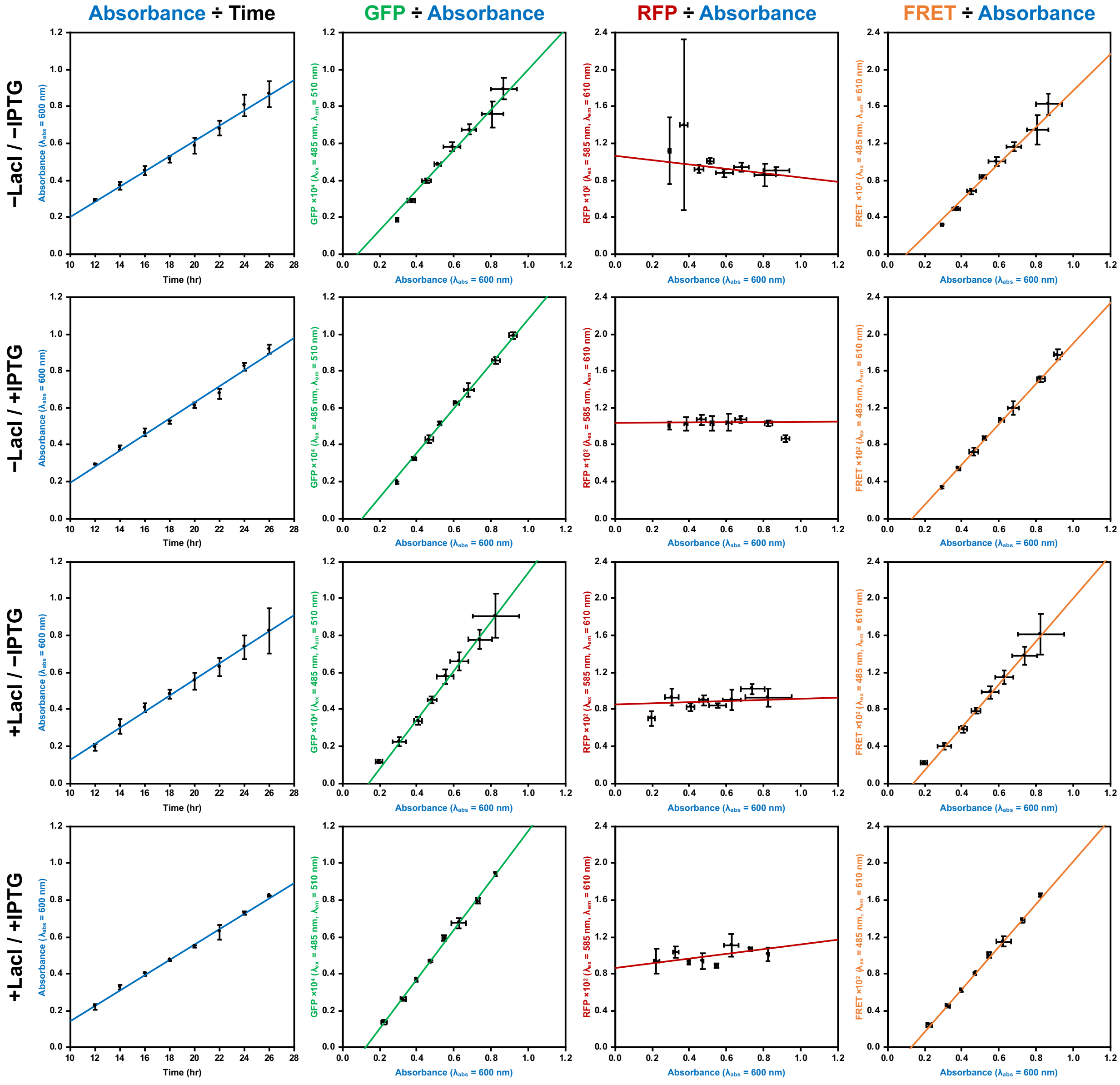

pSym GFP / -pRFP

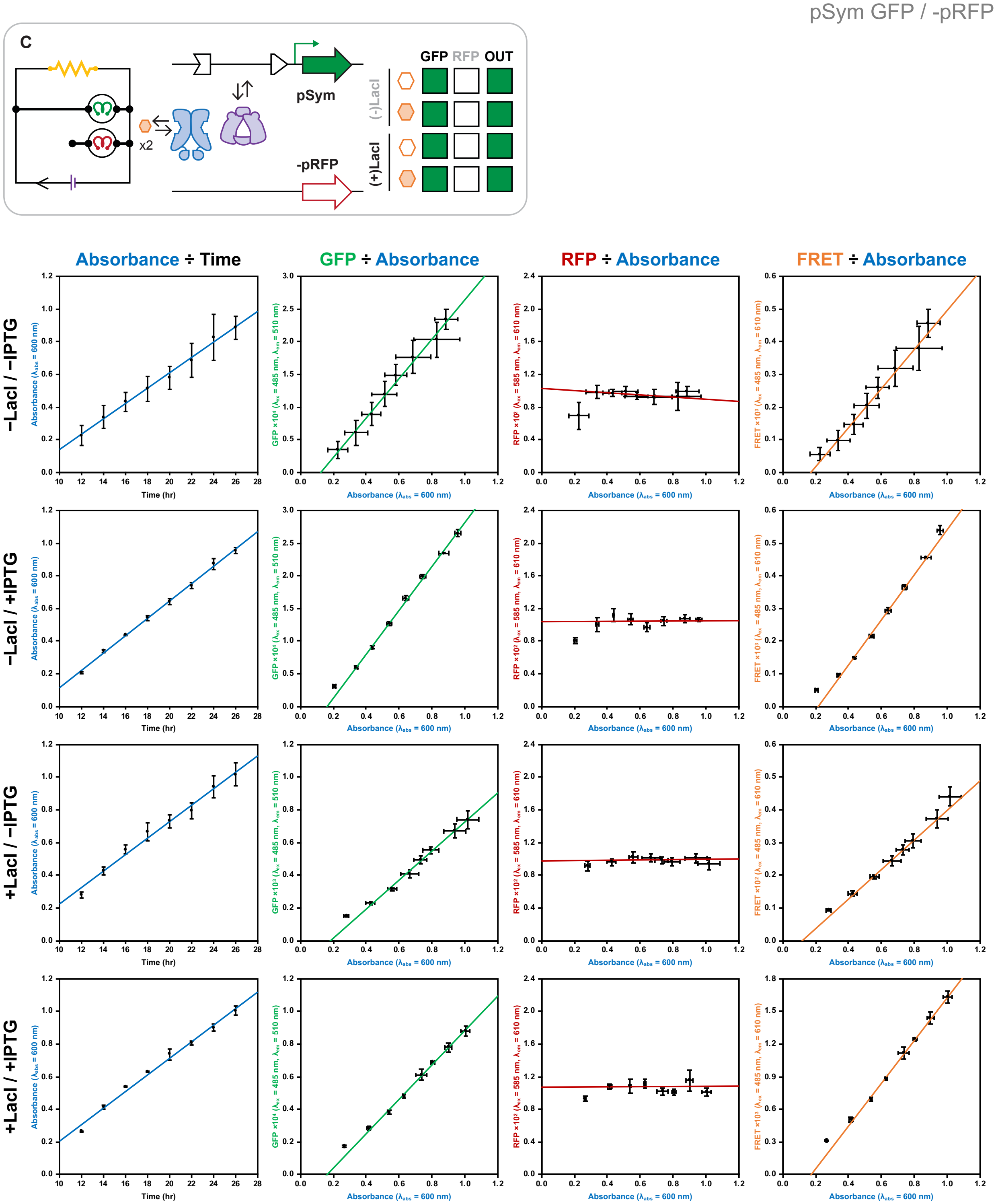

pNull GFP / -pRFP

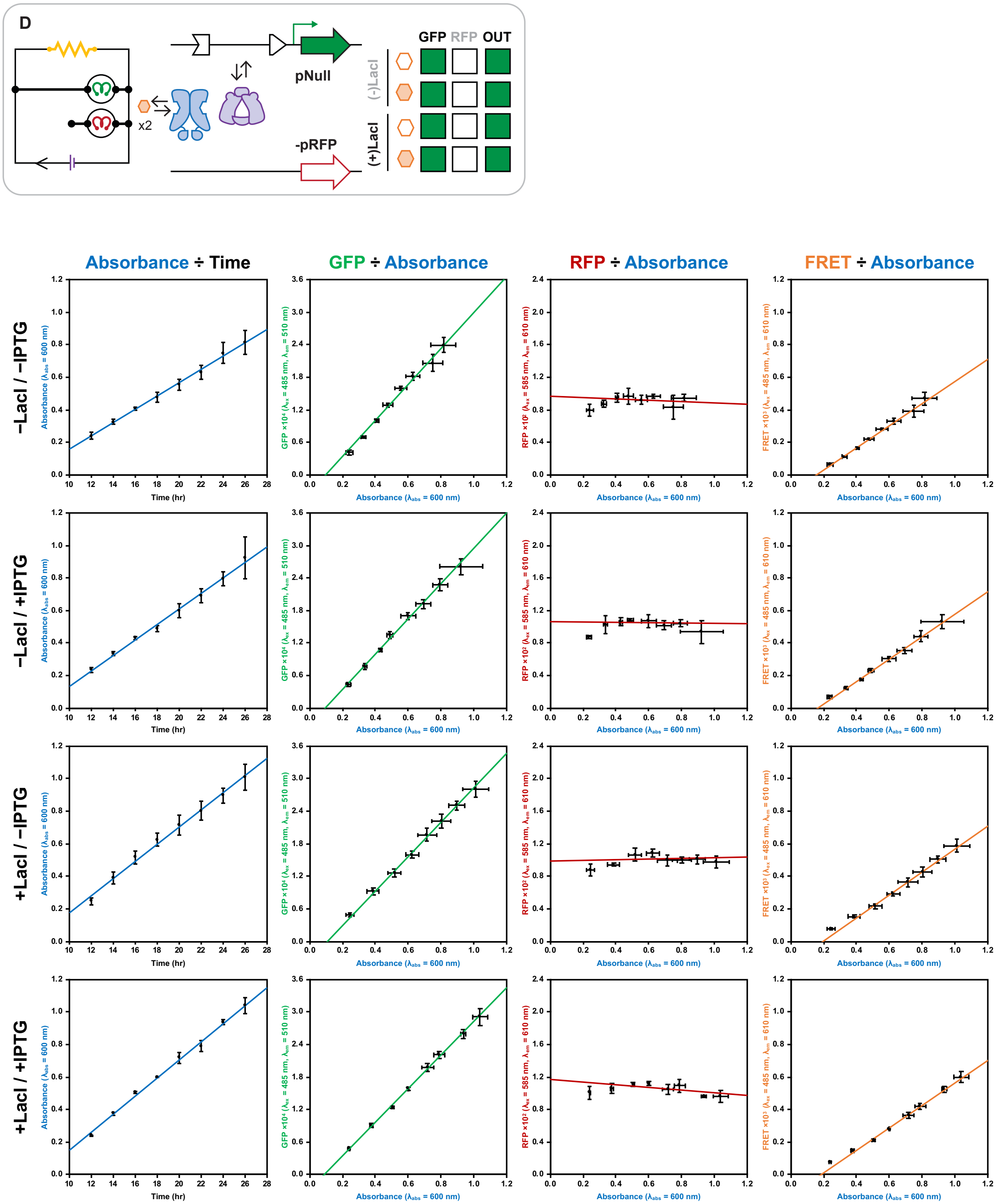

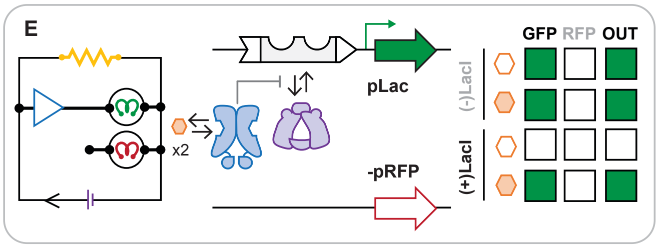

E
x2
pLac
-pRFP
GFP RFP OUT
(-)LacI
(+)LacI

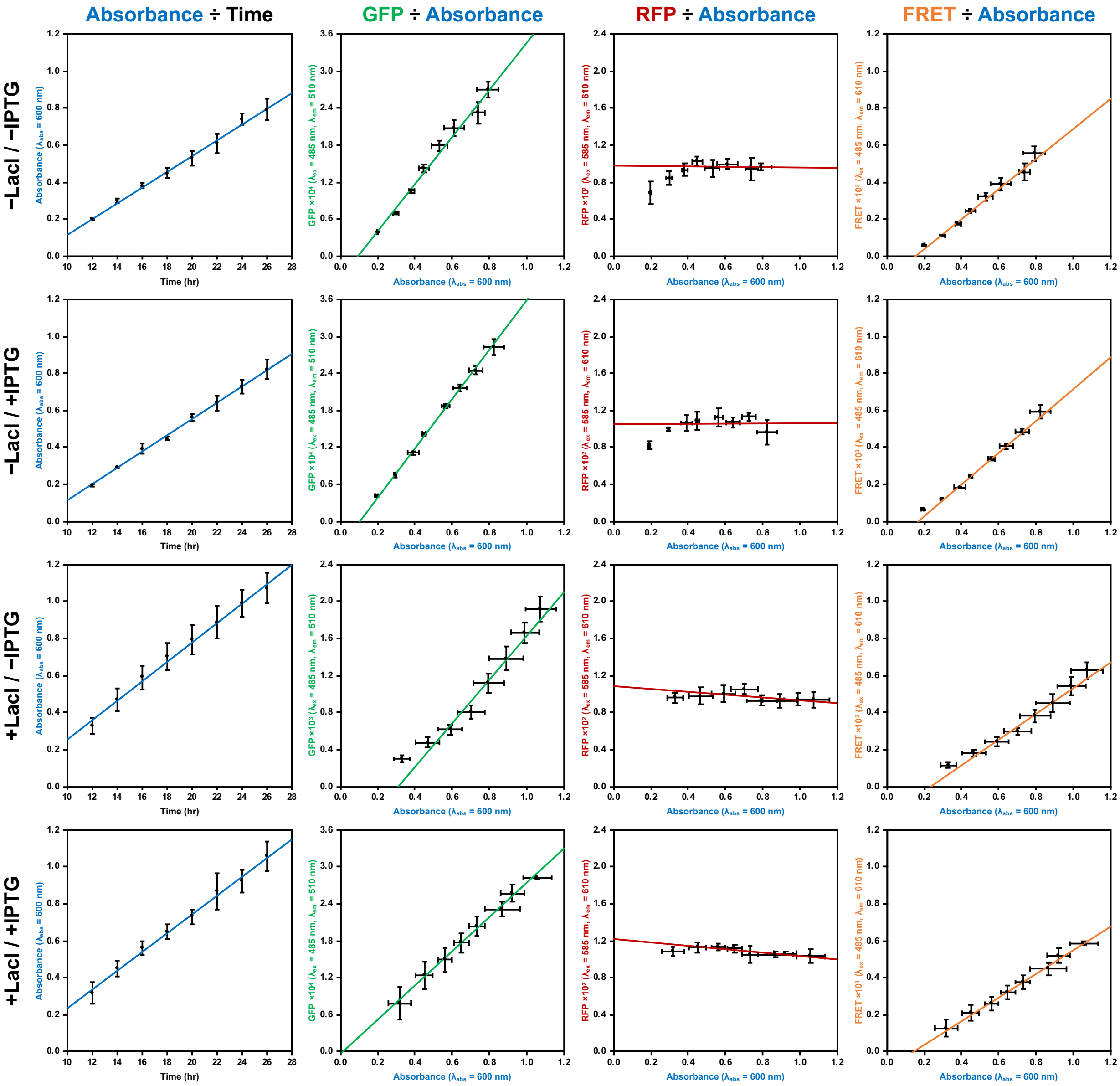

Absorbance ÷ Time
GFP ÷ Absorbance
RFP ÷ Absorbance
FRET ÷ Absorbance
−LacI / −IPTG
−LacI / +IPTG
+LacI / −IPTG
+LacI / +IPTG
Absorbance (λ_{abs} = 600 nm)
Time (hr)
GFP ×10⁴ (λ_{ex} = 485 nm, λ_{em} = 510 nm)
GFP ×10³ (λ_{ex} = 485 nm, λ_{em} = 510 nm)
RFP ×10² (λ_{ex} = 585 nm, λ_{em} = 610 nm)
FRET ×10² (λ_{ex} = 485 nm, λ_{em} = 610 nm)
FRET ×10³ (λ_{ex} = 485 nm, λ_{em} = 610 nm)

pTrc GFP / -pRFP

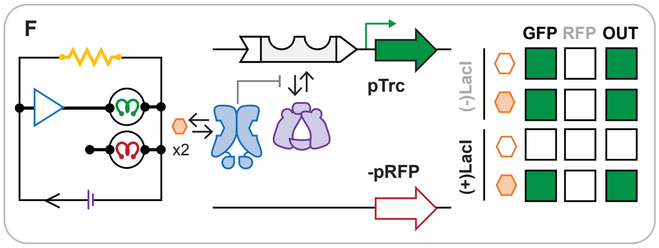

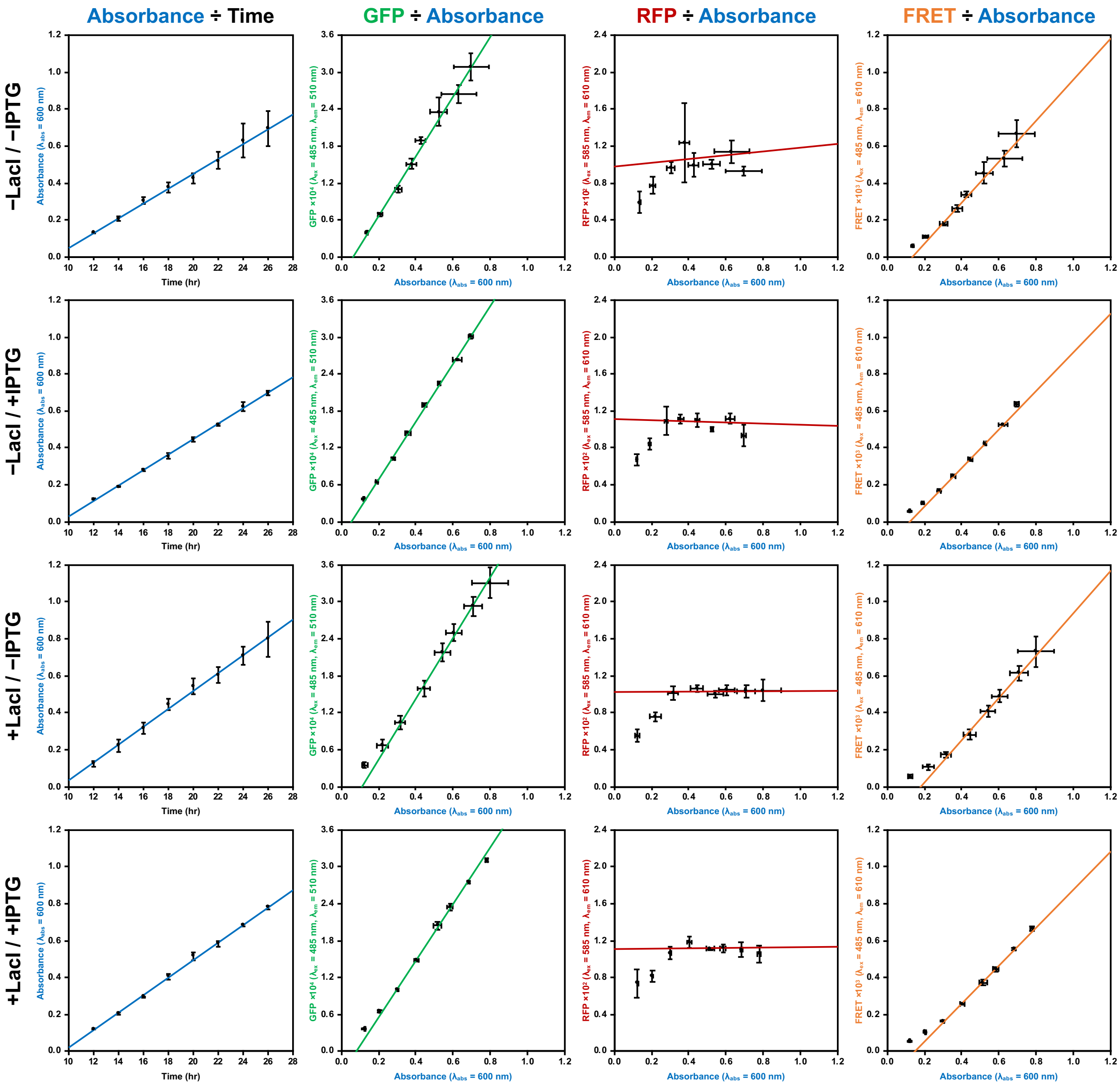

-pGFP / pLac RFP

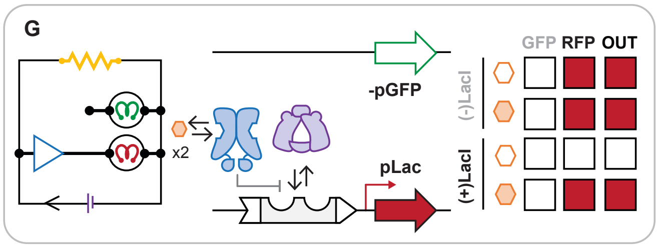

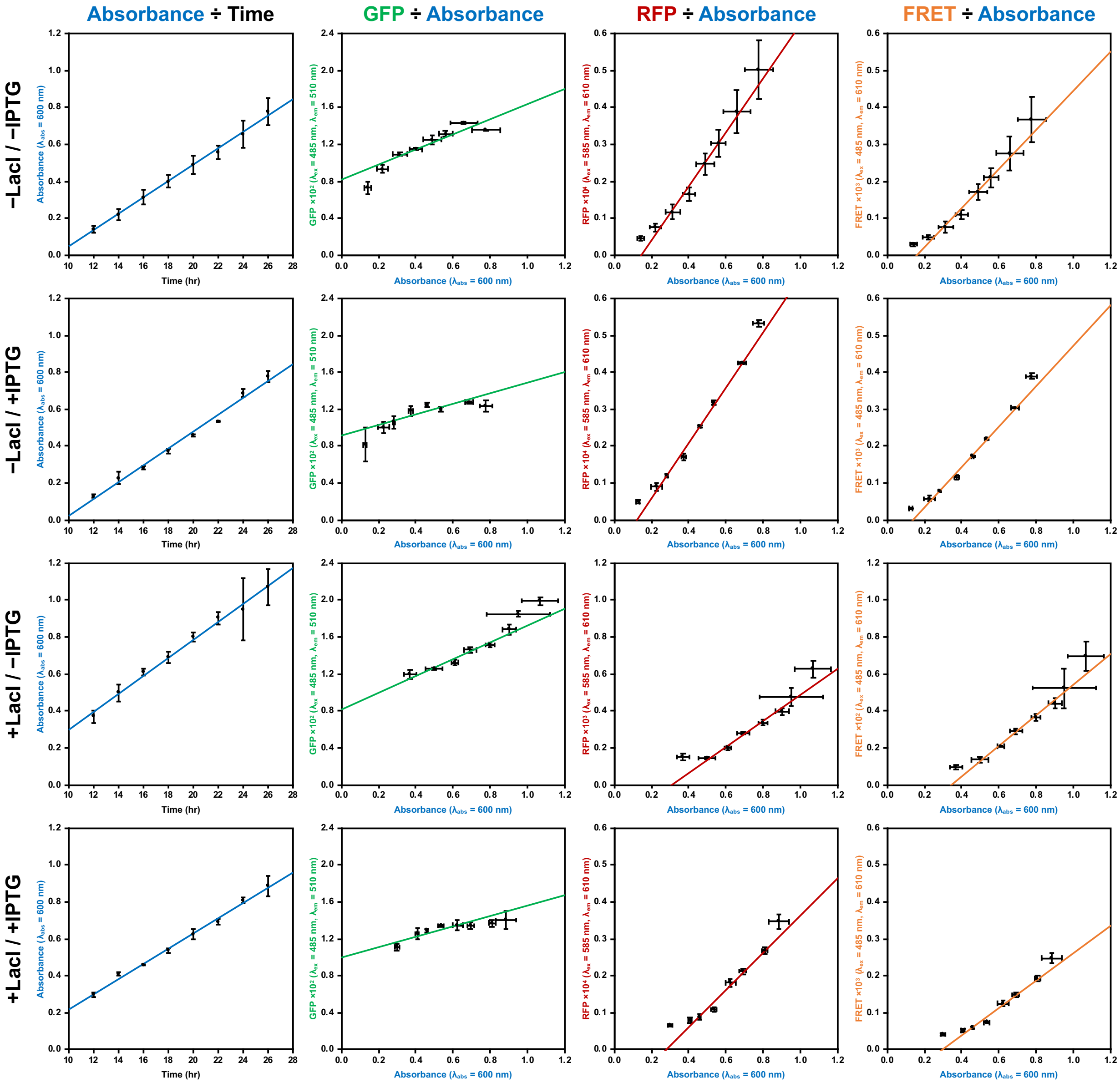

pUV5 GFP / pLac RFP

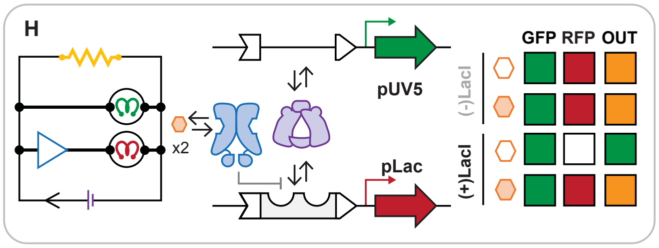

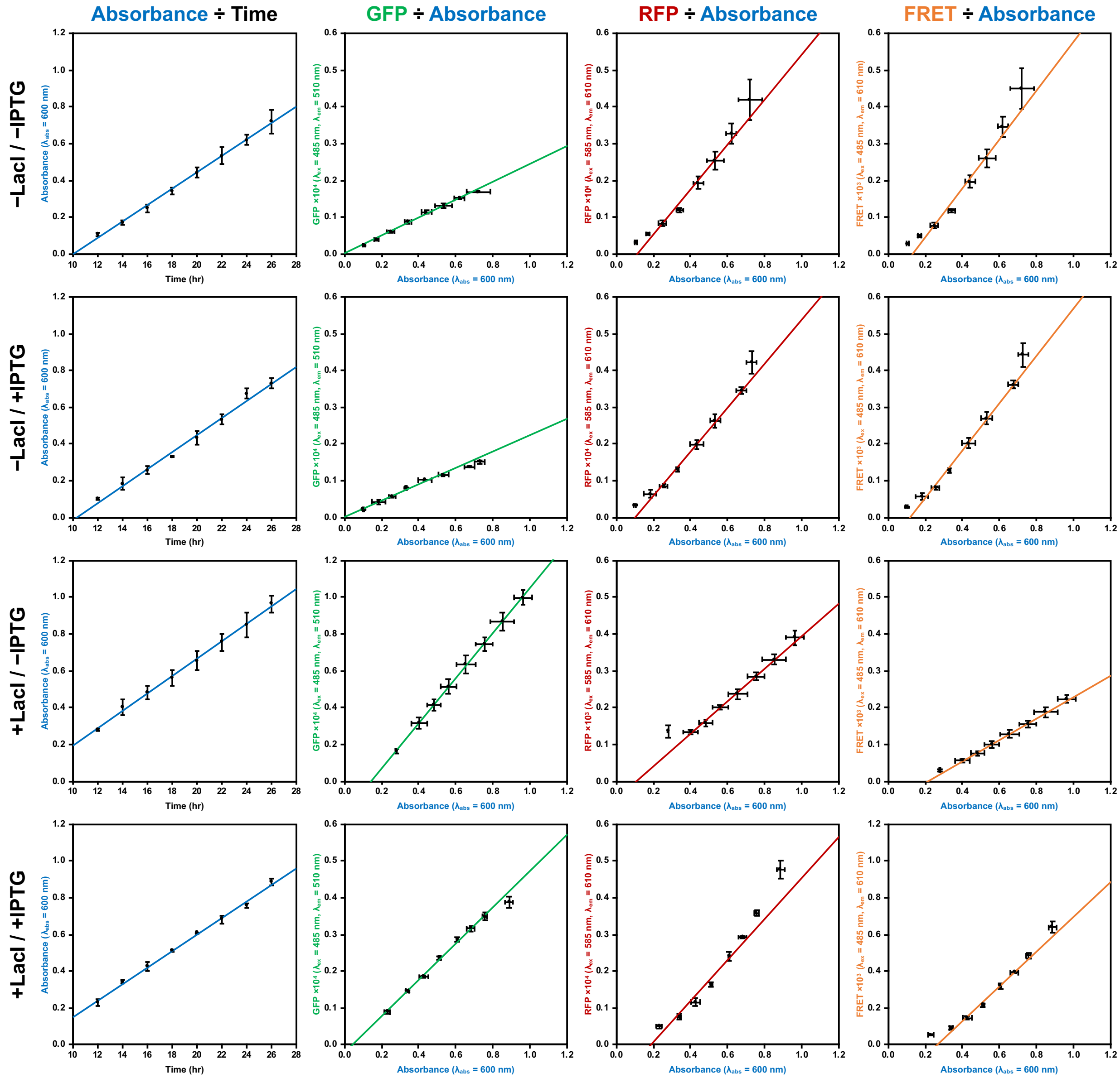

pSym GFP / pLac RFP

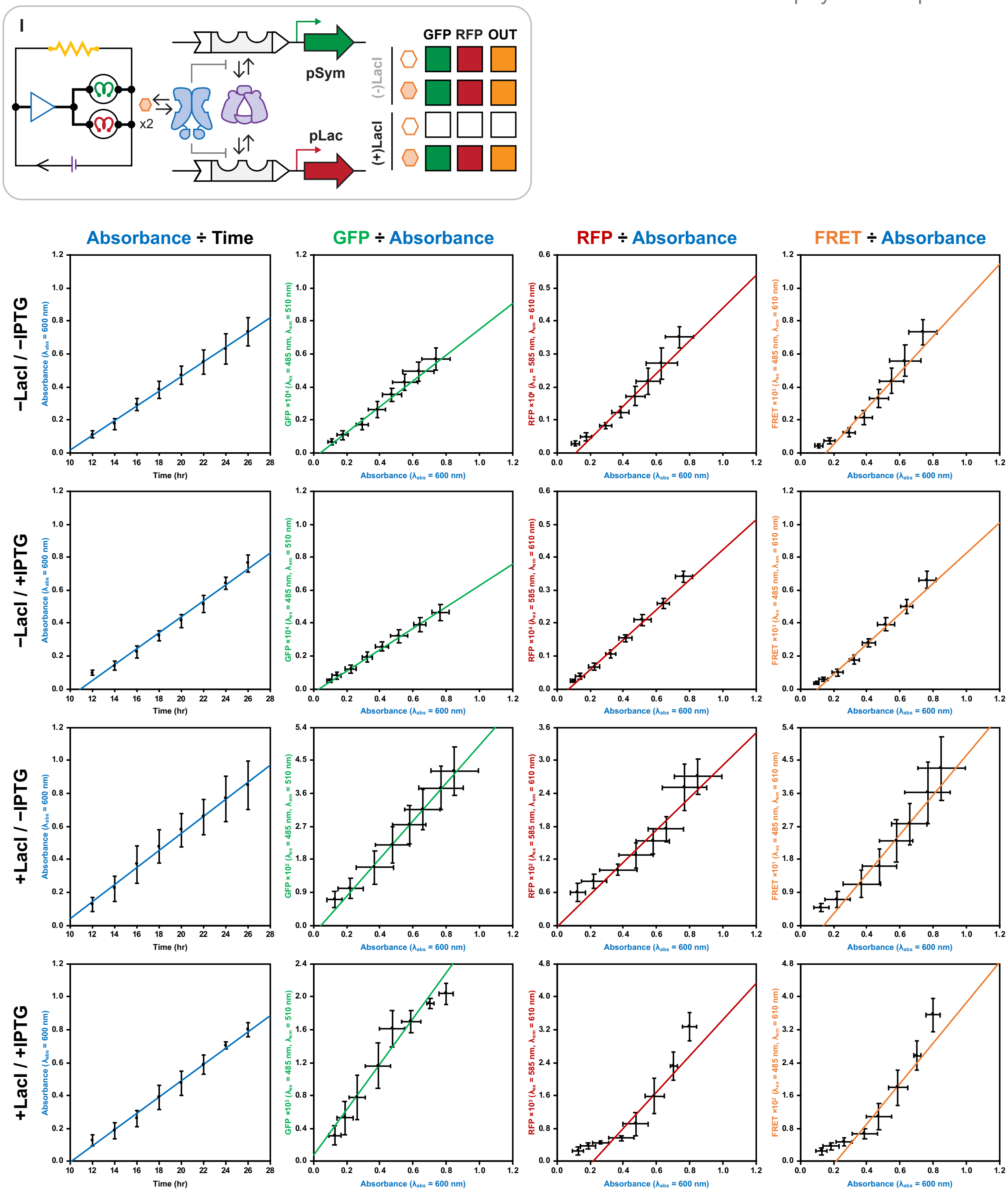

pNull GFP / pLac RFP

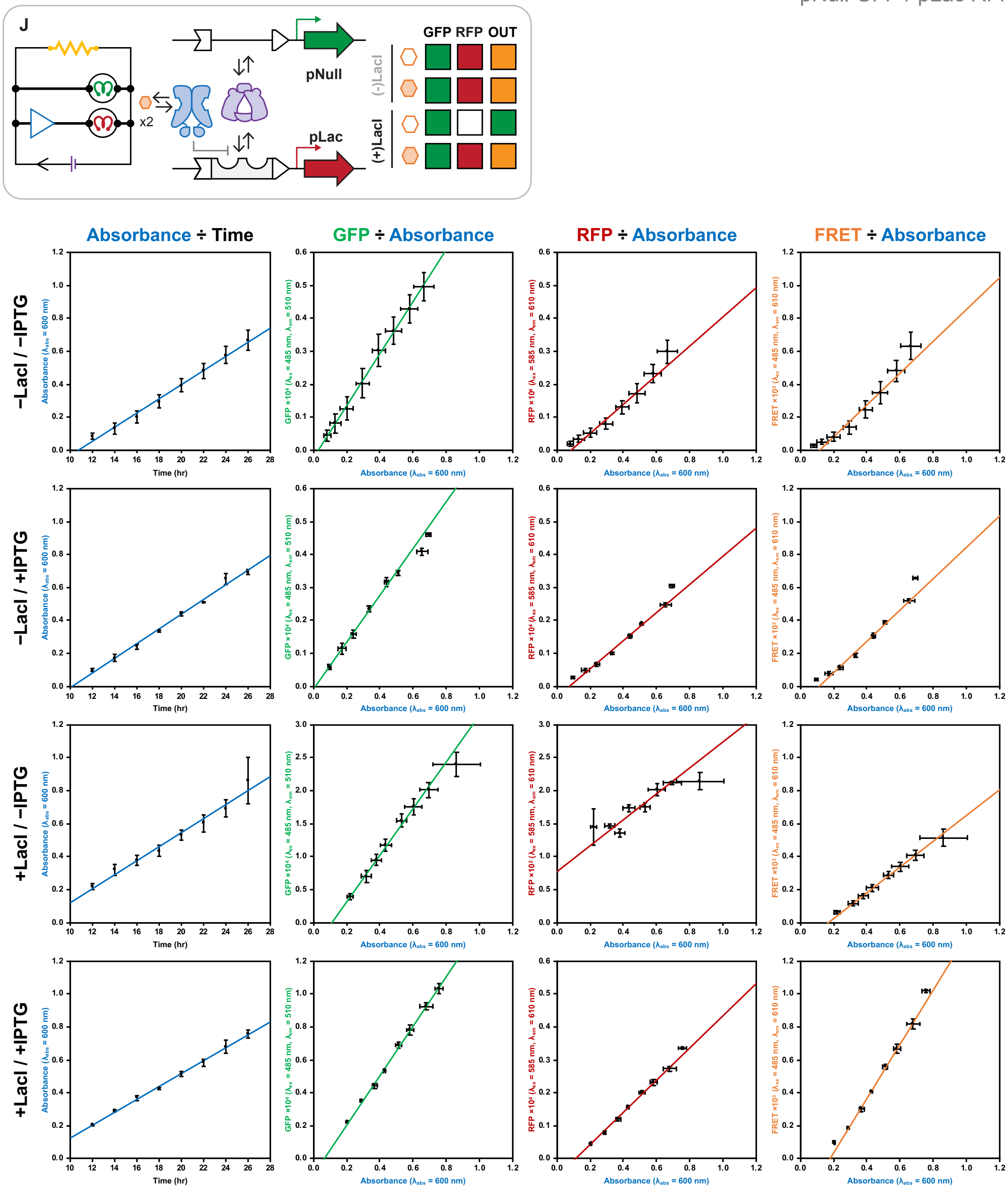

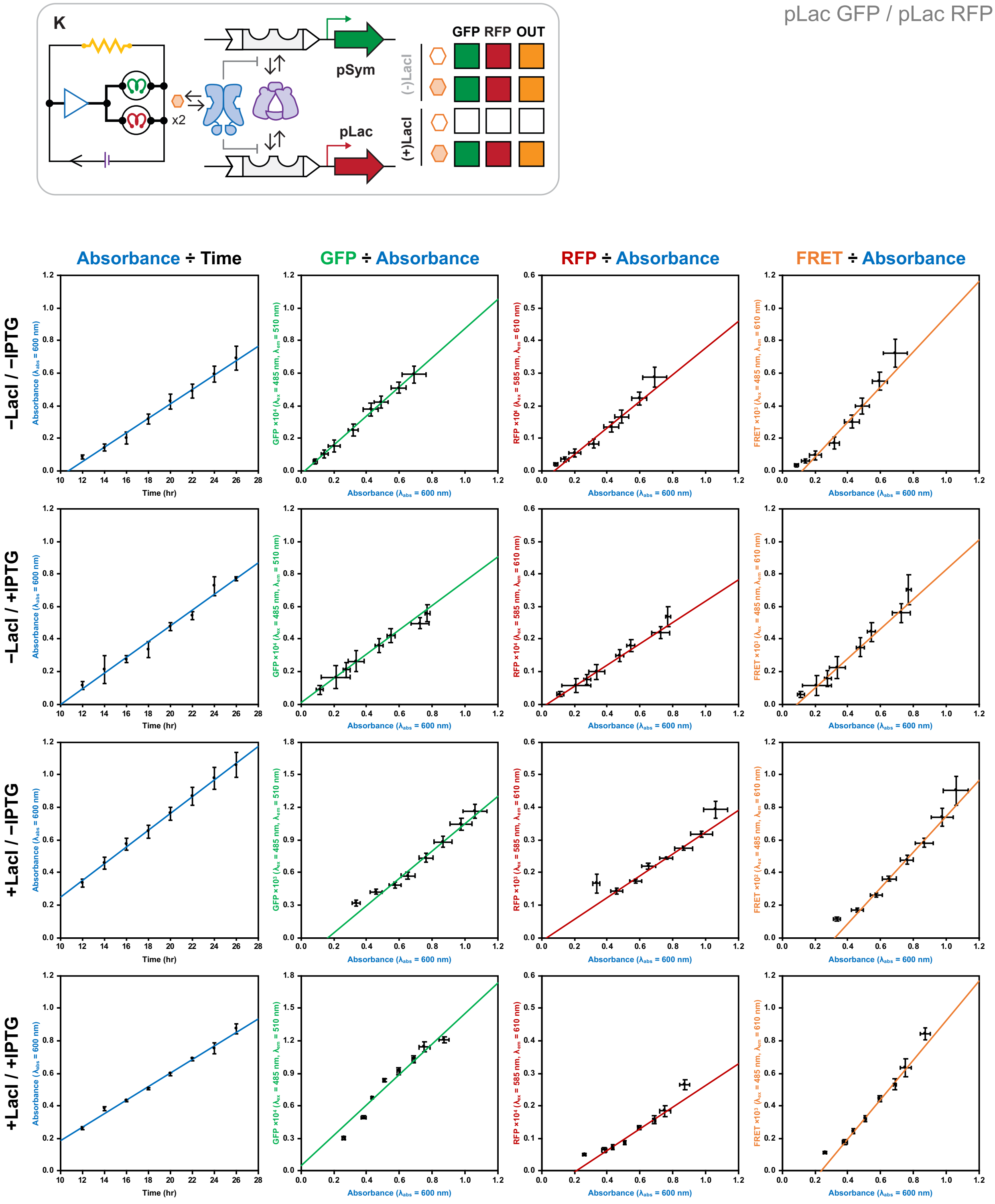
K
x2
pSym
pLac
GFP RFP OUT
(-)LacI
(+)LacI
Absorbance ÷ Time
GFP ÷ Absorbance
RFP ÷ Absorbance
FRET ÷ Absorbance
−LacI / −IPTG
−LacI / +IPTG
+LacI / −IPTG
+LacI / +IPTG
Time (hr)
Absorbance (λabs = 600 nm)
GFP ×10⁴ (λex = 485 nm, λem = 510 nm)
RFP ×10⁴ (λex = 585 nm, λem = 610 nm)
FRET ×10³ (λex = 485 nm, λem = 610 nm)

pTrc GFP / pLac RFP

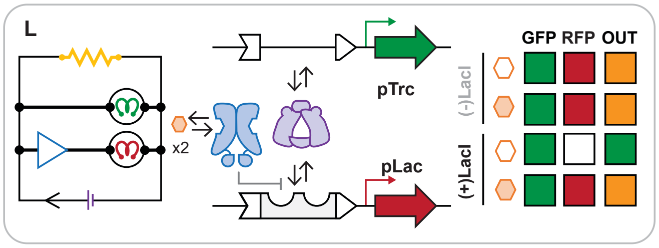

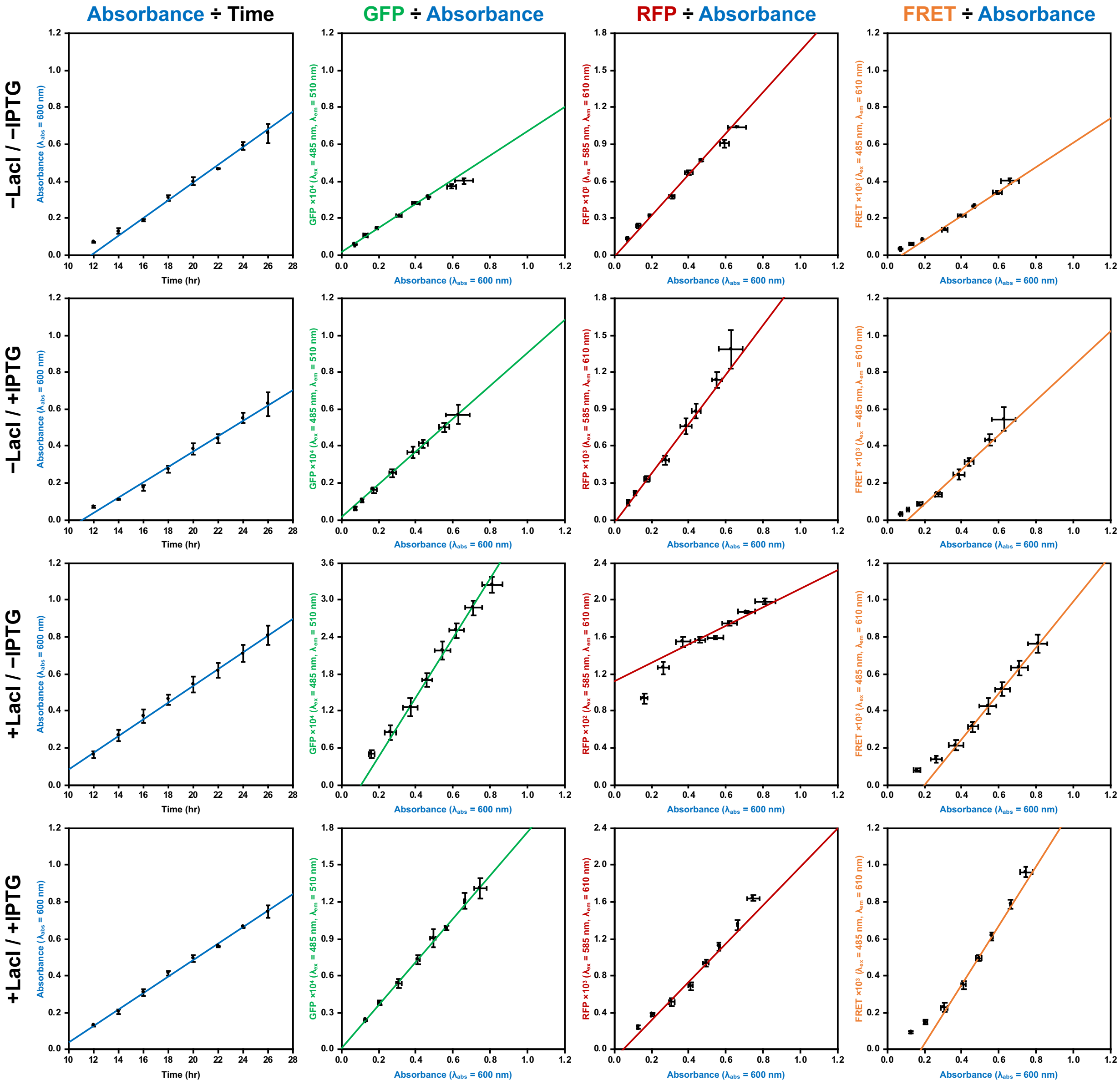

-pGFP / pNull RFP

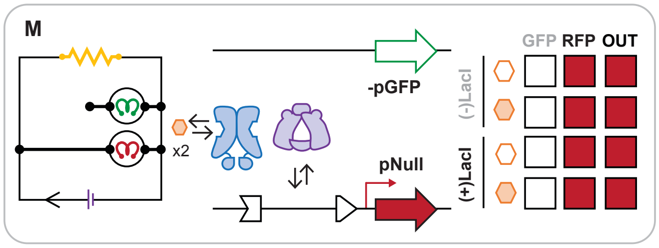

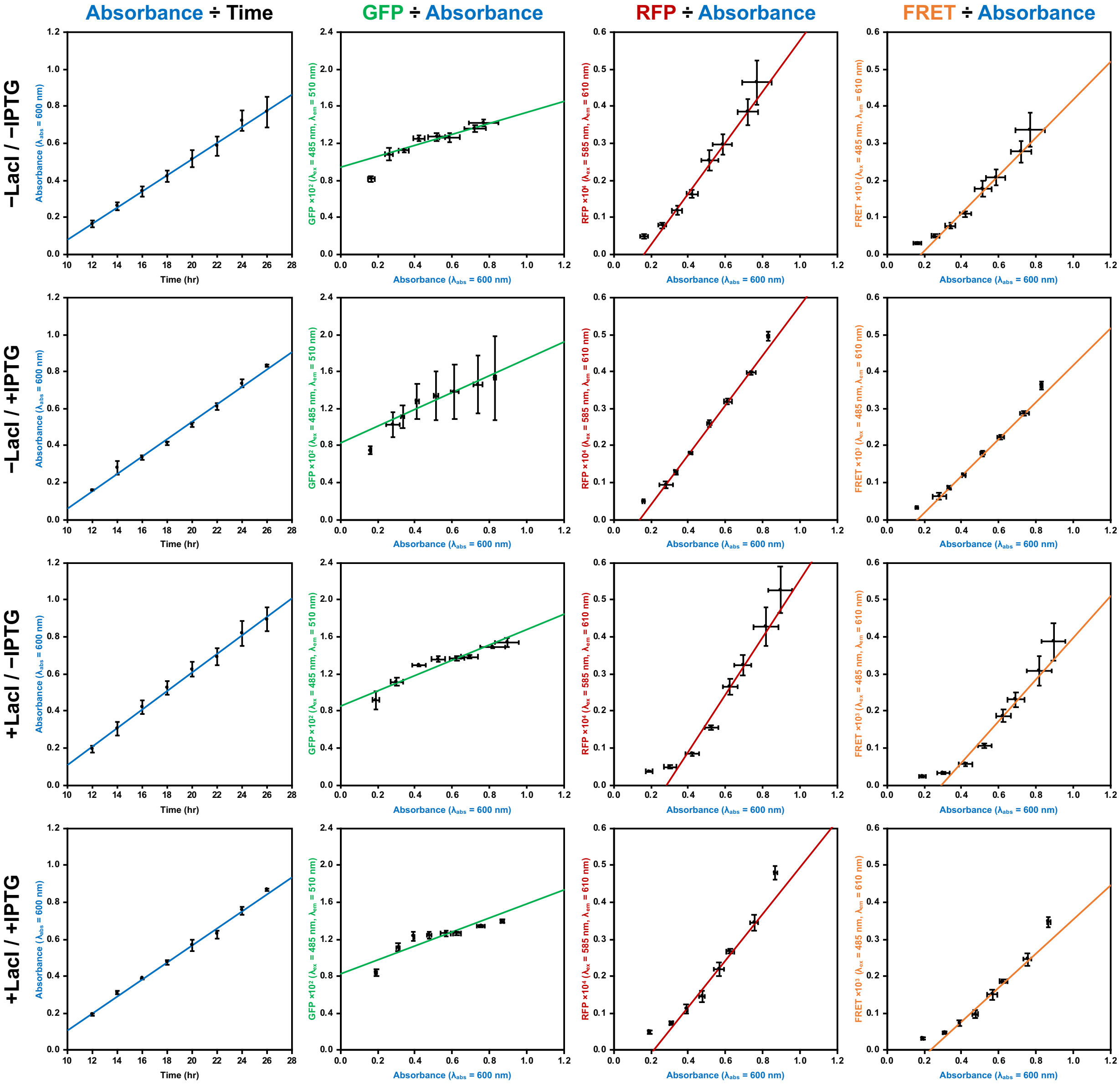

pLac GFP / pNull RFP

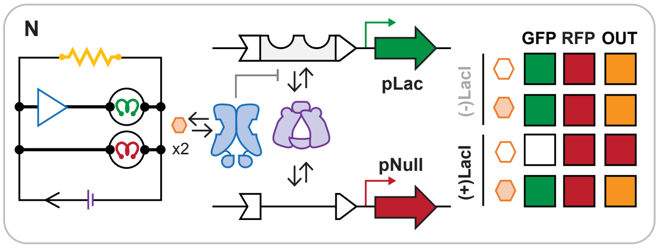

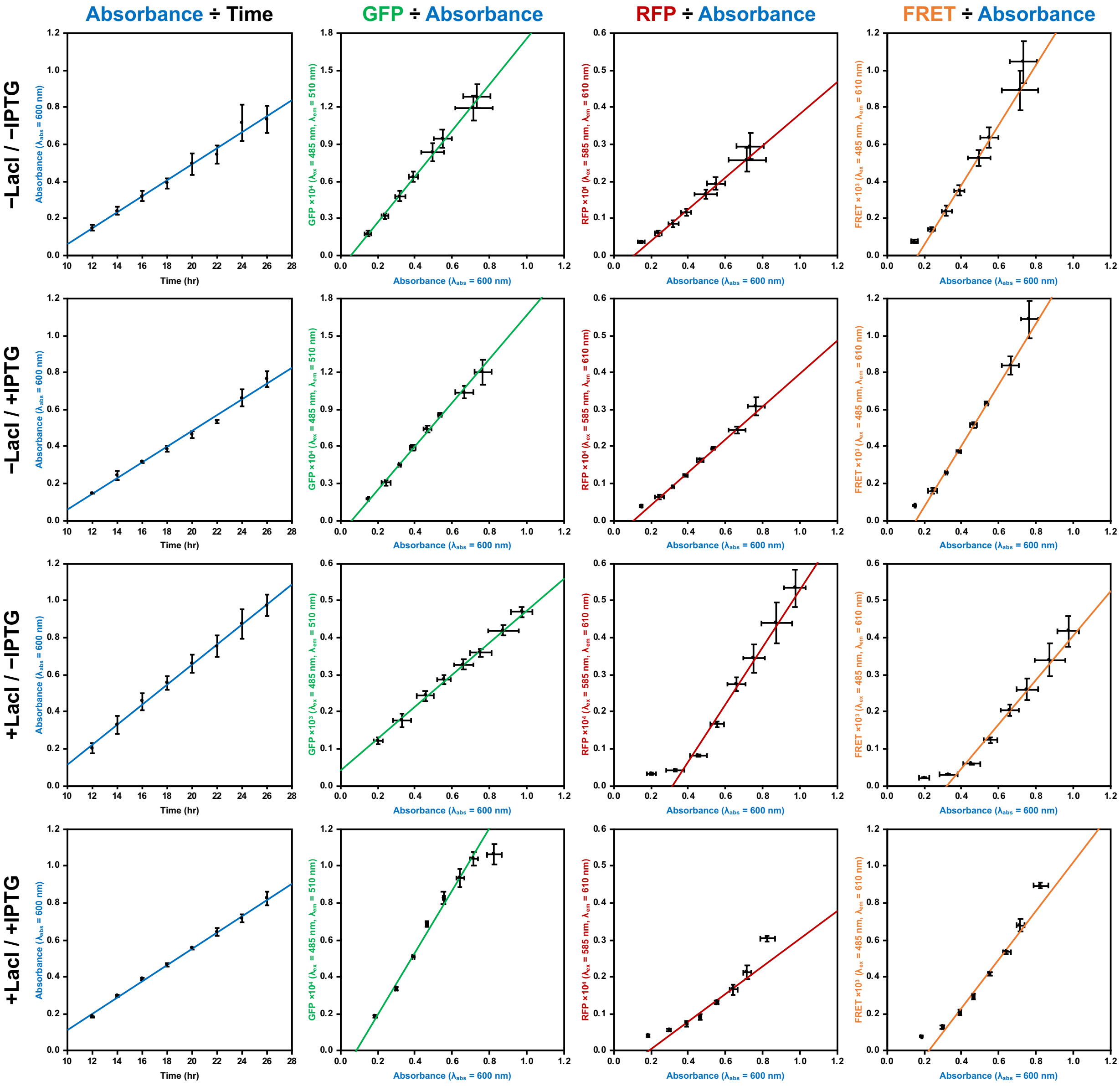

-pGFP / -pGFP: pLac: pTTA, RFP

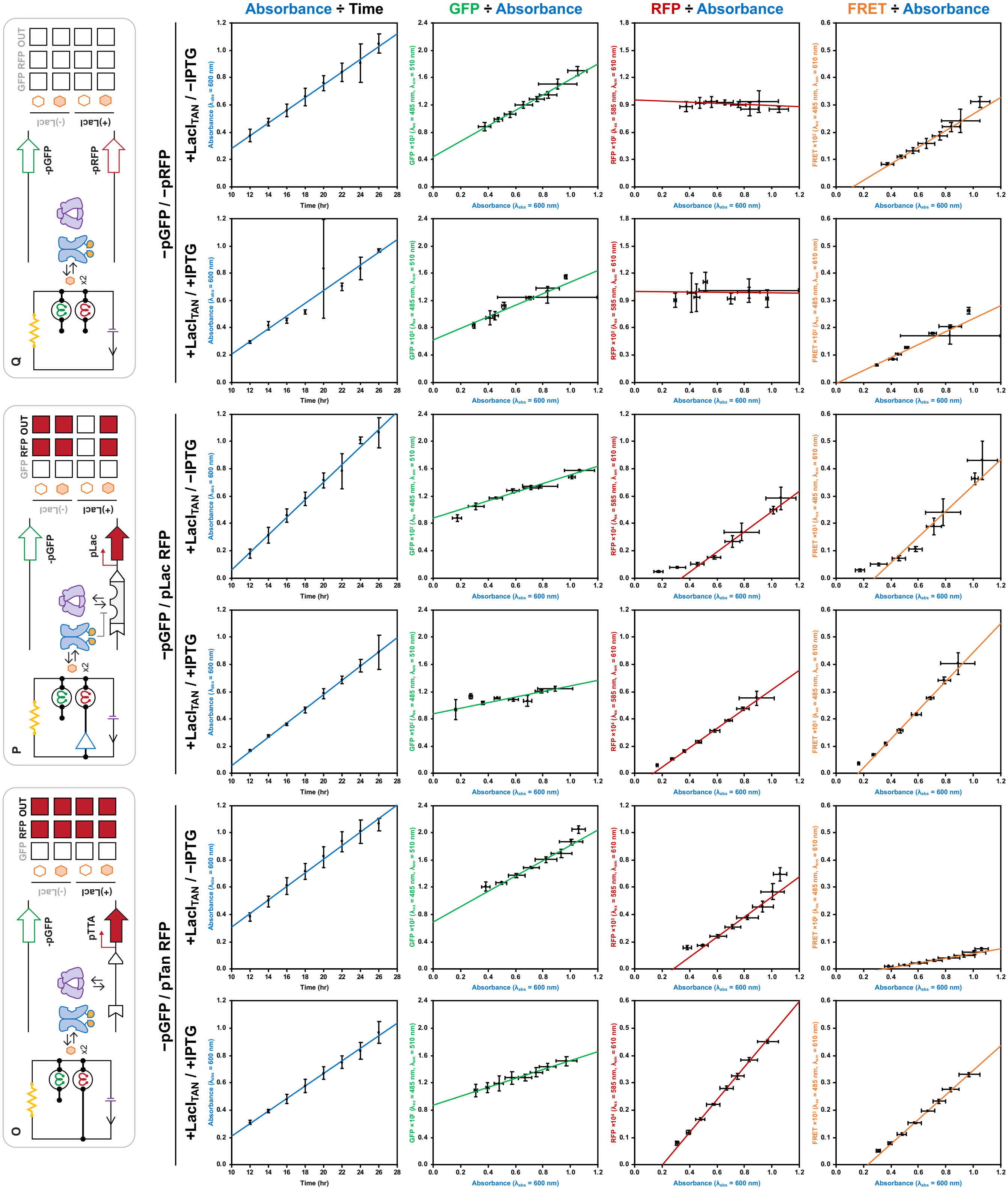

Figure S2. Time-course assays of reporter plasmids: A. –pGFP / −pRFP, B. pUV5 GFP / −pRFP, C. pSym GFP / −pRFP, D. pNull GFP / −pRFP, E. pLac GFP / −pRFP, F. pTrc GFP / −pRFP, G. –pGFP / pLac RFP, H. pUV5 GFP / pLac RFP, I. pSym GFP / pLac RFP, J. pNull GFP / pLac RFP, K. pLac GFP / pLac RFP, L. pTrc GFP / pLac RFP, M. –pGFP / pNull RFP, N. pLac GFP / pNull RFP, O. −pGFP / pTTA RFP / LacI(TAN), P. −pGFP / pLac RFP / LacI(TAN), Q. −pGFP / −pRFP / LacI(TAN). Plots are organized with columns (left to right) displaying culture density (λabs = 600 nm) as a function of time (hr), as well as green fluorescence (λex = 485 nm, λem = 510 nm, gain = 400), red fluorescence (λex = 585 nm, λem = 610 nm, gain = 400), and green-to-red FRET (λex = 485 nm, λem = 610 nm, gain = 400) as a function of culture density (λabs = 600 nm). Fluorescence values are reported in relative fluorescence units, with magnitude indicated on the vertical axis. Rows (top to bottom) detail expression profiles in the absence of both IPTG and functional LacI (−IPTG / −LacI), in the presence of 10 mM IPTG and absence of functional LacI (+IPTG / −LacI), in the absence of IPTG and the presence of functional LacI (−IPTG / +LacI), and in the presence of both 10 mM IPTG and functional LacI (+IPTG / +LacI). Linear fits to the data are provided on each plot to quantify expression parameters during steady state, reported in **Supplementary Tables S1** and **S3** for plots A−N and O−Q, respectively. Each set of plots includes a reference cartoon describing the reporter plasmid's setup (circuit diagram), molecular components (mechanism), and expected output (logic table).

Table S1. Regression statistics

Promoter Architecture		−LacI / −IPTG			−LacI / +IPTG			+LacI / −IPTG			+LacI / +IPTG		
GFP	RFP	R^2	Slope	Intercept	R^2	Slope	Intercept	R^2	Slope	Intercept	R^2	Slope	Intercept
**Time (X: hours) *versus* Absorbance (Y: λ_{abs} = 600 nm)**													
−pGFP	−pRFP	0.981	$5.2 \pm 0.3 \times 10^{-2}$	$-3.7 \pm 0.6 \times 10^{-1}$	0.986	$5.2 \pm 0.3 \times 10^{-2}$	$-3.7 \pm 0.5 \times 10^{-1}$	0.999	$5.5 \pm 0.1 \times 10^{-2}$	$-3.2 \pm 0.2 \times 10^{-1}$	0.997	$5.5 \pm 0.1 \times 10^{-2}$	$-3.4 \pm 0.3 \times 10^{-1}$
pUV5	−pRFP	0.993	$4.1 \pm 0.1 \times 10^{-2}$	$-2.1 \pm 0.3 \times 10^{-1}$	0.988	$4.4 \pm 0.2 \times 10^{-2}$	$-2.4 \pm 0.4 \times 10^{-1}$	0.996	$4.3 \pm 0.1 \times 10^{-2}$	$-3.1 \pm 0.2 \times 10^{-1}$	0.997	$4.1 \pm 0.1 \times 10^{-2}$	$-2.7 \pm 0.2 \times 10^{-1}$
pSym	−pRFP	0.993	$4.7 \pm 0.2 \times 10^{-2}$	$-3.3 \pm 0.3 \times 10^{-1}$	0.998	$5.3 \pm 0.1 \times 10^{-2}$	$-4.2 \pm 0.2 \times 10^{-1}$	0.986	$5.1 \pm 0.2 \times 10^{-2}$	$-2.9 \pm 0.5 \times 10^{-1}$	0.991	$5.1 \pm 0.2 \times 10^{-2}$	$-3.0 \pm 0.4 \times 10^{-1}$
pNull	−pRFP	0.996	$4.1 \pm 0.1 \times 10^{-2}$	$-2.5 \pm 0.2 \times 10^{-1}$	0.994	$4.8 \pm 0.1 \times 10^{-2}$	$-3.4 \pm 0.3 \times 10^{-1}$	0.993	$5.3 \pm 0.2 \times 10^{-2}$	$-3.5 \pm 0.3 \times 10^{-1}$	0.996	$5.6 \pm 0.1 \times 10^{-2}$	$-4.1 \pm 0.3 \times 10^{-1}$
pLac	−pRFP	0.995	$4.2 \pm 0.1 \times 10^{-2}$	$-3.1 \pm 0.2 \times 10^{-1}$	0.998	$4.4 \pm 0.1 \times 10^{-2}$	$-3.3 \pm 0.2 \times 10^{-1}$	0.994	$5.2 \pm 0.2 \times 10^{-2}$	$-2.7 \pm 0.3 \times 10^{-1}$	0.995	$5.1 \pm 0.2 \times 10^{-2}$	$-2.7 \pm 0.3 \times 10^{-1}$
pTrc	−pRFP	0.995	$4.0 \pm 0.1 \times 10^{-2}$	$-3.5 \pm 0.2 \times 10^{-1}$	0.999	$4.2 \pm 0.1 \times 10^{-2}$	$-3.9 \pm 0.1 \times 10^{-1}$	0.996	$4.8 \pm 0.1 \times 10^{-2}$	$-4.5 \pm 0.2 \times 10^{-1}$	0.998	$4.7 \pm 0.1 \times 10^{-2}$	$-4.5 \pm 0.2 \times 10^{-1}$
−pGFP	pLac	0.997	$4.4 \pm 0.1 \times 10^{-2}$	$-4.0 \pm 0.2 \times 10^{-1}$	0.989	$4.6 \pm 0.2 \times 10^{-2}$	$-4.4 \pm 0.4 \times 10^{-1}$	0.993	$4.8 \pm 0.2 \times 10^{-2}$	$-1.9 \pm 0.3 \times 10^{-1}$	0.995	$4.1 \pm 0.1 \times 10^{-2}$	$-1.9 \pm 0.2 \times 10^{-1}$
pUV5	pLac	0.997	$4.5 \pm 0.1 \times 10^{-2}$	$-4.5 \pm 0.2 \times 10^{-1}$	0.991	$4.6 \pm 0.2 \times 10^{-2}$	$-4.7 \pm 0.4 \times 10^{-1}$	0.997	$4.7 \pm 0.1 \times 10^{-2}$	$-2.8 \pm 0.2 \times 10^{-1}$	0.997	$4.5 \pm 0.1 \times 10^{-2}$	$-3.0 \pm 0.2 \times 10^{-1}$
pSym	pLac	0.998	$4.5 \pm 0.1 \times 10^{-2}$	$-4.3 \pm 0.2 \times 10^{-1}$	0.987	$4.8 \pm 0.2 \times 10^{-2}$	$-5.3 \pm 0.5 \times 10^{-1}$	0.995	$5.2 \pm 0.2 \times 10^{-2}$	$-4.8 \pm 0.3 \times 10^{-1}$	0.994	$5.0 \pm 0.2 \times 10^{-2}$	$-5.0 \pm 0.3 \times 10^{-1}$
pNull	pLac	0.994	$4.3 \pm 0.1 \times 10^{-2}$	$-4.7 \pm 0.3 \times 10^{-1}$	0.992	$4.5 \pm 0.2 \times 10^{-2}$	$-4.6 \pm 0.3 \times 10^{-1}$	0.978	$4.2 \pm 0.3 \times 10^{-2}$	$-3.0 \pm 0.5 \times 10^{-1}$	0.998	$3.9 \pm 0.1 \times 10^{-2}$	$-2.6 \pm 0.1 \times 10^{-1}$
pLac	pLac	0.993	$4.4 \pm 0.2 \times 10^{-2}$	$-4.7 \pm 0.3 \times 10^{-1}$	0.982	$4.8 \pm 0.3 \times 10^{-2}$	$-4.9 \pm 0.5 \times 10^{-1}$	0.998	$5.2 \pm 0.1 \times 10^{-2}$	$-2.7 \pm 0.2 \times 10^{-1}$	0.994	$4.2 \pm 0.1 \times 10^{-2}$	$-2.3 \pm 0.3 \times 10^{-1}$
pTrc	pLac	0.990	$4.8 \pm 0.3 \times 10^{-2}$	$-5.7 \pm 0.6 \times 10^{-1}$	0.989	$4.2 \pm 0.2 \times 10^{-2}$	$-4.6 \pm 0.3 \times 10^{-1}$	0.998	$4.5 \pm 0.1 \times 10^{-2}$	$-3.7 \pm 0.2 \times 10^{-1}$	0.998	$4.4 \pm 0.1 \times 10^{-2}$	$-4.0 \pm 0.2 \times 10^{-1}$
−pGFP	pNull	0.995	$4.4 \pm 0.1 \times 10^{-2}$	$-3.6 \pm 0.2 \times 10^{-1}$	0.992	$4.7 \pm 0.2 \times 10^{-2}$	$-4.1 \pm 0.3 \times 10^{-1}$	0.996	$5.0 \pm 0.1 \times 10^{-2}$	$-3.9 \pm 0.2 \times 10^{-1}$	0.994	$4.6 \pm 0.1 \times 10^{-2}$	$-3.5 \pm 0.3 \times 10^{-1}$
pLac	pNull	0.987	$4.3 \pm 0.2 \times 10^{-2}$	$-3.7 \pm 0.4 \times 10^{-1}$	0.991	$4.2 \pm 0.2 \times 10^{-2}$	$-3.7 \pm 0.3 \times 10^{-1}$	0.998	$5.4 \pm 0.1 \times 10^{-2}$	$-4.3 \pm 0.2 \times 10^{-1}$	0.998	$4.4 \pm 0.1 \times 10^{-2}$	$-3.3 \pm 0.2 \times 10^{-1}$
**Absorbance (X: λ_{abs} = 600 nm) *versus* GFP Fluorescence (Y: λ_{ex} = 485 nm, λ_{em} = 510 nm, gain = 50)**													
−pGFP	−pRFP	0.937	$9.4 \pm 1.2 \times 10^{1}$	$5.8 \pm 0.8 \times 10^{1}$	0.973	$10.1 \pm 0.8 \times 10^{1}$	$5.9 \pm 0.5 \times 10^{1}$	0.990	$12.7 \pm 0.6 \times 10^{1}$	$5.0 \pm 0.5 \times 10^{1}$	0.988	$11.8 \pm 0.6 \times 10^{1}$	$6.0 \pm 0.5 \times 10^{1}$
pUV5	−pRFP	0.979	$10.9 \pm 0.8 \times 10^{3}$	$-0.9 \pm 0.5 \times 10^{3}$	0.996	$12.1 \pm 0.4 \times 10^{3}$	$-1.3 \pm 0.2 \times 10^{3}$	0.993	$13.3 \pm 0.6 \times 10^{3}$	$-1.9 \pm 0.3 \times 10^{3}$	0.995	$13.4 \pm 0.5 \times 10^{3}$	$-1.6 \pm 0.3 \times 10^{3}$
pSym	−pRFP	0.978	$30.1 \pm 2.2 \times 10^{3}$	$-3.6 \pm 1.3 \times 10^{3}$	0.997	$33.6 \pm 0.9 \times 10^{3}$	$-5.4 \pm 0.5 \times 10^{3}$	0.992	$8.9 \pm 0.4 \times 10^{2}$	$-1.7 \pm 0.3 \times 10^{2}$	0.995	$10.5 \pm 0.4 \times 10^{3}$	$-1.7 \pm 0.2 \times 10^{3}$
pNull	−pRFP	0.981	$33.1 \pm 2.3 \times 10^{3}$	$-3.2 \pm 1.2 \times 10^{3}$	0.994	$32.5 \pm 1.3 \times 10^{3}$	$-3.0 \pm 0.8 \times 10^{3}$	0.997	$31.9 \pm 0.9 \times 10^{3}$	$-3.5 \pm 0.6 \times 10^{3}$	0.997	$31.0 \pm 0.8 \times 10^{3}$	$-2.8 \pm 0.5 \times 10^{3}$
pLac	−pRFP	0.968	$38.2 \pm 3.5 \times 10^{3}$	$-3.5 \pm 1.8 \times 10^{3}$	0.997	$40.0 \pm 1.1 \times 10^{3}$	$-4.2 \pm 0.6 \times 10^{3}$	0.970	$2.4 \pm 0.2 \times 10^{3}$	$-0.7 \pm 0.2 \times 10^{3}$	0.993	$27.8 \pm 1.2 \times 10^{3}$	$-0.3 \pm 0.8 \times 10^{3}$
pTrc	−pRFP	0.966	$48.4 \pm 5.2 \times 10^{3}$	$-2.9 \pm 2.4 \times 10^{3}$	0.996	$46.8 \pm 1.7 \times 10^{3}$	$-2.4 \pm 0.8 \times 10^{3}$	0.995	$49.3 \pm 2.0 \times 10^{3}$	$-5.3 \pm 1.1 \times 10^{3}$	0.999	$46.1 \pm 1.0 \times 10^{3}$	$-3.7 \pm 0.5 \times 10^{3}$
−pGFP	pLac	0.873	$8.2 \pm 1.4 \times 10^{1}$	$8.2 \pm 0.7 \times 10^{1}$	0.804	$5.7 \pm 1.4 \times 10^{1}$	$9.2 \pm 0.6 \times 10^{1}$	0.956	$9.1 \pm 1.0 \times 10^{1}$	$8.2 \pm 0.7 \times 10^{1}$	0.785	$5.6 \pm 1.5 \times 10^{1}$	$10.0 \pm 0.8 \times 10^{1}$
pUV5	pLac	0.994	$2.4 \pm 0.1 \times 10^{3}$	$2.9 \pm 5.1 \times 10^{1}$	0.987	$2.2 \pm 0.2 \times 10^{3}$	$-3.5 \pm 5.5 \times 10^{1}$	0.999	$12.3 \pm 0.2 \times 10^{3}$	$-17.5 \pm 0.9 \times 10^{2}$	0.997	$49.1 \pm 1.4 \times 10^{2}$	$-1.9 \pm 0.8 \times 10^{2}$
pSym	pLac	0.980	$7.8 \pm 0.6 \times 10^{3}$	$-3.2 \pm 2.0 \times 10^{2}$	0.996	$6.5 \pm 0.2 \times 10^{3}$	$-1.8 \pm 1.1 \times 10^{2}$	0.997	$5.2 \pm 0.2 \times 10^{2}$	$-2.3 \pm 0.8 \times 10^{1}$	0.957	$27.7 \pm 2.9 \times 10^{2}$	$0.8 \pm 1.4 \times 10^{2}$
pNull	pLac	0.989	$7.9 \pm 0.6 \times 10^{3}$	$-2.2 \pm 2.7 \times 10^{2}$	0.994	$7.1 \pm 0.3 \times 10^{3}$	$-0.8 \pm 1.2 \times 10^{2}$	0.994	$35.3 \pm 1.4 \times 10^{3}$	$-3.9 \pm 0.7 \times 10^{3}$	0.996	$15.0 \pm 0.5 \times 10^{3}$	$-9.6 \pm 2.2 \times 10^{2}$
pLac	pLac	0.992	$9.0 \pm 0.5 \times 10^{3}$	$-2.0 \pm 2.4 \times 10^{2}$	0.994	$7.5 \pm 0.6 \times 10^{3}$	$0.8 \pm 2.1 \times 10^{2}$	0.979	$12.6 \pm 0.9 \times 10^{2}$	$-2.1 \pm 0.7 \times 10^{2}$	0.955	$10.6 \pm 1.6 \times 10^{3}$	$3.0 \pm 1.2 \times 10^{3}$
pTrc	pLac	0.995	$7.1 \pm 0.8 \times 10^{3}$	$1.7 \pm 0.7 \times 10^{2}$	0.996	$8.9 \pm 0.4 \times 10^{3}$	$1.7 \pm 1.6 \times 10^{2}$	0.995	$48.2 \pm 2.0 \times 10^{3}$	$-5.0 \pm 1.1 \times 10^{3}$	0.997	$17.6 \pm 0.5 \times 10^{3}$	$1.2 \pm 1.7 \times 10^{2}$
−pGFP	pNull	0.892	$5.9 \pm 1.0 \times 10^{1}$	$9.5 \pm 0.5 \times 10^{1}$	0.904	$9.1 \pm 1.5 \times 10^{1}$	$8.3 \pm 0.8 \times 10^{1}$	0.887	$8.3 \pm 1.3 \times 10^{1}$	$8.5 \pm 0.7 \times 10^{1}$	0.765	$7.6 \pm 1.9 \times 10^{1}$	$8.3 \pm 0.9 \times 10^{1}$
pLac	pNull	0.993	$18.7 \pm 0.8 \times 10^{3}$	$-10.7 \pm 3.7 \times 10^{2}$	0.994	$17.7 \pm 0.7 \times 10^{3}$	$-10.6 \pm 3.2 \times 10^{2}$	0.996	$43.1 \pm 1.3 \times 10^{1}$	$4.1 \pm 0.9 \times 10^{1}$	0.987	$16.8 \pm 1.0 \times 10^{3}$	$-13.6 \pm 5.2 \times 10^{2}$
**Absorbance (X: λ_{abs} = 600 nm) *versus* RFP Fluorescence (Y: λ_{ex} = 585 nm, λ_{em} = 610 nm, gain = 50)**													
−pGFP	−pRFP	0.002	$0.1 \pm 1.5 \times 10^{1}$	$8.3 \pm 1.0 \times 10^{1}$	0.010	$0.3 \pm 1.7 \times 10^{1}$	$9.8 \pm 1.1 \times 10^{1}$	0.244	$-1.1 \pm 1.0 \times 10^{1}$	$10.1 \pm 0.7 \times 10^{1}$	0.331	$-1.9 \pm 1.3 \times 10^{1}$	$11.6 \pm 1.0 \times 10^{1}$
pUV5	−pRFP	0.308	$-2.3 \pm 2.0 \times 10^{1}$	$10.6 \pm 1.2 \times 10^{1}$	0.002	$0.1 \pm 0.6 \times 10^{1}$	$10.4 \pm 0.4 \times 10^{1}$	0.069	$0.6 \pm 1.2 \times 10^{1}$	$8.5 \pm 0.7 \times 10^{1}$	0.178	$2.5 \pm 2.7 \times 10^{1}$	$8.6 \pm 1.5 \times 10^{1}$
pSym	−pRFP	0.628	$-1.4 \pm 0.5 \times 10^{1}$	$10.3 \pm 0.3 \times 10^{1}$	0.004	$0.2 \pm 1.3 \times 10^{1}$	$10.4 \pm 0.8 \times 10^{1}$	0.025	$0.2 \pm 0.7 \times 10^{1}$	$9.7 \pm 0.5 \times 10^{1}$	0.001	$0.1 \pm 1.5 \times 10^{1}$	$10.7 \pm 1.0 \times 10^{1}$
pNull	−pRFP	0.053	$-0.8 \pm 1.7 \times 10^{1}$	$9.6 \pm 0.9 \times 10^{1}$	0.020	$-0.2 \pm 0.7 \times 10^{1}$	$10.6 \pm 0.4 \times 10^{1}$	0.015	$0.3 \pm 1.3 \times 10^{1}$	$9.9 \pm 0.9 \times 10^{1}$	0.329	$-1.6 \pm 1.2 \times 10^{1}$	$11.7 \pm 0.8 \times 10^{1}$
pLac	−pRFP	0.015	$-0.3 \pm 1.1 \times 10^{1}$	$9.8 \pm 0.6 \times 10^{1}$	0.000	$0.0 \pm 1.5 \times 10^{1}$	$10.6 \pm 0.9 \times 10^{1}$	0.245	$-1.6 \pm 1.6 \times 10^{1}$	$10.9 \pm 1.1 \times 10^{1}$	0.746	$-1.9 \pm 0.5 \times 10^{1}$	$12.2 \pm 0.4 \times 10^{1}$
pTrc	−pRFP	0.052	$2.1 \pm 5.1 \times 10^{1}$	$9.7 \pm 2.4 \times 10^{1}$	0.028	$-0.6 \pm 2.0 \times 10^{1}$	$11.1 \pm 0.9 \times 10^{1}$	0.006	$0.1 \pm 1.0 \times 10^{1}$	$10.3 \pm 0.5 \times 10^{1}$	0.003	$0.2 \pm 1.6 \times 10^{1}$	$11.1 \pm 0.8 \times 10^{1}$
−pGFP	pLac	0.984	$73.2 \pm 4.7 \times 10^{2}$	$-10.6 \pm 2.2 \times 10^{2}$	0.995	$75.0 \pm 2.8 \times 10^{2}$	$-9.1 \pm 1.3 \times 10^{2}$	0.980	$70.3 \pm 5.0 \times 10^{1}$	$-21.6 \pm 3.8 \times 10^{1}$	0.974	$50.4 \pm 4.1 \times 10^{2}$	$-14.0 \pm 2.5 \times 10^{2}$
pUV5	pLac	0.979	$61.2 \pm 4.5 \times 10^{2}$	$-6.8 \pm 1.9 \times 10^{2}$	0.994	$59.7 \pm 2.4 \times 10^{2}$	$-5.9 \pm 1.0 \times 10^{2}$	0.997	$4.4 \pm 0.1 \times 10^{2}$	$-4.9 \pm 0.8 \times 10^{1}$	0.969	$55.6 \pm 5.4 \times 10^{2}$	$-10.2 \pm 2.7 \times 10^{2}$
pSym	pLac	0.980	$49.4 \pm 3.5 \times 10^{2}$	$-5.4 \pm 1.6 \times 10^{2}$	0.995	$45.5 \pm 1.7 \times 10^{2}$	$-3.2 \pm 0.7 \times 10^{2}$	0.912	$2.9 \pm 0.5 \times 10^{2}$	$-0.3 \pm 2.5 \times 10^{1}$	0.926	$43.9 \pm 7.2 \times 10^{2}$	$-9.5 \pm 3.6 \times 10^{2}$
pNull	pLac	0.980	$44.1 \pm 3.2 \times 10^{2}$	$-3.6 \pm 1.2 \times 10^{2}$	0.992	$42.7 \pm 1.9 \times 10^{2}$	$-3.3 \pm 0.8 \times 10^{2}$	0.886	$2.0 \pm 0.4 \times 10^{2}$	$7.7 \pm 1.8 \times 10^{1}$	0.994	$48.9 \pm 2.3 \times 10^{2}$	$-5.5 \pm 1.2 \times 10^{2}$
pLac	pLac	0.981	$41.0 \pm 2.8 \times 10^{2}$	$-3.2 \pm 1.1 \times 10^{2}$	0.988	$32.8 \pm 1.8 \times 10^{2}$	$-1.1 \pm 0.8 \times 10^{2}$	0.989	$3.3 \pm 0.2 \times 10^{2}$	$-1.1 \pm 1.3 \times 10^{1}$	0.981	$33.4 \pm 2.3 \times 10^{2}$	$-7.0 \pm 1.3 \times 10^{2}$
pTrc	pLac	0.987	$16.7 \pm 1.4 \times 10^{2}$	$-1.1 \pm 4.8 \times 10^{1}$	0.994	$20.0 \pm 0.8 \times 10^{2}$	$-1.8 \pm 2.1 \times 10^{1}$	0.870	$9.9 \pm 2.2 \times 10^{1}$	$1.1 \pm 0.1 \times 10^{2}$	0.971	$20.8 \pm 2.1 \times 10^{2}$	$-9.6 \pm 8.6 \times 10^{1}$
−pGFP	pNull	0.993	$69.0 \pm 2.9 \times 10^{2}$	$-11.1 \pm 1.5 \times 10^{2}$	0.998	$67.2 \pm 1.4 \times 10^{2}$	$-93.5 \pm 7.2 \times 10^{1}$	0.973	$77.4 \pm 6.4 \times 10^{2}$	$-21.9 \pm 3.8 \times 10^{2}$	0.988	$63.0 \pm 3.5 \times 10^{2}$	$-13.4 \pm 1.9 \times 10^{2}$
pLac	pNull	0.997	$43.0 \pm 1.3 \times 10^{2}$	$-47.4 \pm 6.1 \times 10^{1}$	0.998	$44.3 \pm 1.0 \times 10^{2}$	$-46.3 \pm 4.7 \times 10^{1}$	0.981	$77.2 \pm 5.3 \times 10^{2}$	$-24.2 \pm 3.4 \times 10^{2}$	0.967	$37.3 \pm 3.4 \times 10^{2}$	$-6.9 \pm 1.8 \times 10^{2}$
**Absorbance (X: λ_{abs} = 600 nm) *versus* GFP-RFP Förster Resonance Energy Transfer (Y: λ_{ex} = 485 nm, λ_{em} = 610 nm, gain = 50)**													
−pGFP	−pRFP	0.984	$2.6 \pm 0.2 \times 10^{1}$	$0.0 \pm 0.2 \times 10^{1}$	0.998	$2.8 \pm 0.1 \times 10^{1}$	$-0.2 \pm 0.1 \times 10^{1}$	0.996	$3.6 \pm 0.1 \times 10^{1}$	$-0.4 \pm 0.1 \times 10^{1}$	0.996	$3.5 \pm 0.1 \times 10^{1}$	$-0.4 \pm 0.1 \times 10^{1}$
pUV5	−pRFP	0.986	$19.7 \pm 1.2 \times 10^{1}$	$-2.0 \pm 1.2 \times 10^{1}$	0.999	$21.8 \pm 0.4 \times 10^{1}$	$-2.8 \pm 0.4 \times 10^{1}$	0.997	$23.3 \pm 0.7 \times 10^{1}$	$-3.3 \pm 0.7 \times 10^{1}$	0.998	$23.2 \pm 0.6 \times 10^{1}$	$-2.9 \pm 0.6 \times 10^{1}$
pSym	−pRFP	0.989	$59.9 \pm 3.2 \times 10^{1}$	$-10.2 \pm 3.2 \times 10^{1}$	0.997	$68.9 \pm 1.8 \times 10^{1}$	$-14.7 \pm 1.8 \times 10^{1}$	0.999	$4.5 \pm 0.1 \times 10^{1}$	$-0.5 \pm 0.1 \times 10^{1}$	0.996	$19.6 \pm 0.6 \times 10^{1}$	$-3.4 \pm 0.6 \times 10^{1}$
pNull	−pRFP	0.993	$68.0 \pm 2.8 \times 10^{1}$	$-10.7 \pm 2.8 \times 10^{1}$	0.995	$68.8 \pm 2.3 \times 10^{1}$	$-11.3 \pm 2.3 \times 10^{1}$	0.992	$70.1 \pm 3.1 \times 10^{1}$	$-13.5 \pm 3.1 \times 10^{1}$	0.996	$68.7 \pm 2.3 \times 10^{1}$	$-12.4 \pm 2.3 \times 10^{1}$
pLac	−pRFP	0.985	$81.1 \pm 4.9 \times 10^{1}$	$-12.4 \pm 4.9 \times 10^{1}$	0.998	$85.5 \pm 2.0 \times 10^{1}$	$-14.1 \pm 2.0 \times 10^{1}$	0.986	$7.0 \pm 0.4 \times 10^{1}$	$-1.6 \pm 0.4 \times 10^{1}$	0.987	$64.0 \pm 3.7 \times 10^{1}$	$-9.0 \pm 3.7 \times 10^{1}$
pTrc	−pRFP	0.984	$11.1 \pm 0.8 \times 10^{2}$	$-1.5 \pm 0.8 \times 10^{2}$	1.000	$10.4 \pm 0.1 \times 10^{2}$	$-12.4 \pm 0.7 \times 10^{1}$	0.993	$11.5 \pm 0.6 \times 10^{2}$	$-2.1 \pm 0.6 \times 10^{2}$	0.998	$10.3 \pm 0.3 \times 10^{2}$	$-1.5 \pm 0.3 \times 10^{2}$
−pGFP	pLac	0.979	$53.1 \pm 3.9 \times 10^{1}$	$-8.5 \pm 3.9 \times 10^{1}$	0.994	$54.7 \pm 2.1 \times 10^{1}$	$-7.6 \pm 2.1 \times 10^{1}$	0.988	$8.3 \pm 0.5 \times 10^{1}$	$-2.9 \pm 0.5 \times 10^{1}$	0.977	$37.1 \pm 2.9 \times 10^{1}$	$-11.0 \pm 2.9 \times 10^{1}$
pUV5	pLac	0.975	$66.4 \pm 5.3 \times 10^{1}$	$-8.6 \pm 5.3 \times 10^{1}$	0.994	$64.4 \pm 2.6 \times 10^{1}$	$-7.5 \pm 2.6 \times 10^{1}$	0.999	$29.3 \pm 0.3 \times 10^{1}$	$-6.4 \pm 0.5 \times 10^{1}$	0.991	$94.8 \pm 4.6 \times 10^{1}$	$-25.1 \pm 4.6 \times 10^{1}$
pSym	pLac	0.963	$11.0 \pm 1.1 \times 10^{2}$	$-1.7 \pm 1.1 \times 10^{2}$	0.988	$9.2 \pm 0.5 \times 10^{2}$	$-9.5 \pm 5.0 \times 10^{1}$	0.969	$5.4 \pm 0.5 \times 10^{1}$	$-0.7 \pm 0.5 \times 10^{1}$	0.948	$4.9 \pm 0.7 \times 10^{2}$	$-1.0 \pm 0.7 \times 10^{2}$
pNull	pLac	0.970	$9.7 \pm 0.9 \times 10^{2}$	$-1.1 \pm 0.9 \times 10^{2}$	0.992	$9.5 \pm 0.4 \times 10^{2}$	$-1.1 \pm 0.4 \times 10^{2}$	0.999	$7.8 \pm 0.1 \times 10^{2}$	$-1.3 \pm 0.1 \times 10^{2}$	0.999	$16.4 \pm 0.3 \times 10^{2}$	$-2.9 \pm 0.3 \times 10^{2}$
pLac	pLac	0.968	$10.8 \pm 1.0 \times 10^{2}$	$-1.3 \pm 1.0 \times 10^{2}$	0.991	$90.5 \pm 4.3 \times 10^{1}$	$-7.9 \pm 4.3 \times 10^{1}$	0.994	$11.0 \pm 0.4 \times 10^{1}$	$-3.5 \pm 0.4 \times 10^{1}$	0.997	$12.1 \pm 0.3 \times 10^{2}$	$-2.9 \pm 0.3 \times 10^{2}$
pTrc	pLac	0.988	$65.8 \pm 4.1 \times 10^{1}$	$-4.9 \pm 4.1 \times 10^{1}$	0.983	$93.5 \pm 7.2 \times 10^{1}$	$-9.8 \pm 7.2 \times 10^{1}$	0.999	$12.4 \pm 0.2 \times 10^{2}$	$-2.5 \pm 0.2 \times 10^{2}$	0.994	$16.0 \pm 0.7 \times 10^{2}$	$-2.9 \pm 0.7 \times 10^{2}$
−pGFP	pNull	0.992	$51.1 \pm 2.3 \times 10^{1}$	$-9.3 \pm 2.3 \times 10^{1}$	0.999	$49.8 \pm 0.9 \times 10^{1}$	$-8.0 \pm 0.9 \times 10^{1}$	0.971	$56.3 \pm 4.9 \times 10^{1}$	$-16.4 \pm 4.9 \times 10^{1}$	0.987	$46.1 \pm 2.7 \times 10^{1}$	$-10.6 \pm 2.7 \times 10^{1}$
pLac	pNull	0.997	$16.2 \pm 0.4 \times 10^{2}$	$-26.5 \pm 4.1 \times 10^{1}$	0.999	$16.5 \pm 0.3 \times 10^{2}$	$-25.6 \pm 2.8 \times 10^{1}$	0.979	$59.6 \pm 4.4 \times 10^{1}$	$-19.1 \pm 4.4 \times 10^{1}$	0.986	$13.2 \pm 0.8 \times 10^{2}$	$-29.4 \pm 7.8 \times 10^{1}$

Table S2. pValue Statistics

Promoter Architecture		GFP ÷ Absorbance T-Test p-Value (n = 3, two-tailed homoscedastic)				RFP ÷ Absorbance T-Test p-Value (n = 3, two-tailed homoscedastic)			
		μ_1: −LacI / −IPTG	μ_1: −LacI / −IPTG	μ_2: −LacI / +IPTG	μ_3: +LacI / −IPTG	μ_1: −LacI / −IPTG	μ_1: −LacI / −IPTG	μ_2: −LacI / +IPTG	μ_3: +LacI / −IPTG
GFP	RFP	μ_2: −LacI / +IPTG	μ_3: +LacI / −IPTG	μ_4: +LacI / +IPTG	μ_4: +LacI / +IPTG	μ_2: −LacI / +IPTG	μ_3: +LacI / −IPTG	μ_4: +LacI / +IPTG	μ_4: +LacI / +IPTG
−pGFP	−pRFP	4.7×10^{-1}	1.5×10^{-2}	5.5×10^{-2}	1.4×10^{-1}	8.9×10^{-1}	3.0×10^{-1}	1.5×10^{-1}	4.6×10^{-1}
pUV5	−pRFP	8.0×10^{-2}	1.3×10^{-2}	1.8×10^{-2}	8.0×10^{-1}	1.2×10^{-1}	9.3×10^{-2}	2.0×10^{-1}	3.3×10^{-1}
pSym	−pRFP	6.7×10^{-2}	2.3×10^{-5}	1.8×10^{-6}	1.3×10^{-6}	1.4×10^{-1}	3.8×10^{-2}	9.7×10^{-1}	9.0×10^{-1}
pNull	−pRFP	7.2×10^{-1}	4.3×10^{-1}	1.7×10^{-1}	3.0×10^{-1}	6.0×10^{-1}	4.1×10^{-1}	1.4×10^{-1}	1.3×10^{-1}
pLac	−pRFP	4.2×10^{-1}	5.7×10^{-5}	1.9×10^{-4}	3.3×10^{-6}	7.8×10^{-1}	3.0×10^{-1}	1.0×10^{-1}	7.7×10^{-1}
pTrc	−pRFP	6.4×10^{-1}	8.0×10^{-1}	5.5×10^{-1}	6.2×10^{-2}	4.5×10^{-1}	5.5×10^{-1}	6.4×10^{-1}	9.8×10^{-1}
−pGFP	pLac	9.5×10^{-2}	4.3×10^{-1}	9.2×10^{-1}	2.6×10^{-2}	6.1×10^{-1}	1.7×10^{-5}	9.9×10^{-4}	5.4×10^{-5}
pUV5	pLac	1.1×10^{-1}	8.5×10^{-8}	2.2×10^{-5}	4.1×10^{-7}	6.4×10^{-1}	2.6×10^{-5}	3.0×10^{-1}	8.0×10^{-5}
pSym	pLac	2.8×10^{-2}	3.8×10^{-5}	7.0×10^{-5}	1.9×10^{-4}	1.6×10^{-1}	2.2×10^{-5}	7.2×10^{-1}	5.9×10^{-4}
pNull	pLac	1.2×10^{-1}	6.3×10^{-6}	1.6×10^{-5}	1.8×10^{-5}	5.5×10^{-1}	2.2×10^{-5}	2.2×10^{-2}	3.8×10^{-6}
pLac	pLac	2.4×10^{-2}	9.4×10^{-6}	3.3×10^{-2}	5.6×10^{-4}	1.4×10^{-2}	2.2×10^{-5}	7.8×10^{-1}	2.4×10^{-5}
pTrc	pLac	2.5×10^{-2}	4.5×10^{-6}	1.4×10^{-5}	1.2×10^{-5}	2.0×10^{-2}	2.7×10^{-5}	5.8×10^{-1}	8.0×10^{-5}
−pGFP	pNull	3.5×10^{-2}	6.5×10^{-2}	3.2×10^{-1}	6.0×10^{-1}	3.9×10^{-1}	1.1×10^{-1}	1.3×10^{-1}	2.7×10^{-2}
pLac	pNull	1.8×10^{-1}	2.1×10^{-6}	2.6×10^{-1}	8.5×10^{-6}	2.1×10^{-1}	4.2×10^{-4}	2.7×10^{-2}	4.0×10^{-4}

Table S3. Regression statistics

Experimental Conditions		Time (X) *versus* Absorbance (Y)			Absorbance (X) *versus*			Absorbance (X) *versus*			Absorbance (X) *versus* GFP-RFP Förster Resonance Energy Transfer (Y)		
LacI	IPTG	R^2	Slope	Intercept	R^2	Slope	Intercept	R^2	Slope	Intercept	R^2	Slope	Intercept
**Promoter Architecture: −pGFP / −pRFP**													
$LacI_{TAN}$	0 mM	0.996	$4.7 \pm 0.1 \times 10^{-2}$	$-1.8 \pm 0.2 \times 10^{-1}$	0.978	$11.4 \pm 0.9 \times 10^{1}$	$4.4 \pm 0.6 \times 10^{1}$	0.106	$-0.6 \pm 0.9 \times 10^{1}$	$9.6 \pm 0.6 \times 10^{1}$	0.992	$3.1 \pm 0.1 \times 10^{1}$	$-0.4 \pm 0.1 \times 10^{1}$
$LacI_{TAN}$	10 mM	0.911	$4.7 \pm 0.6 \times 10^{-2}$	$-2.6 \pm 1.2 \times 10^{-1}$	0.895	$8.4 \pm 1.4 \times 10^{1}$	$6.3 \pm 0.9 \times 10^{1}$	0.002	$-0.1 \pm 1.7 \times 10^{1}$	$10.0 \pm 1.1 \times 10^{1}$	0.895	$2.4 \pm 0.4 \times 10^{1}$	$0.0 \pm 0.4 \times 10^{1}$
**Promoter Architecture: −pGFP / pLac RFP**													
$LacI_{TAN}$	0 mM	0.991	$6.4 \pm 0.2 \times 10^{-2}$	$-5.9 \pm 0.5 \times 10^{-1}$	0.960	$6.4 \pm 0.7 \times 10^{1}$	$8.7 \pm 0.4 \times 10^{1}$	0.988	$74.4 \pm 4.7 \times 10^{2}$	$-25.6 \pm 3.5 \times 10^{2}$	0.952	$47.1 \pm 5.3 \times 10^{1}$	$-13.3 \pm 5.3 \times 10^{1}$
$LacI_{TAN}$	10 mM	0.999	$5.2 \pm 0.1 \times 10^{-2}$	$-4.6 \pm 0.1 \times 10^{-1}$	0.958	$4.1 \pm 0.4 \times 10^{1}$	$8.8 \pm 0.3 \times 10^{1}$	0.998	$71.2 \pm 1.7 \times 10^{2}$	$-95.5 \pm 9.2 \times 10^{1}$	0.996	$52.8 \pm 1.7 \times 10^{1}$	$-8.3 \pm 1.7 \times 10^{1}$
**Promoter Architecture: −pGFP / pTan RFP**													
−LacI	0 mM	0.992	$4.7 \pm 0.2 \times 10^{-2}$	$-3.3 \pm 0.3 \times 10^{-1}$	0.456	$6.3 \pm 3.4 \times 10^{1}$	$9.9 \pm 2.0 \times 10^{1}$	0.987	$71.8 \pm 4.1 \times 10^{2}$	$-15.4 \pm 2.4 \times 10^{2}$	0.982	$52.8 \pm 3.6 \times 10^{1}$	$-12.2 \pm 3.6 \times 10^{1}$
−LacI	10 mM	0.995	$4.7 \pm 0.1 \times 10^{-2}$	$-3.9 \pm 0.3 \times 10^{-1}$	0.800	$4.6 \pm 1.2 \times 10^{1}$	$9.4 \pm 0.6 \times 10^{1}$	0.990	$68.0 \pm 3.3 \times 10^{2}$	$-10.2 \pm 1.8 \times 10^{2}$	0.991	$49.5 \pm 2.3 \times 10^{1}$	$-8.2 \pm 2.3 \times 10^{1}$
LacI	0 mM	0.994	$4.7 \pm 0.1 \times 10^{-2}$	$-4.2 \pm 0.3 \times 10^{-1}$	0.898	$10.4 \pm 1.7 \times 10^{1}$	$7.8 \pm 0.9 \times 10^{1}$	0.955	$68.3 \pm 7.5 \times 10^{2}$	$-18.1 \pm 4.4 \times 10^{2}$	0.937	$33.8 \pm 4.4 \times 10^{1}$	$-5.4 \pm 4.4 \times 10^{1}$
LacI	10 mM	0.996	$4.0 \pm 0.1 \times 10^{-2}$	$-3.7 \pm 0.2 \times 10^{-1}$	0.784	$9.9 \pm 2.6 \times 10^{1}$	$7.8 \pm 1.1 \times 10^{1}$	0.987	$67.3 \pm 7.8 \times 10^{2}$	$-22.0 \pm 4.7 \times 10^{2}$	0.976	$19.9 \pm 1.6 \times 10^{1}$	$-0.6 \pm 1.6 \times 10^{1}$
$LacI_{TAN}$	0 mM	0.991	$5.0 \pm 0.2 \times 10^{-2}$	$-1.9 \pm 0.4 \times 10^{-1}$	0.984	$11.3 \pm 0.7 \times 10^{1}$	$6.9 \pm 0.6 \times 10^{1}$	0.978	$73.2 \pm 5.5 \times 10^{1}$	$-20.4 \pm 4.3 \times 10^{1}$	0.985	$8.8 \pm 0.5 \times 10^{1}$	$-3.1 \pm 0.5 \times 10^{1}$
$LacI_{TAN}$	10 mM	0.997	$4.6 \pm 0.1 \times 10^{-2}$	$-2.5 \pm 0.2 \times 10^{-1}$	0.964	$6.5 \pm 0.6 \times 10^{1}$	$8.7 \pm 0.4 \times 10^{1}$	0.999	$60.1 \pm 1.1 \times 10^{2}$	$-12.2 \pm 0.7 \times 10^{2}$	0.998	$45.0 \pm 0.9 \times 10^{1}$	$-10.3 \pm 0.9 \times 10^{1}$

Table S4. pValue Statistics

T-Test Populations		GFP ÷ Absorbance p-Values (n = 3, two-tailed homoscedastic)			RFP ÷ Absorbance p-Values (n = 3, two-tailed homoscedastic)		
Population 1	Population 2	−pGFP / −pRFP	−pGFP / pLac RFP	−pGFP / pTan RFP	−pGFP / −pRFP	−pGFP / pLac RFP	−pGFP / pTan RFP
−LacI / −IPTG	−LacI / +IPTG	4.7×10^{-1}	9.5×10^{-2}	4.6×10^{-1}	8.9×10^{-1}	6.1×10^{-1}	2.9×10^{-1}
+LacI / −IPTG	+LacI / +IPTG	1.4×10^{-1}	2.6×10^{-2}	8.3×10^{-1}	4.6×10^{-1}	5.4×10^{-5}	8.8×10^{-1}
$+LacI_{TAN}$ / −IPTG	$+LacI_{TAN}$ / +IPTG	3.6×10^{-2}	1.0×10^{-2}	4.5×10^{-1}	7.0×10^{-1}	3.2×10^{-1}	2.0×10^{-7}
−LacI / −IPTG	+LacI / −IPTG	1.5×10^{-2}	4.3×10^{-1}	1.4×10^{-1}	3.0×10^{-1}	1.7×10^{-5}	5.3×10^{-1}
−LacI / −IPTG	$+LacI_{TAN}$ / −IPTG	8.7×10^{-2}	1.1×10^{-1}	7.2×10^{-2}	5.0×10^{-1}	7.7×10^{-1}	1.1×10^{-5}
+LacI / −IPTG	$+LacI_{TAN}$ / −IPTG	9.4×10^{-2}	1.9×10^{-2}	4.6×10^{-1}	5.5×10^{-1}	1.6×10^{-5}	1.4×10^{-4}
−LacI / +IPTG	+LacI / +IPTG	5.5×10^{-2}	9.2×10^{-1}	3.2×10^{-2}	1.5×10^{-1}	9.9×10^{-4}	9.0×10^{-1}
−LacI / +IPTG	$+LacI_{TAN}$ / +IPTG	1.5×10^{-1}	1.3×10^{-1}	6.8×10^{-2}	7.5×10^{-1}	1.1×10^{-1}	1.7×10^{-2}
+LacI / +IPTG	$+LacI_{TAN}$ / +IPTG	2.0×10^{-2}	1.6×10^{-1}	9.2×10^{-2}	2.4×10^{-1}	1.2×10^{-3}	1.9×10^{-1}

Figure S3

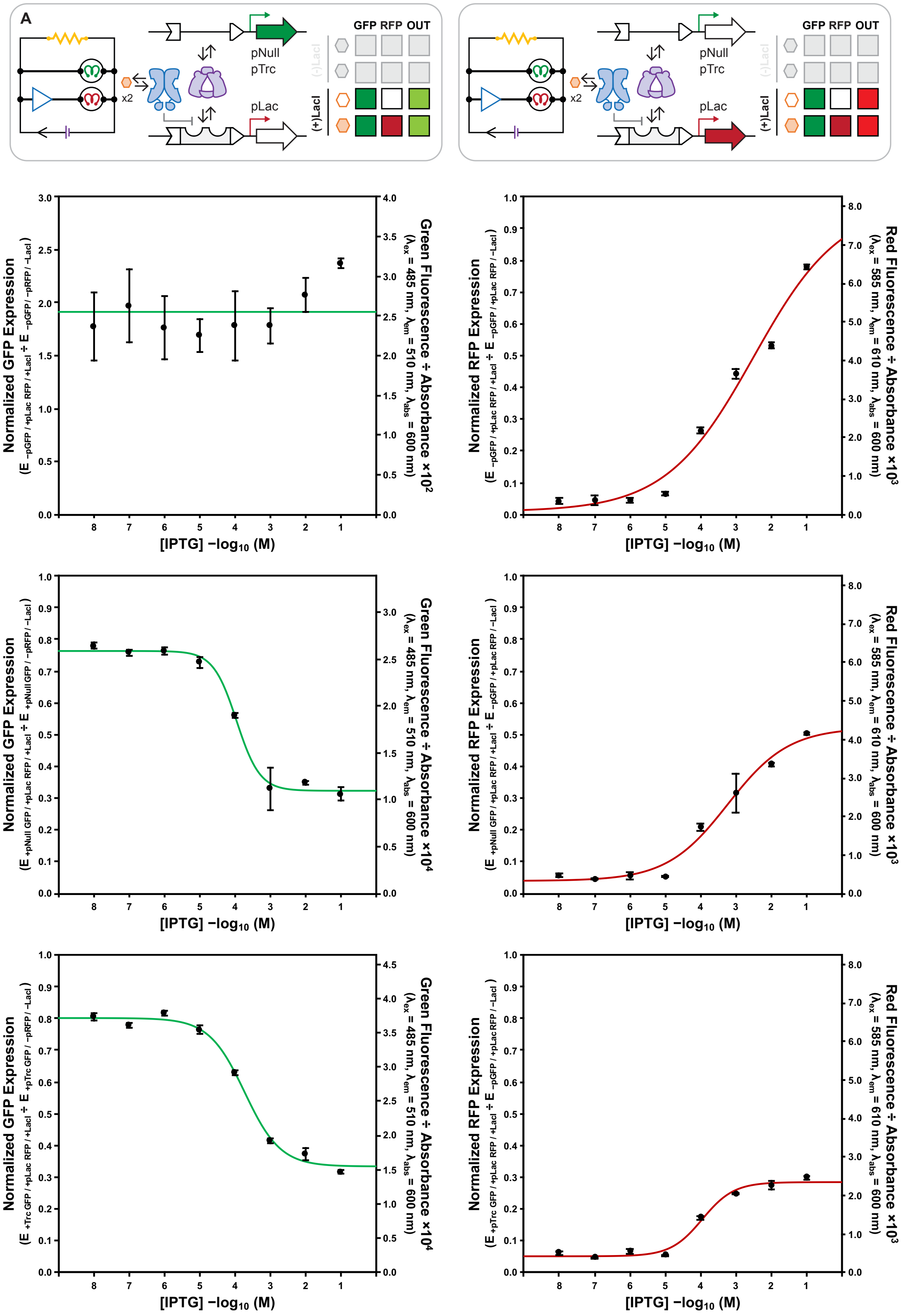

A
x2
pNull
pTrc
pLac
GFP RFP OUT
(-)LacI
(+)LacI
Normalized GFP Expression
Normalized RFP Expression
Green Fluorescence ÷ Absorbance ×10^2
Green Fluorescence ÷ Absorbance ×10^4
Red Fluorescence ÷ Absorbance ×10^3
(λex = 485 nm, λem = 510 nm, λabs = 600 nm)
(λex = 585 nm, λem = 610 nm, λabs = 600 nm)
[IPTG] −log10 (M)

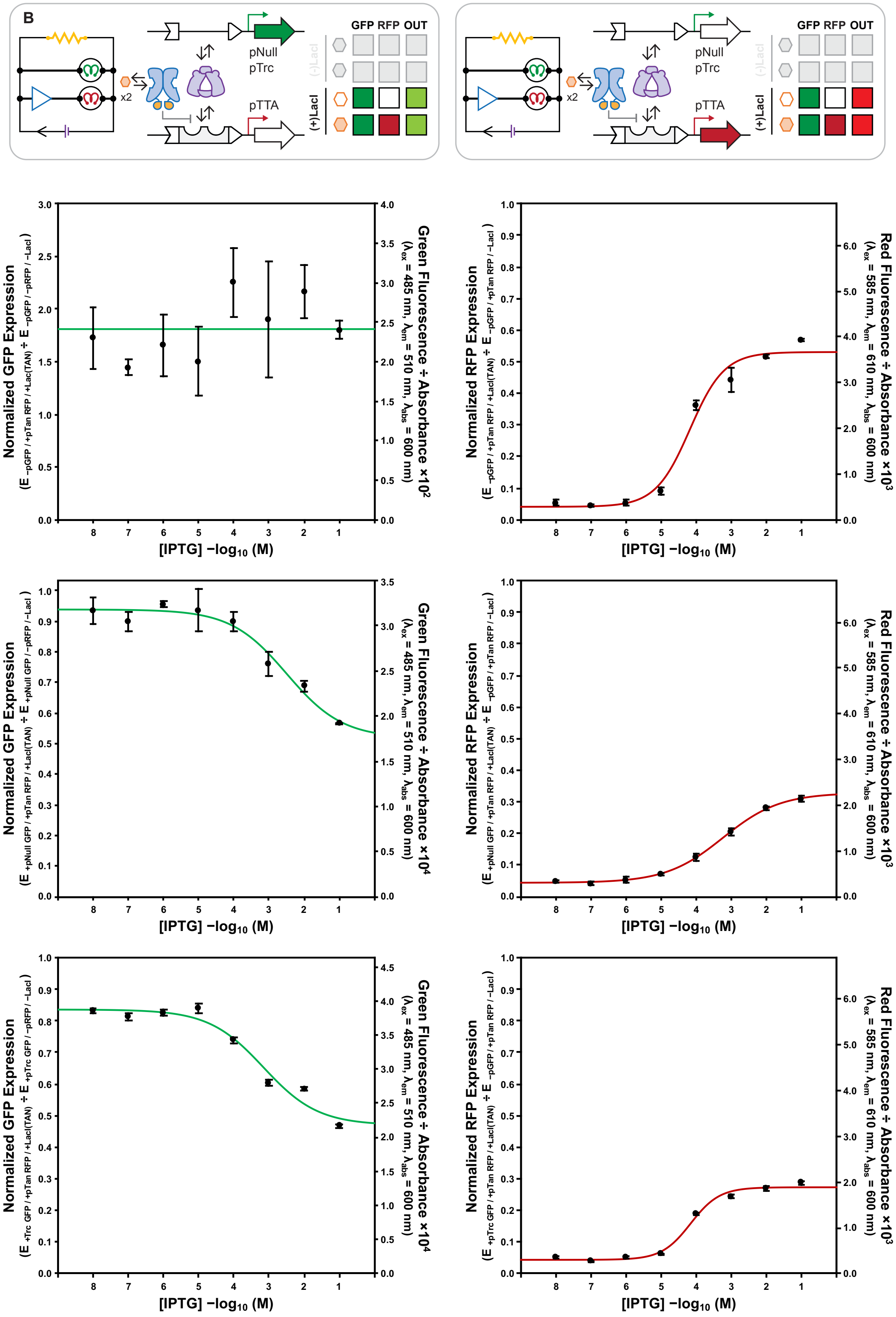
B
GFP RFP OUT
pNull
pTrc
pTTA
x2
(-)LacI
(+)LacI
Normalized GFP Expression
Normalized RFP Expression
Green Fluorescence ÷ Absorbance ×10²
Green Fluorescence ÷ Absorbance ×10⁴
Red Fluorescence ÷ Absorbance ×10³
(λex = 485 nm, λem = 510 nm, λabs = 600 nm)
(λex = 585 nm, λem = 610 nm, λabs = 600 nm)
[IPTG] −log10 (M)

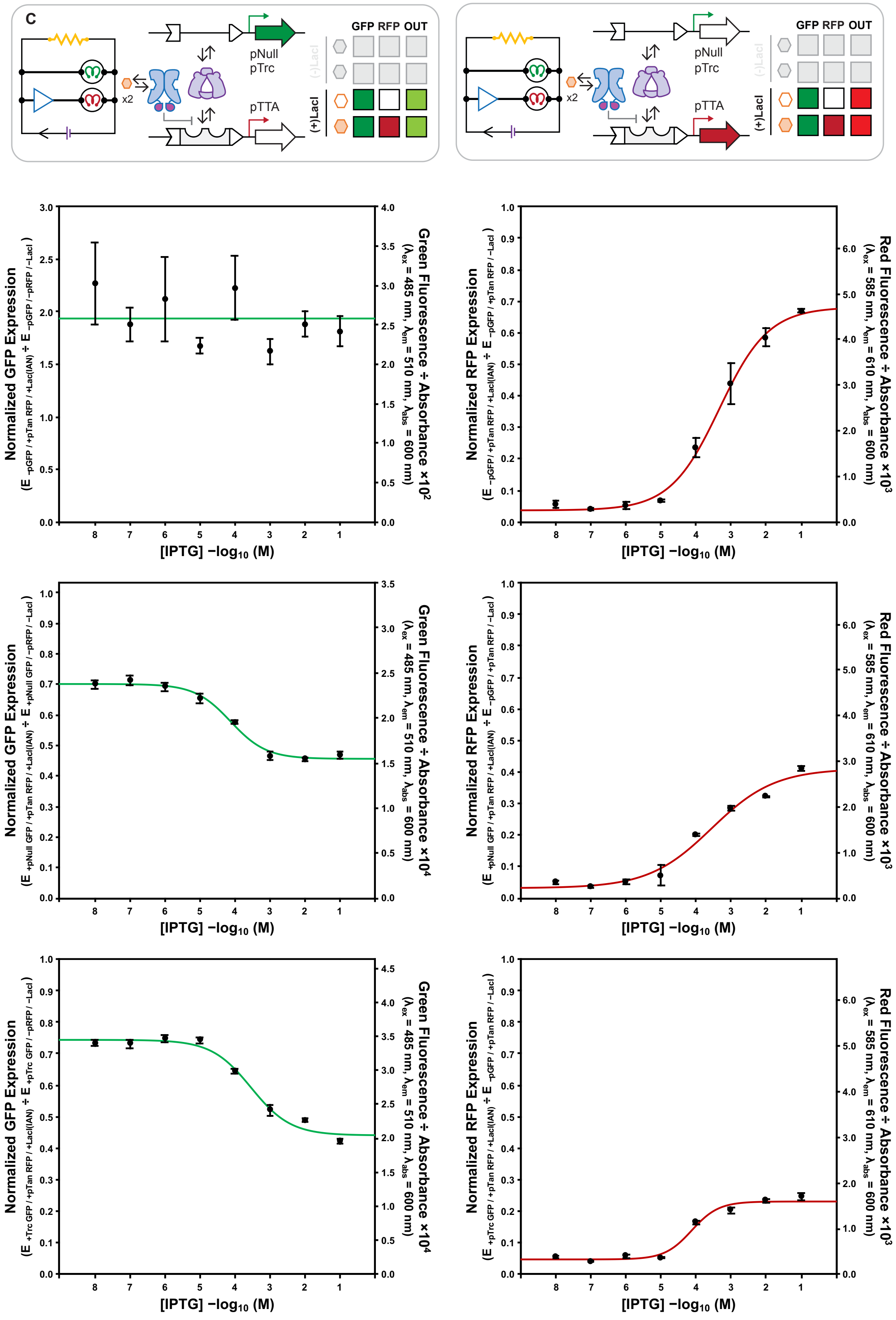

C
pNull
pTrc
pTTA
x2
GFP RFP OUT
(-)LacI
(+)LacI
Normalized GFP Expression
Green Fluorescence ÷ Absorbance ×10^2
(λex = 485 nm, λem = 510 nm, λabs = 600 nm)
Normalized RFP Expression
Red Fluorescence ÷ Absorbance ×10^3
(λex = 585 nm, λem = 610 nm, λabs = 600 nm)
Green Fluorescence ÷ Absorbance ×10^4
[IPTG] −log10 (M)

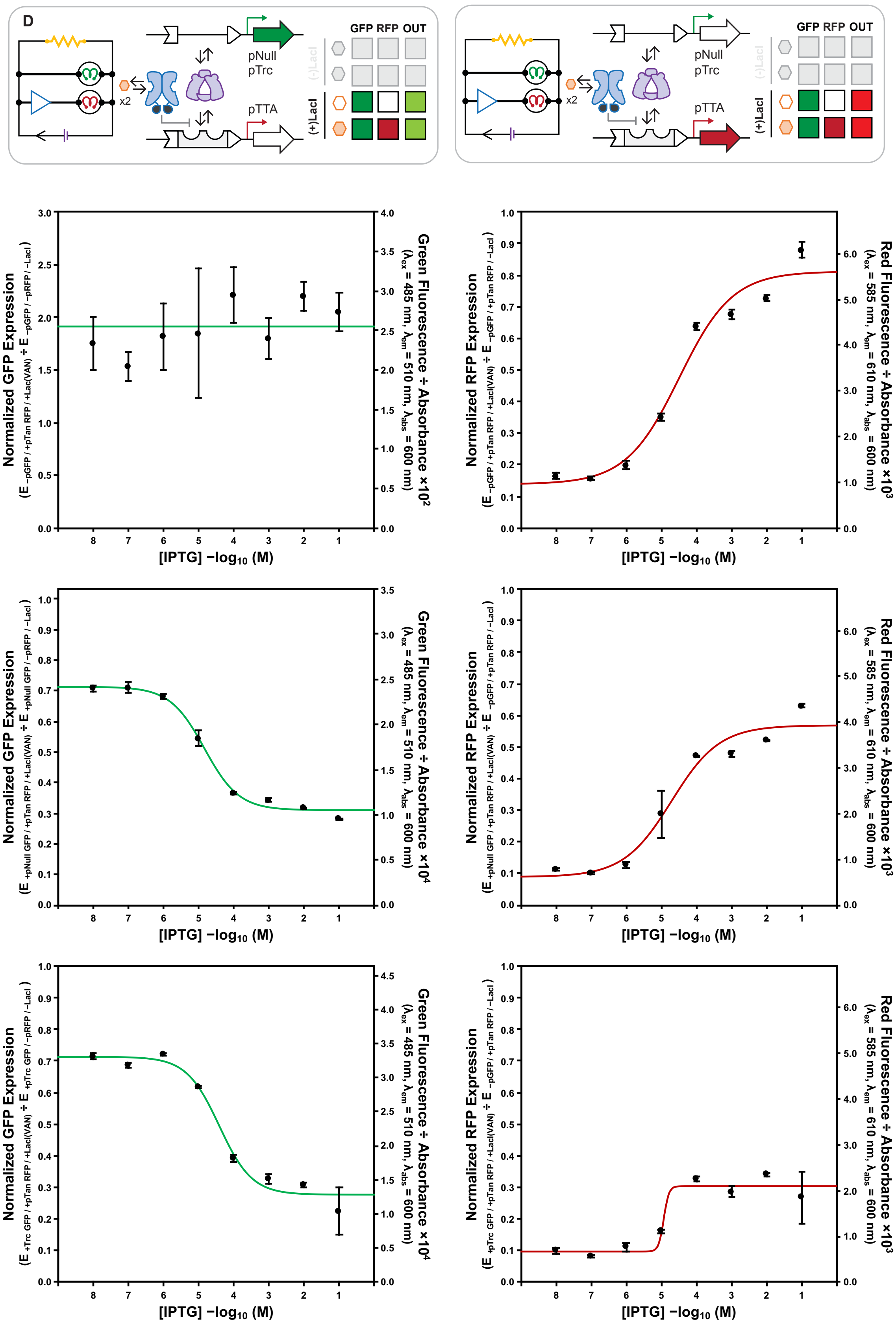
D
x2
pNull
pTrc
pTTA
GFP RFP OUT
(-)LacI
(+)LacI
Normalized GFP Expression
Normalized RFP Expression
Green Fluorescence ÷ Absorbance ×10²
Green Fluorescence ÷ Absorbance ×10⁴
Red Fluorescence ÷ Absorbance ×10³
(λex = 485 nm, λem = 510 nm, λabs = 600 nm)
(λex = 585 nm, λem = 610 nm, λabs = 600 nm)
[IPTG] −log10 (M)

Figure S3. Density normalized GFP and RFP expression measured at the 20 hour time-point as a function of various IPTG concentrations. Plots shown include: the repressor LacI(YQR) (A) co-expressed with the reporter plasmids –pGFP / pLac RFP, pNull GFP / pLac RFP, and pTrc GFP / pLac RFP, as well as the repressors LacI(TAN) (B), LacI(IAN) (C), and LacI(VAN) (D) co-expressed with the reporter plasmids –pGFP / pTTA RFP, pNull GFP / pTTA RFP, and pTrc GFP / pTTA RFP.

Table S5. IPTG Inducer Titration Curve Parameters

Experiment			Regression			Parameters			
pGFP	pRFP	LacI	Channel	R^2	$\|\|\varepsilon\|\|$	$-EC_{50}$	E_0	D	k
−pGFP	pLac	$LacI_{YQR}$	G/A	0.000	0.3620	0.000	1.697	0.000	0.000
−pGFP	pLac	$LacI_{YQR}$	R/A	0.980	0.0112	2.504	0.009	0.969	0.810
pNull	pLac	$LacI_{YQR}$	G/A	0.995	0.0016	3.960	0.323	0.439	−3.025
pNull	pLac	$LacI_{YQR}$	R/A	0.985	0.0035	3.227	0.038	0.485	1.162
pTrc	pLac	$LacI_{YQR}$	G/A	0.994	0.0019	3.730	0.333	0.468	−1.973
pTrc	pLac	$LacI_{YQR}$	R/A	0.986	0.0011	3.946	0.053	0.232	2.249
−pGFP	pTan	$LacI_{IAN}$	G/A	0.000	0.4214	0.000	1.618	0.000	0.000
−pGFP	pTan	$LacI_{IAN}$	R/A	0.997	0.0016	3.344	0.039	0.642	1.428
pNull	pTan	$LacI_{IAN}$	G/A	0.991	0.0008	4.115	0.455	0.247	−1.966
pNull	pTan	$LacI_{IAN}$	R/A	0.981	0.0029	3.565	0.033	0.379	1.114
pTrc	pTan	$LacI_{IAN}$	G/A	0.983	0.0021	3.530	0.441	0.302	−1.773
pTrc	pTan	$LacI_{IAN}$	R/A	0.982	0.0010	4.133	0.048	0.185	2.604
−pGFP	pTan	$LacI_{TAN}$	G/A	0.000	0.5917	0.000	1.416	0.000	0.000
−pGFP	pTan	$LacI_{TAN}$	R/A	0.984	0.0059	4.182	0.041	0.490	2.044
pNull	pTan	$LacI_{TAN}$	G/A	0.972	0.0040	2.502	0.520	0.417	−1.259
pNull	pTan	$LacI_{TAN}$	R/A	0.999	0.0000	3.241	0.043	0.285	1.230
pTrc	pTan	$LacI_{TAN}$	G/A	0.962	0.0054	3.163	0.470	0.365	−1.289
pTrc	pTan	$LacI_{TAN}$	R/A	0.991	0.0007	4.162	0.044	0.229	2.429
−pGFP	pTan	$LacI_{VAN}$	G/A	0.000	0.3896	0.000	1.562	0.000	0.000
−pGFP	pTan	$LacI_{VAN}$	R/A	0.972	0.0166	4.501	0.137	0.673	1.321
pNull	pTan	$LacI_{VAN}$	G/A	0.994	0.0015	4.832	0.309	0.405	−2.033
pNull	pTan	$LacI_{VAN}$	R/A	0.967	0.0103	4.721	0.090	0.478	1.464
pTrc	pTan	$LacI_{VAN}$	G/A	0.981	0.0056	4.410	0.275	0.437	−2.146
pTrc	pTan	$LacI_{VAN}$	R/A	0.952	0.0039	4.941	0.097	0.208	13.813

1. Garcia, H.G., Sanchez, A., Boedicker, J.Q., Osborne, M., Gelles, J., Kondev, J. and Phillips, R. (2012) Operator sequence alters gene expression independently of transcription factor occupancy in bacteria. *Cell Rep*, **2**, 150-161.
